# Community-based, peer-led psychosocial support to address stigma and reduce depression among adults with tuberculosis in Indonesia: A prospective interventional cohort study

**DOI:** 10.1371/journal.pgph.0006754

**Published:** 2026-07-16

**Authors:** Ahmad Fuady, Marinda Asiah Nuril Haya, Mariska Anindhita, Matsna Haniifah, Fathinah Ranggauni Hardy, Dzul Faridah Arinal Haq, Imelda Aliska, Elvira Radhiatul Febriani, Adji Fauzan Rifky, Artasya Karnasih, Feranindhya Agiananda, Finny Fitry Yani, Trevino Aristarkus Pakasi, Adhityawarman Menaldi, Budi Hermawan, Tom Wingfield

**Affiliations:** 1 Department of Community Medicine, Faculty of Medicine, Universitas Indonesia, Jakarta, Indonesia; 2 Primary Health Care Research and Innovation Centre, Indonesian Medical Education and Research Institute, Faculty of Medicine Universitas Indonesia, Jakarta, Indonesia; 3 Health Sciences Faculty, Universitas Pembangunan Nasional Veteran, Jakarta, Indonesia; 4 Department of Child Health, Faculty of Medicine, Universitas Andalas, Padang, West Sumatera, Indonesia; 5 Department of Psychiatry, Faculty of Medicine, Universitas Indonesia, Jakarta, Indonesia; 6 Faculty of Psychology, Universitas Indonesia, Depok, West Java, Indonesia; 7 Perhimpunan Organisasi Pasien Tuberkulosis (POP-TB) Indonesia, Jakarta, Indonesia; 8 Centre for Tuberculosis Research, Departments of Clinical Sciences and International Public Health, Liverpool School of Tropical Medicine, Liverpool, United Kingdom; 9 Department of Global Public Health, Karolinska Institute, Stockholm, Sweden; 10 Tropical and Infectious Disease Unit, Royal Liverpool and Broadgreen University Hospitals NHS Trust, Liverpool, United Kingdom; University of Sydney, AUSTRALIA

## Abstract

We co-developed and evaluated a community-based, peer-led psychosocial support intervention consisting of individual and peer-led group counselling, online group messaging, and community-based “TB-Talks” to address tuberculosis (TB)-Stigma and reduce depression among adults with TB in Indonesia. A non-randomized, single-arm prospective interventional cohort study was conducted in five primary healthcare centres and two public hospitals in Depok and Padang cities from June 2024 to January 2025. All newly-diagnosed individuals with either drug-sensitive (DS-) or drug-resistant (DR-) TB, aged 15 years and above, were consecutively recruited and offered individual counselling at baseline, then monthly peer-led group counselling and online group messaging and TB Talks during six months of treatment. Participants were assessed at baseline, end of intensive phase (Month-2), and continuation phase (Month-6) for TB-stigma, depression, and quality of life (QoL), using locally-adapted and validated tools including the Van Rie TB-Stigma scale, PHQ-9, and EQ5D5L, respectively. In addition, we collated quantitative acceptability, appropriateness and feasibility feedback from participants and stakeholders. A total of 129 people with TB (Depok = 85, Padang = 44) participated, including 55 (43%) women. Most participants had new DS-TB (62%), 19 (15%) recurrent DS-TB, and 30 (23%) DR-TB. At baseline, most participants had moderate-to-severe TB-Stigma (74%; 95%CI = 65–82%) and depression symptoms (57%; 35–72%). Among 112 participants who completed all assessments, moderate-to-severe TB-Stigma decreased to 43% (34–53%) and 21% (13–28%) and depression symptom prevalence decreased to 32% (20–47%) and 15% (6–23%) at Month-2 and Month-6, respectively. Participants’ QoL increased from median 0.84 (0.63-1.00, at baseline) to 0.92 (0.83-1.00, Month-2) and 1.00 (0.91-1.00, Month-6). Both study participants and stakeholders perceived that activities in this study were feasible, acceptable, and locally-appropriate. Community-based, peer-led psychosocial support, combining individual and monthly group counselling, has potential to mitigate TB-Stigma and depression while improving QoL among adults with TB in Indonesia.

## Background

It is estimated that tuberculosis (TB) caused illness in 10.7 million people and 1.23 million deaths globally in 2024 [1]. People with TB face massive challenges related to the psychosocial consequences of this poverty-related disease, including depression and TB-related stigma (herein termed TB-Stigma). TB-Stigma can discourage individuals experiencing symptoms from seeking medical care [2], resulting in delayed diagnosis and treatment initiation [3]. Even after diagnosis, TB-Stigma can make it more difficult for people with TB to take regularly and complete their treatment [4], undermining treatment success and contributing to poorer health, psychological and social (psychosocial), and economic outcomes [5].

Our previous “TB-CAPITA” study involving 612 people with TB across seven provinces of Indonesia found that 61% of individuals with TB experienced at least moderate TB-Stigma [6]. Moreover, experiencing TB-Stigma was significantly associated with symptoms of depression and a lower quality of life (QoL). These outcomes may be explained by the fact that TB-Stigma is often accompanied by discriminatory attitudes and behaviours, which can lead to broader social consequences such as isolation and loss of employment or income—factors that may contribute to the onset of depression and diminished QoL [5].

Designing and implementing psychosocial interventions to address TB-Stigma and depression is essential. To ensure the replicability and sustainability of such interventions, it is critical that they are grounded in TB-affected communities’ needs, driven by these same communities, and are implementable and scalable within available human resources and funding. Moreover, among TB-affected community groups, peers (individuals with lived experience of, or who have survived, the disease) [7], can serve as a vital bridge between communities and the health system, encouraging people with TB to seek care and facilitating navigation of the often-complex treatment journey [8, 9]. Peers are also ideally placed to be trained to deliver holistic support for people with TB to mitigate the stark psychosocial consequences associated with this disease of poverty. However, evidence on the effectiveness and cost-effectiveness of peer-based interventions to reduce TB-Stigma and depression, particularly those delivered in community settings, remains limited.

As part of the Medical Research Council UK Public Health Intervention Development (MRC PHIND) funded “Community-based, peer-led intervention to reduce TB-Stigma in Indonesia (TB-CAPS)” project, we engaged a diverse range of stakeholders to participate in the co-design and delivery of a psychosocial support intervention for people with TB [10]. The final intervention, reached through consensus, included a package comprising individual psychological assessment and counselling, monthly peer-led group counselling, online group messaging, and community-based “TB Talks.”

This study, within the TB-CAPS project, aimed to implement and evaluate the impact of the co-designed intervention on psychosocial outcomes important to people with TB including mitigating TB-Stigma, reducing depression, and improving QoL. Quantitative feedback on the feasibility, appropriateness, acceptability, and sustainability of the intervention was collected from key stakeholders including people with TB and National TB Programme (NTP) healthcare workers and leaders and evaluated.

## Methods

### Study design

This was a non-randomized, single-arm prospective interventional cohort study. Two cities—Depok and Padang—were purposively selected based on findings from our previous TB-CAPITA study [6], which identified people with TB in these cities as having substantial burdens of both TB-Stigma and depression. In each city, we collaborated with the local health offices to designate five primary health centres (three in Depok and two in Padang) and two hospitals providing drug-resistant TB services—Universitas Indonesia Hospital in Depok and Dr. M. Djamil Hospital in Padang—as study sites. The participant recruitment took place from June 12, 2024 to September 5, 2024, and follow up continued until January 16, 2025.

We applied a mixed-methods approach to evaluate the community-based, peer-led psychosocial support intervention through three key activities. First, we conducted longitudinal quantitative assessments of people with TB participating in the study to measure changes in TB-Stigma, depression scores, and QoL over time. Second, we engaged diverse stakeholders (see S1 File) to gather their perspectives on the feasibility, appropriateness, and acceptability of the TB-CAPS “active ingredients” —activities in our intervention that potentially drive clinical effect, are conceptually well defined, and link to specific hypothesised mechanisms of action [11]—as well as how such support could be funded, formulated, refined, and scaled up nationally in Indonesia. Third, we carried out in-depth interviews (IDIs) and focus group discussions (FGDs) with staff of provincial and district health offices, staff of primary health centres (called *Puskesmas* in Bahasa Indonesia), peers, and study participants to explore challenges, enabling factors, and the potential for replication and sustainability of the intervention. This paper reports the quantitative evaluation of TB-Stigma, depression, and QoL, and quantitative feedback from key stakeholders. The qualitative IDI and FGD findings will be reported elsewhere.

### Psychosocial support intervention

#### Population.

The intervention population included young people and adults, aged 15 years or above, newly diagnosed with drug-sensitive TB (DS-TB) notified to *Puskesmas* or with drug-resistant TB (DR-TB) notified to DR-TB health services at the two public hospital sites. Individuals with a pre-existing formal, documented diagnosis of an active mental health condition by the time of recruitment, including severe depression or psychosis, were excluded. Participants were consecutively invited and recruited based on their date of TB notification.

### Sample size calculation

Our *a priori* sample size calculation was based on both primary and secondary outcomes. For the primary outcome, our previous study indicated that 61% *(P*_*0*_*)* of people with TB in Indonesia experienced at least moderate TB-Stigma as measured by the van Rie Stigma Scale [6]. We hypothesized that our psychosocial support intervention could reduce the proportion of people with TB experiencing at least moderate TB-Stigma by a relative reduction of 30%, from 61% (*at baseline*) [6] to 42.7% (*at the sixth month after TB treatment initiation*). For the secondary outcome, we hypothesized that the support could achieve a relative reduction of 50% in the proportion of people with TB reporting symptoms consistent with depression, from 41.5% *(at baseline)* [6] to 20.75% *(at the sixth month).* Using an alpha of 5%, power of 80%, and accounting for a 15% attrition rate, we determined that a minimum of 66 and 76 participants with TB were needed to detect the anticipated effect sizes on TB-Stigma and depression, respectively.

### Sampling methods

We recruited people with DS-TB in *Puskesmas* and DR-TB in two hospitals. In each participating healthcare facility, healthcare staff supporting this study identified eligible people with DS- or DR-TB who were recently diagnosed or underwent a diagnostic procedure. Once the potential participant was bacteriologically-confirmed with TB or rifampicin-resistant TB by molecular WHO-recommended rapid diagnostic test and notified to the NTP register, we invited them to join the study. Research team members provided the potential participants with the participant information sheet and discussed its aims, objectives, methods, and outcome measures. Participants who did not consent to participate did not continue in the study.

### Psychosocial support

In this study, the psychosocial support intervention previously co-developed with key multisectoral stakeholders was comprised of four “active ingredients” [12] including individual psychological assessment and counselling, monthly peer-led group counselling, online group messaging, and community-based “TB Talks” (see Box 1). Based on our own novel conceptual frameworks, which include pathways to impact, developed through comprehensive scoping reviews [5, 13], individual and group counselling are proposed to improve participants’ knowledge and attitudes toward the disease, as well as foster a sense of psychological and social support, which in turn strengthens self-efficacy, reduces enacted stigma, and ultimately diminishes self- or internalised TB-Stigma and anticipated TB-Stigma. Complementarily, community TB Talks create dialogue and a safe space where individuals feel seen and heard, promoting greater community understanding of TB and contributing to a reduction in enacted TB-Stigma.

1 Individual psychological assessment and counselling

Following consent, we conducted baseline assessment of TB-Stigma, depression symptoms, and QoL using a locally-validated survey (see below). Immediately after the survey, we conducted the first active ingredient of our psychosocial support package, which was individual counselling provided by the study team. The counselling focused on exploring what the participants felt before and after diagnosis, what were their main ideas, concerns, and expectations, and optimal strategies to cope with their illness and treatment. Although the importance of adherence to treatment was covered within the counselling, it must be noted that the individual psychological counselling conducted during this study was much broader, and should not be confused with, standard adherence counselling delivered by National TB Programmes in Indonesia and globally. Standard adherence counselling commonly consists of provision of information and education and does not routinely include a psychological support component.

2 Monthly peer-led group counselling

After completing individual counselling, participants were invited to join the second active ingredient of the support package: monthly peer-led group counselling sessions. The sessions were held monthly, were in person, and lasted on average 60 minutes [range 45–90 minutes] depending on the group dynamics. Each session was led primarily by a peer, usually a TB survivor but sometimes, when a TB survivor was not available, a community volunteer. In this study, we recruited nine TB survivors and two community volunteers; all peers were recruited through TB Survivor civil-society organizations: *Perhimpunan Orang dengan Tuberkulosis* (POP TB) at the national level, *Terus Berjuang* (Terjang) in Depok, and *Pusako* in Padang. Collectively, for the purposes of the TB-CAPS project and this intervention, the TB survivors and community volunteers were termed “peer supporters”. It was expected that people with either DS- or DR-TB would attend two group counselling sessions during their initiation phase and could attend six in total during the intervention period. The limited study budget and grant period precluded our being able to intervene or follow-up for longer durations. Moreover, from our related research, we have demonstrated that levels of TB-Stigma and depression are highest in the treatment initiation phase rather than continuation phase, and so also assumed that psychological support would be most beneficial in the first two months and, therefore, follow-up to six months would be sufficient. Return transportation costs and refreshments and a meal for people joining the group counselling sessions were all covered by the TB-CAPS project.

Based on stakeholder feedback from our formative co-development research on the importance of being able to address questions related to TB medications and their side effects, which may not be able to be comprehensively answered by peer supporters, physicians also co-led the sessions. Physicians involved in the counselling were purposively selected. They constituted general physicians working at selected *Puskesmas* in Padang and general physicians undergoing family physician specialist training in Depok. Prior to the study implementation, all facilitators (TB survivors, community volunteers, and physicians) received a two-day, face-to-face training led by the study team members on effective communication, active listening, providing supportive responses, and leading group counselling sessions. At least one team member observed the peer-led group counselling sessions and conducted debriefings with peer supporters and physicians afterward to evaluate the sessions, identify areas for refinement in subsequent sessions, and discuss topics for upcoming meetings. All facilitators received refresher training six months after the initial training to reinforce, evaluate, and further improve their skills.

3 Online group messaging

As a complement to the group counselling, we invited all participants to join an online messaging group. The group was administered by researchers (MA, MH, FRH, DFAH, IA, ERF) and included study participants who agreed to join and peer supporters. This messaging group was intended to allow participants to share their feelings, concerns, and facilitate questions and answers between participants and peer supporters. It was also considered an important active ingredient to reach younger people with TB participating in the research, for whom such groups are regularly used communication tools. Researchers did not intervene in the dynamics of the messaging group unless it was needed to correct misconceptions relating to TB.

4 Community-based “TB Talks”

The final active ingredient of our intervention was public education delivered through “TB talks” in the geographical catchment area of each participating *Puskesmas,* once during the study period. We invited purposively one of the group counselling participants from each *Puskesmas* and his/her family members to the meeting as the main invited speakers. TB Talks focused on people’s feelings after being diagnosed, the challenges they and their family faced, and what they and others need from the *community* to ensure people with TB are supported. Wider community members from the area around *Puskesmas* were invited to the TB Talk. At the end of the community TB Talk, a physician (from either researcher or *Puskesmas* staff) provided further focused education, the subject of which was dependent on community asks and needs. Meetings lasted 1.5 hours on average.

### Evaluation

#### TB-Stigma, depression and QoL.

We evaluated the intervention by assessing TB-Stigma, depression, and QoL among participants at baseline, Month-2, and Month-6 after treatment initiation.

TB-Stigma was measured using a previously adapted and socio-culturally validated version of the van Rie TB-Stigma Scale in Bahasa Indonesia [14]. The scale comprised two forms: Form A (Personal Perspective), containing 11 items across three domains (disclosure, isolation, and guilt), and Form B (Community Perspective), containing 10 items across two domains (isolation and distancing). TB-Stigma scores were calculated using a well-recognised formula from the validated tool: (sum of item scores x 50) / (3 x number of item). Higher scores indicated greater TB-Stigma. There are no specific TB-Stigma severity categories, but based on learning from our previous studies including in Indonesia, we divided the scores into three categories: no stigma (TB-Stigma score = 0), low (≤16.67), moderate (16.68–33.33), and high (>33.33) [6]. For the statistical analyses, these four categories were reduced to two groups: no-to-low TB-Stigma (0 - 16.67) and moderate-to-high TB-Stigma (16.68 and above).

Depression was assessed using the Indonesian-validated Patient Health Questionnaire-9 (PHQ-9) [15]. Higher scores reflect more severe depressive symptoms and were divided into well-recognised categories of severity: no depression (score of 0–4), mild (5 –9), moderate (10 –14), moderately severe (15 –19), and severe (20 –27) depression. In line with the power calculations above, we further aggregated the categories into two groups: no depression to mild depression (0-9) and moderate to severe (10 and above) depression.

QoL was measured using the Indonesian version of the EQ-5D-5L, which evaluates five dimensions of health status; index scores were derived using Indonesian value sets, with higher values representing better health-related QoL [16].

### National participatory workshop

To facilitate study findings being translated into actionable activities and potentially policy and practice change, we organized a multi-stakeholder workshop at the end of the study, on May 16–17, 2025. The invited stakeholders included all participants from the first national consultation conducted during the co-development phase of the intervention (see S1 File). In addition, we invited peer supporters and study participants with TB from participating Depok and Padang study sites to ensure that community voices were meaningfully represented. The participating people with TB included TB survivors—those who had recently completed treatment—and individuals currently undergoing DS-TB or DR-TB treatment but who had sputum TB culture conversion to negative.

The workshop began with a presentation of the study findings, where TW, AF, and MAN outlined the study objectives, implementation process, and key results. To strengthen cross-learning, representatives from Mentari TB Muhammadiyah, POP TB, and Penabulu–STPI consortium—civil-society and related organizations with prior experience implementing peer-led interventions for MDR-TB—were invited to share the lessons learned from their field experience. This exchange provided policy actors and practitioners with comparative perspectives to assess the relevance, scalability, and complementarity of the TB-CAPS intervention.

Qualitative insights were gathered through open discussion fora during the workshop. We then divided participants into several small groups to collaboratively develop policy recommendations and design options for a -scaled,community-based psychosocial support model tailored to the Indonesian national TB programme context. The workshop served as a platform for evidence co-creation, policy dialogue, and consensus-building toward sustainable integration of psychosocial support into TB care.

### Acceptability, appropriateness and feasibility

Quantitative feedback data were collected from workshop participants across three domains using the Acceptability of Intervention Measure (AIM), Intervention Appropriateness Measure (IAM), and Feasibility of Intervention Measure (FIM) questionnaires, [17] each consisting of four Likert-scale items which were translated and validated in Bahasa Indonesia. The survey was conducted during the national participatory workshop.

These tools were also administered separately to participants receiving the intervention approximately 12–15 weeks after they completed the intervention via online surveys. The online survey was disseminated between April 28 to May 15, 2025.

In addition, we estimated the costs associated with delivering TB-CAPS’ “active ingredients”. These costs included transportation and food for study participants and peer supporters, as well as expenses related to coordination, training, and study permits.

### Data analysis

We conducted both descriptive and inferential analyses to assess changes in TB-Stigma, depression, and QoL over time. Descriptive statistics were used to summarize participants’ TB-Stigma, PHQ-9 scores, and QoL scores at baseline, Month-2, and Month-6. To evaluate changes in individual TB-Stigma items, we applied McNemar and Cochran’s Q tests to determine differences between baseline and Month-2 and across the three time points (baseline *vs.* Month-2 *vs.* Month-6). We also applied complete-case approach, restricting the analyses to completers at each measurement timepoint (Month-2 and Month-6).

To evaluate the acceptability, appropriateness, and feasibility of the intervention ingredients, we applied descriptive analyses on quantitative feedback collected from both study participants and multi-stakeholder workshop participants. Each measure (AIM, IAM, and FIM) was summarized using numbers (n), percentages (%), and 95% confidence intervals where appropriate. In parallel, qualitative feedback was gathered from participants during the workshop and systematically analysed to provide additional insights into the quantitative findings.

#### Human Ethics and consent to participate.

All participants received the Participant Information Sheet (PIS) and gave their informed consent to join the TB-CAPS intervention activities (individual counselling, group counselling, and online group messaging) and all assessments, in accordance with the Declaration of Helsinki. All participants of the national participatory workshop also received the Participant Information Sheet (PIS) and gave their informed consent to join prior to the workshop. This study received research ethical approval from the Research Ethical Committee of Liverpool School of Tropical Medicine (RGETEM044) and the Faculty of Medicine Universitas Indonesia (KET-1169/UN2.F1/ETIK/PPM.00.02/2023). Specifically, all participants gave consent for publication.

## Results

During the study period, 136 eligible individuals were diagnosed with TB in the study sites, 85 in Depok and 51 in Padang. In Depok, all diagnosed individuals consented to participate in the baseline assessment and individual counselling, whereas in Padang, 44 individuals (86%) agreed to take part in the baseline assessment with field notes indicating that the predominating reasons for non-participation related to time constraints and distance of domicile from healthcare facilities. This left 129/136 (95%) participants with data available to analyse.

### TB-Stigma, depression, and quality of life at baseline

The median age of the cohort was 43 (IQR 27.5-54.0), with 15–29 years (29%) being the largest age group. The majority of participants were male (57%, 95%CI: 49–67%) (Table 1). Most participants were married (64%, 57–73%), had completed secondary education (54%, 46–63%) and had income-earning jobs (57%, 49–64%).

**Table 1:** Participant characteristics (N = 129).

Characteristics	Depok, N = 85			Padang, N = 44			Total, N = 129		
	*n*	*%*	*95%CI*	*n*	*%*	*95%CI*	*n*	*%*	*95%CI*
Gender									
Male	48	56	(46-67)	26	59	(43-74)	74	57	(49-67)
Female	37	44	(33-54)	18	41	(26-57)	55	43	(33-51)
Age, median (IQR), *years*	43	(27-58)	(36–48)	42	(28-52)	(37–48)	43	27-54	(38–47)
Age Group									
15–29 years	26	31	(21-41)	12	27	(14-41)	38	29	(22–38)
30–39 years	11	13	(7–21)	6	14	(5-25)	17	13	(8–19)
40–49 years	18	21	(13-30)	11	25	(13-39)	29	22	(16–30)
50–59 years	16	19	(11–27)	12	27	(15-41)	28	22	(15–29)
60 years and above	14	16	(9–25)	3	7	(0-15)	17	13	(8–19)
Marital status									
Married	53	62	(52-72)	30	68	(53-82)	83	64	(57-73)
Not married	25	29	(20-39)	13	30	(16-44)	38	29	(22–37)
Widower	7	8	(3–15)	1	2	(0-8)	8	6	(2–11)
Education level									
No school	10	12	(5–19)	0	0	(0-0)	10	8	(4–12)
Primary level	19	22	(14-32)	13	30	(16-44)	32	25	(18 –33)
Secondary level	45	53	(42-64)	25	57	(41-72)	70	54	(46-63)
College/University	11	13	(6–20)	6	14	(5-24)	17	13	(8–19)
Employment									
Employed	44	52	(41-62)	29	66	(51-80)	73	57	(49-64)
Unemployed	41	48	(38-59)	15	34	(20-49)	56	43	(36–51)
Type of TB									
DS-TB, new	52	61	(50-72)	28	64	(49-78)	80	62	(53-70)
DS-TB, recurrent	12	14	(7 –22)	7	16	(6-28)	19	15	(9–21)
DR-, MDR-TB	21	25	(16-34)	9	20	(9-33)	30	23	(16–32)

There were distinct patterns of TB-Stigma across the domains of disclosure, isolation, and guilt. In general, fewer participants reported concerns related to the domain of isolation than the domains of TB diagnosis disclosure or guilt related to TB diagnosis. The most prevalent concern in the disclosure domain was about being careful whom to tell about TB diagnosis (64%, 95%CI 57–73% in total; 66%, 55–77% in Depok; 61%, 48–75% in Padang) and fear of telling people outside their family about having TB (43%, 35–52%; 46%, 35–58%; 39%, 25–52%, Table 2). For the guilt domain, participants feelings of guilt due to a perception that their family had a burden of caring for them was the most prominent concern (43%, 35–51%; 48%, 38–59%; 32%, 19–46%), followed by guilt related to lifestyle behaviours such as smoking or drinking (36%, 27–44%; 38%, 28–48% and 32%, 18–47%).

**Table 2:** TB-Stigma at baseline (TB diagnosis) in Depok and Padang (N = 129).

Domain of TB-Stigma	Depok, N = 85			Padang, N = 44			Total, N = 129		
	n	%	(95%CI)	n	%	(95%CI)	n	%	(95%CI)
*Domain: Disclosure*									
P5. I am afraid to tell people outside my family that I have TB	39	46%	(35-58%)	17	39%	(25-52%)	56	43%	(35-52%)
P6. I am afraid to tell others that I have TB because others may think that I also have HIV/AIDS	17	20%	(12-29%)	10	23%	(11-35%)	27	21%	(14-28%)
P8. I choose carefully who I tell about having TB	56	66%	(56-77%)	27	61%	(48-75%)	83	64%	(55-73%)
P11. I am afraid of other people to tell my family that I have TB	21	25%	(16-34%)	9	20%	(10-33%)	30	23%	(16-31%)
*Domain: Isolation*									
P1. I feel hurt by how others react to knowing that I have TB	13	15%	(8-23%)	14	32%	(18-46%)	27	21%	(14-28%)
P2. I have lost friends when I shared with them that I have TB	10	12%	(5-19%)	6	14%	(5-24%)	16	12%	(7-19%)
P3. I feel lonely	21	25%	(16-34%)	9	20%	(9-33%)	30	23%	(16-31%)
P4. I am afraid of going to TB clinics because other people may see me there	17	20%	(12-28%)	6	14%	(4-26%)	23	18%	(12-25%)
*Domain: Guilt*									
P7. I feel guilty because my family has the burden of caring for me	41	48%	(38-59%)	14	32%	(19-46%)	55	43%	(35-51%)
P9. I feel guilty for getting TB because of my smoking, drinking, or other lifestyle behaviours	32	38%	(28-48%)	14	32%	(18-47%)	46	36%	(27-44%)
P10. I am worried about having HIV/AIDS	19	22%	(14-31%)	11	25%	(13-38%)	30	23%	(16-31%)

### Changes of TB-Stigma, depression, and QoL

**Fig 1:**
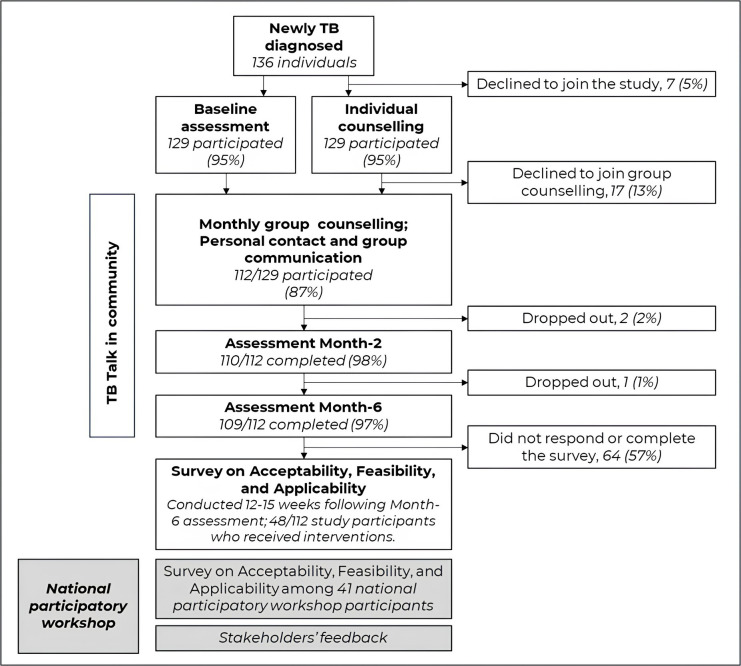
Participant flow.

Of 129 participants at baseline assessment, 17 declined to join the group counselling sessions with field notes including this was due to other commitments (n = 15) or feeling shy (n = 2). This left 112 out of 129 participants joining group counselling (Total: 87% [81–92%]; Depok: 75 [88%, 81–95%]; and Padang: 37 [84%, 72–94%]). Of 112 participants, 41(43%) of them responded to the online questionnaire delivered 12–15 weeks after the end of interventions (Fig 1).

Among 112 participants, 75 (67%, 59–76%) had moderate TB-Stigma and 2 (2%, 0–5%) had severe TB-Stigma (Table 3). The proportion of those with at least moderate TB-Stigma reduced significantly from 77% (69–85%) to 55% (45–66%) at Month-2 (p = 0.002) and 28% (20–37%) at Month-6 (p < 0.001). At Month-2, three TB-Stigma items showed a statistically significant reduction: fear of disclosing TB status to others, fear of being seen visiting clinics due to TB, and guilt about burdening one’s family due to illness. By Month-6, a greater number of TB-Stigma items, particularly those in Disclosure and Guilt, showed progressive decline compared to baseline.

**Table 3:** Change of TB-Stigma at baseline (diagnosis), Month-2, to Month-6.

Domain of TB-Stigma	Baseline, N = 112			Month-2, N = 100			Month-6, N = 99			P*	P**
	*n*	*%*	*95%CI*	*n*	*%*	*95%CI*	*n*	*%*	*95%CI*		
TB-Stigma											
No stigma	1	1	(0-3)	5	5	(1–9)	7	7	(2–13)	0.002^^^	<0.001^^^
Low	34	30	(21-39)	47	47	(38-56)	71	72	(63-81)		
Moderate	75	67	(59-76)	47	47	(37-57)	21	21	(13-30)		
High	2	*2*	(0-5)	1	1	(0-3)	0	0	(0-0)		
TB-Stigma score, *median, 95% CI (IQR)*	20	18-21	(17–24)	17	15-18	(11–21)	8	6-11	(5–17)	<0.001	<0.001
*Domain: Disclosure*											
P5. I am afraid to tell people outside my family that I have TB	52	46	(37-56%)	35	35	(26-44%)	27	27	(18-36%)	0.031	0.004
P6. I am afraid to tell others that I have TB because others may think that I also have HIV/AIDS	25	22	(15-30%)	22	22	(15-30%)	8	8	(3-14%)	1.000	0.006
P8. I choose carefully who I tell about having TB	72	64	(55-72%)	52	52	(42-62%)	48	49	(38-59%)	0.059	0.030
P11. I am afraid of other people to tell my family that I have TB	27	24	(16-32%)	22	22	(14-30%)	16	16	(9-23%)	0.541	0.209
*Domain: Isolation*											
P1. I feel hurt by how others react to knowing that I have TB	24	21	(14-29%)	16	16	(10-24%)	8	8	(3-14%)	0.791	0.045
P2. I have lost friends when I shared with them that I have TB	14	13	(6-19%)	7	7	(3-13%)	5	5	(1-10%)	0.453	0.282
P3. I feel lonely	26	23	(16-31%)	12	12	(6-20%)	12	12	(6-19%)	0.077	0.102
P4. I am afraid of going to TB clinics because other people may see me there	18	16	(10-23%)	7	7	(2-13%)	3	3	(0-7%)	0.039	0.007
*Domain: Guilty*											
P7. I feel guilty because my family has the burden of caring for me	50	45	(36-54%)	31	31	(23-40%)	19	19	(12-27%)	0.021	<0.001
P9. I feel guilty for getting TB because of my smoking, drinking, or other lifestyle behaviours	41	37	(28-46%)	27	27	(18-37%)	19	19	(12-27%)	0.064	0.006
P10. I am worried about having HIV/AIDS	27	24	(16-32%)	22	22	(15-31%)	14	14	(8-22%)	0.541	0.41

*Significance (P-values) between baseline and Month-2 assessment, tested with McNemar; **Significance (P-values) between baseline, Month-2 and Month-6 assessment, tested with Cochran’s Q test; ^Significance test by merging categories to No-Low and Moderate-High Stigma.

**Fig 2:**
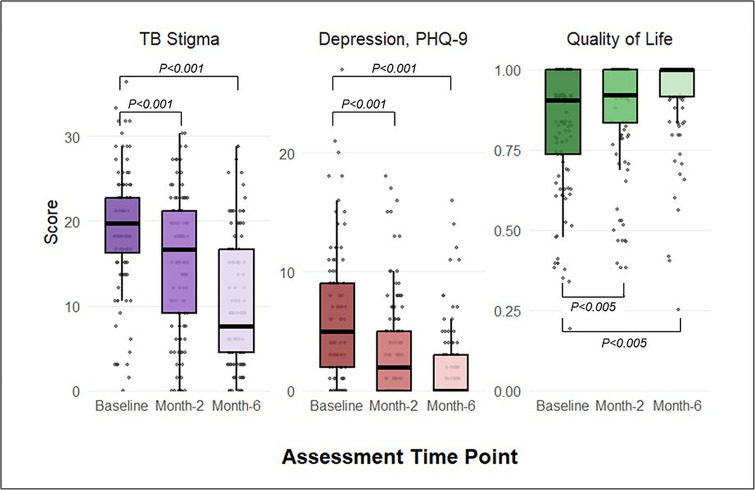
TB-Stigma, depression and quality of life at baseline, Month-2 and Month-6. *Significance (P-values) between baseline and Month-2 assessment, tested with McNemar; **Significance (P-values) between baseline, Month-2 and Month-6 assessment, tested with Cochran’s Q test. The range for TB-Stigma is 0-50, depression is 0-27, and quality of life is 0-1..

At baseline, the median (IQR) of overall TB-Stigma score was 20 (IQR:17–24). The median TB-Stigma score were similar in Depok (20, IQR:18–24) and Padang (18, IQR: 13–23). Overall, the TB-Stigma scores decreased significantly to 17 (IQR: 11–21) at Month-2 (p < 0.001) and 8 (IQR: 5–17) at Month-6 (p < 0.001, Fig 2).

Depression scores showed a similar pattern, with a significant reduction from 6 (IQR: 3–9) at baseline to 2 (IQR: 1–5) at Month-2 (p < 0.001) and 0 (IQR: 0–3) at Month-6 (p < 0.001). The decline was statistically significant at Month-2 compared to baseline. In terms of depression severity categories, the proportion of people with depression symptoms reduced significantly from 62% to 38% at Month-2 (p = 0.019) and to 14% at Month-6 (p < 0.001).

For QoL, an improvement was observed from 0.91 (IQR:0.63-1.00) at baseline to 0.92 (IQR:0.83-1.00, p < 0.005) at Month-2 and 1.00 (0.91-1.00, p < 0.005) at Month-6, but not between Month-2 and Month-6 (p = 0.126).

The proportion of participants with at least moderate TB-Stigma was higher in Depok (82%, 95%CI:77–93%) than in Padang (60%, 43–74%) at baseline (Table 4). The proportion decreased significantly in Depok at Month-2 (49%, 95% CI:38–61%) and Month-6 (26%, 95% CI:15–37%). In Padang, there was no statistically significant reduction at Month-2 (67%, 95% CI: 50–82%), but a significant reduction was observed at Month-6, compared to baseline and Month-2 (33%, 95% CI: 17–50%).

**Table 4:** TB-Stigma, depression, and quality of life at baseline, Month-2, and Month- 6.

Variables	Baseline			Month-2			Month-6			P^a^	P^b^
	n	%	95%CI	n	%	95%CI	n	%	95%CI		
**Depok**			**n = 75**			**n = 67**			**n = 66**		
TB-Stigma											
No to Low	11	15	(7-23%)	34	51	(39-63%)	49	74	(63-85%)	<0.001	<0.001
Moderate to Severe	64	85	(77-93%)	33	49	(38-61%)	17	26	(15-37%)		
Depression											
No to minimal	28	37	(27-48%)	40	60	(47-71%)	57	86	(77-94%)	0.090	<0.001
Mild to moderate	41	55	(44-67%)	25	37	(26-49%)	9	14	(6-23%)		
Moderately severe to severe	6	8	(3-14%)	2	3	(0-8%)	0	0	(0-3%)		
**Padang**			**n = 37**			**n = 33**			**n = 33**		
TB-Stigma											
No to Low	15	41	(26-57%)	11	33	(18-50%)	22	67	(50-83%)	0.343	0.011
Moderate to Severe	22	60	(43-74%)	22	67	(50-82%)	11	33	(17-50%)		
Depression											
No to minimal	15	41	(24-56%)	22	67	(50-82%)	28	85	(71-96%)	0.097	<0.003
Mild to moderate	16	43	(27-60%)	8	24	(11-39%)	4	12	(3-24%)		
Moderately severe to severe	6	16	(5-29%)	3	9	(0-21%)	1	3	(0-10%)		
**Total**			**n = 112**			**n = 100**			**n = 99**		
TB-Stigma											
No to Low	26	23	(16-31%)	45	45	(35-55%)	71	72	(63-81%)	0.002	<0.001
Moderate to Severe	86	77	(69-84%)	55	55	(45-65%)	28	28	(19-37%)		
Depression											
No to minimal	43	38	(30-46%)	62	62	(52-71%)	85	86	(79-93%)	0.019	<0.001
Mild to moderate	57	51	(41-61%)	33	33	(24-42%)	13	13	(7-20%)		
Moderately severe to severe	12	11	(5-17%)	5	5	(1-9%)	1	1	(0-3%)		

^*a*^*Significance (P-values) between baseline and Month-2 assessment, using McNemar tests;* ^*b*^*Significance (P-values) between baseline, Month-2 and Month-6 assessment, using Cochran’s Q tests.*

Mild to moderate depressive symptoms among participants were 55% (95% CI: 44–67%) in Depok and 43% (95% CI: 27–60%) in Padang. Six participants in Depok and six in Padang exhibited moderately severe to severe depressive symptoms. Two participants in Depok reported having suicidal thoughts after being diagnosed with TB, both of whom were promptly supported by the study team to seek and be linked to mental health care services. In both cities, there was no reduction in depression at Month-2; however, the proportion of participants with at least mild depression decreased significantly at Month-6.

### Acceptability, appropriateness, and feasibility

**Fig 3:**
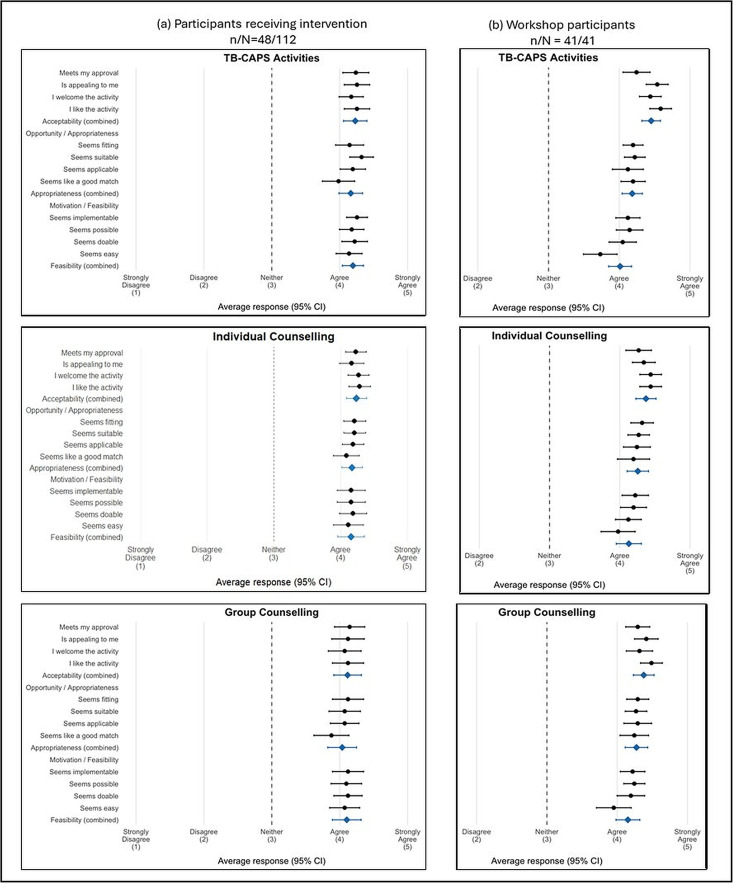
Acceptability, appropriateness, and feasibility of overall TB-CAPS “active ingredients”, individual counselling, and peer group counselling evaluated by (a) study participants (n=48) and (b) workshop participants (n=41). Point estimates and confidence intervals in black are specific elements of acceptability, feasibility, and appropriateness. Point estimates and confidence intervals in blue combine the scores of the specific elements of acceptability, feasibility, and appropriateness.

We collected 41/41 (100%) responses from workshop participants and 48/112 (43%) responses from study participants receiving the intervention regarding the acceptability, appropriateness, and feasibility of the psychosocial intervention. Overall, most responses were positive, with high scores of acceptability, appropriateness, and feasibility (Fig 3).

However, several important issues were identified through the workshop dialogue. A key concern raised was the capacity of *Puskesmas* to implement and replicate the intervention at primary care level. There was a perceived doubt about whether healthcare workers can implement this additional intervention program beyond their current daily tasks. During the dialogue, the need to develop or strengthen networks and partnerships between Puskesmas and community and civil-society organizations, empower peer supporters, and seek help from volunteers in implementing the activities were all emphasized. Participants also suggested integrating the intervention with other adjunctive TB support services to ensure alignment and adjusting the timing of group counselling sessions to allow more people, particularly those working during weekdays, to participate. Methodological suggestions to strengthen the evidence-base for the effectiveness of the intervention included introducing a control group and doing a cluster- or individually-randomised controlled trial, expanding implementation beyond Padang and Depok prior to national scale-up, and integrating referral pathways to existing mental health services to strengthen continuity of care.

For online group messaging, interactions were largely limited to disseminating information about TB and schedules for group counselling sessions. Only a few participants were active in the group, and most preferred to contact peer supporters privately when they needed to ask questions or seek advice.

Of the five TB Talks conducted during the study period, most attendees were female, primarily those living close to the event locations and those actively involved in integrated health posts (*Posyandu*), which are community-based health services with a focus on providing maternal and child health care.

### Costs of implementing the psychosocial support intervention activities

We estimated the costs of implementing the individual and group counselling sessions. Total costs of psychosocial support were IDR 423,328–450,282 (US$26.7-28.4) per person with TB (US$1 = 15,855; World Bank, 2024) [18]. The TB-Stigma and mental health assessments, along with individual counselling—which were often conducted together—cost between IDR 178,000 and 205,000 (US$11.2–12.9, per person per session. Group counselling sessions cost IDR 246,500 (US$15.5) per person per session.

Reflecting on these costs during the workshop, participants perceived them as reasonable to budget for in NTP activities and integrate into routine practice, although they noted that lower costs would further enhance acceptability and replicability.

## Discussion

This prospective single-arm interventional cohort study demonstrated that the TB-CAPS psychosocial support intervention, which included individual counselling, group counselling, online group messaging, and educational TB Talks, has the potential to reduce TB-Stigma and depressive symptoms, as well as improve QoL among people with TB. Significant reductions in TB-Stigma and depression scores were observed at both Month-2 and Month-6, indicating sustained psychosocial benefits during the six-month treatment period. The intervention was also perceived as acceptable, appropriate, and feasible by participants and stakeholders. However, several implementation challenges were identified, including the limited capacity of public primary healthcare facilities, the timing and scheduling of peer group meetings, and the need for better integration with existing government-provided TB and mental health services.

The baseline TB-Stigma scores observed in Depok (median 20, IQR:18–24) and Padang (18, IQR: 13–23) were similar to those reported in our previous study conducted across seven provinces in Indonesia [6]. Participants in Depok, a more urban setting than Padang, expressed a slightly higher level of anticipated stigma, reflecting persistent fears of disclosure and social judgment. Higher TB-Stigma in urban *vs.* rural areas has been reported elsewhere [19–21], often linked not to knowledge or education levels but to misconceptions about TB and social proximity within densely populated neighbourhoods, where individuals may fear being recognized as having TB. In contrast, a study from South Africa found that anticipated, internalized, and enacted stigma were higher in rural settings compared to peri-urban areas [21]. Our previous study also showed no relationship between urban-rural settings and TB-Stigma. This contrast suggests that stigma patterns vary across contexts and may reflect differences in cultural norms, community cohesion, social capital, urbanization, and ethnic backgrounds.

This study highlights a prevalent concern among people with TB: being careful about whom to tell about TB diagnosis and the fear of disclosing the disease to people outside one’s family, indicating the persistence of anticipated TB-Stigma. In addition, self- or internalised TB-Stigma was prominent at baseline, as reflected by the high proportion of people who felt guilty for burdening their families with care responsibilities and for perceived lifestyle-related causes of their illness, even when these were not objectively proven or linked. We consider that the psychosocial support provided during TB-CAPS through individual and peer group counselling may have contributed to greater awareness, emotional resilience, and confidence among participants, enabling them to better cope with self-stigmatisation and stress from TB-Stigma [22].

The observed reduction in TB-Stigma scores by Month-2 shows the potential and prompt effectiveness of this intervention. In our previous study in seven provinces of Indonesia [6], there was no difference in TB-Stigma scores between participants surveyed in the intensive phase (corresponding to the baseline assessment in this study) *vs.* early continuation phase (corresponding to the Month-2 assessment in this study) of treatment. The reduction in TB-Stigma observed here may also parallel clinical improvement and symptom relief, which could help alleviate fears of being identified as infectious. Over time, participation in group meetings may enhance understanding of TB and improve self-efficacy, thereby reducing both misconceptions about and fear of recognition of TB diagnosis. Together, these findings underscore the importance of integrating psychosocial support into routine TB care.

As described in our previous scoping review on community-based interventions to reduce the psychosocial impact of infectious diseases [13], this study highlights that both individual and group counselling can contribute to people with TB feeling psychologically and socially supported. Combined with increased knowledge about TB, these interventions can enhance self-efficacy and confidence while reducing self-stigma—which, if left unaddressed, may increase the risk of depression, anxiety, and other mental health problems.

There is limited evidence on the effectiveness of peer-led group counselling in reducing TB-Stigma and depression. Most existing interventions, such as cash transfers and peer counselling, have primarily focused on improving treatment adherence and clinical outcomes [23–25]. More robust evidence is available from the HIV/AIDS and leprosy fields [26–28]. Group counselling sessions led by peers or affected communities, conducted regularly, have been shown to empower individuals with HIV/AIDS by facilitating acceptance of their diagnosis and improving treatment adherence [26, 27]. In Indonesia, a study also found that multi-session individual counselling reduced stigma and depression among people affected by leprosy by enhancing their confidence [28].

In this study, depressive symptoms decreased significantly across time points. In our previous study in Indonesia [6], there was no difference in depression scores between participants surveyed in the intensive phase *vs.* early continuation phase (corresponding to the baseline and the Month-2 assessment in this study, respectively) of treatment. In this current TB-CAPS study, at baseline, several participants reported emotional distress, with two individuals expressing suicidal ideation soon after being diagnosed with TB. Many others described social withdrawal, distancing themselves from friends, neighbours, and community activities due to feelings of shame or fear of discrimination. These findings reflect the close interconnection between TB and mental health, as has been consistently demonstrated in previous research [6, 29, 30]. Depression often arises as a psychological consequence of TB-Stigma, particularly when individuals internalize negative societal attitudes or experience social rejection. Moreover, TB illness, TB-Stigma, and depression frequently interact as part of a syndemic triad, mutually reinforcing one another and collectively exerting a detrimental impact on QoL, treatment adherence, and functional well-being [31, 32]—both during and after the course of TB illness.

Given these intersections, it is crucial to routinely assess TB-Stigma, depression, and QoL, especially at the time of diagnosis. Early identification of psychological distress allows healthcare providers to deliver timely and targeted psychosocial support both in healthcare facilities and, vitally, in communities, such as Integrated Health Posts (*Posyandu*) in Indonesia. Awareness and sensitivity among healthcare workers are especially important to recognize these challenges, facilitate appropriate referrals to counselling or mental health services, and promote empathic patient–provider interactions [33]. In addition, for future implementation, it is important to select assessment tools that are appropriate to the current health system context—tools that are brief yet sufficiently sensitive to detect TB-Stigma and mental health problems; to define who should do such assessment; and characterise when the assessment should be conducted (at the diagnosis, start of treatment, end of the initial phase, end of treatment, or all time points).

The co-developed psychosocial TB-CAPS intervention was widely perceived as acceptable, appropriate, and feasible by participants and stakeholders. The drop-out rate of group counselling was also low at both settings (9/75 = 12% in Depok and 4/37 = 11% in Padang), providing a practical indication that the group counselling was acceptable for those joining the counselling. Nonetheless, several implementation challenges emerged, including the limited capacity of public primary care facilities, the scheduling of peer group meetings to convenient times for participants especially with respect to work hours, and the need for stronger integration with other existing TB and mental health services.

The costs of the intervention were reasonable. The cost per person to attend two required months of group counselling during the intensive phase of treatment was US$31, representing 12.2% of the overall cost of treating drug-susceptible TB (US$254) [6]. These costs could likely be further reduced when the intervention is implemented at scale and in non-research settings. Given the effect of the intervention observed by Month-2 of follow-up, delivering group counselling during the intensive treatment phase only, when it is likely people with TB need it most, would be most feasible and cost efficient for better clinical and psychosocial outcomes [34, 35].

The findings from our study suggest the potential for scale-up, although several issues need to be addressed to ensure the intervention is actionable and implementable. As highlighted in the participatory workshop, implementation should be integrated within existing systems to avoid siloing and fragmentation across specialties, particularly by linking with mental health services and established referral pathways. Training is required for TB-related healthcare workers at the primary care level, peer supporters, and general physicians. In addition, collaboration with TB survivor organizations and other relevant civil society organizations, should be strengthened. These organizations could receive training of trainers to enable them to train peer supporters, provide supervision, and help minimize the workload of primary care healthcare workers. Scheduling should remain flexible, with peer supporters identifying the most appropriate times for group counselling (e.g., weekends in urban settings, and after working hours on weekdays in rural areas). Another important issue is financing these activities; funding may be sourced not only from within the National TB Programme but also, perhaps as co-funding, from provincial or district health offices, with potential collaboration from philanthropic organizations.

Despite showing the potential benefits of interventions, this study has several limitations. First, as a prospective cohort study without a control group, it was not possible to establish causal inference or fully attribute the observed improvements in TB-Stigma, depression, and QoL to the TB-CAPS psychosocial intervention. However, comprehensive quantitative data from our previous cross-sectional study conducted across seven provinces in Indonesia suggest that the positive effects of this study intervention on reducing TB-Stigma and depression were greater than those observed among individuals who did not receive the intervention and further, more detailed analyses comparing data from these studies are ongoing. Future studies employing controlled or randomized designs will be important to rigorously evaluate the intervention’s effectiveness. Second, the study was conducted in Depok and Padang, two settings which may not be representative of Indonesia’s diverse epidemiological and sociocultural contexts. Third, since we applied a complete-case approach in our analysis and there were some (13/112, 12%) missing cases, particularly at Month-2, the findings may not reflect the true changes in TB-Stigma and depression amongst all participants recruited, most likely leading to underestimation of thereductions. Fourth, although all peer supporters underwent standardized and refresher training, variability in their communication skills and counselling approaches may have influenced participant engagement and outcomes. Strengthening supervision, ongoing capacity-building, and formalised remuneration or in-kind support for peer supporters will be important in future scale-up to ensure consistent quality of delivery. In addition, despite the high level of agreement with the TB-CAPS “active ingredients,” the low response rate to the online survey assessing acceptability, appropriateness, and feasibility among study participants limits the generalisability of these findings. This was likely due to the survey being administered 12–15 weeks after the intervention had ended. It is therefore critical for future studies to ask participants’ feedback during active participation or soon after the completion of their participation.

## Conclusions

The psychosocial support intervention provided in this TB-CAPS study shows potential for reducing TB-Stigma and depressive symptoms and for improving QoL. However, a controlled study with complementary health economic evaluation is needed to confirm its effectiveness and cost-effectiveness. Peer-led group counselling during the intensive treatment phase may represent a feasible priority window for further scale-up, and adequately trained and supervised peer supporters can complement Primary Health Centre services (Puskesmas in the Indonesian context) in delivering such psychosocial support. Participants and stakeholders found the intervention acceptable, appropriate, and feasible within existing TB services Scale-up and suggested that institutionalisation of integrated psychosocial support within national TB programmes would be achievable as long as there were clearly defined roles of healthcare workers and peer supporters, alongside sufficient and sustained funding.

**Box 1. Details of “active ingredients” of TB-CAPS.**

**Table:** 

Active ingredients	Delivered by	Time	Topics	Quality assurance
Individual counselling	Study team member	Once at the first visit (baseline) after the baseline assessment.	• Assessment of TB-Stigma, depression, and quality of life • Psychological counselling • Referral to mental health services if needed	Questionnaire checking; Supervision; Debriefing
Peer-led Group counselling	Peer supporters as lead; physicians as co-lead	Monthly meetings; at least during the first two months of TB treatment but up to six sessions throughout standard 6-month treatment duration. The specific time was agreed upon jointly by participants and Primary Health Centre staff.	Introduction for new participants Basic topics: - Knowledge about TB, myths, and misperceptions. - Social support - Sharing additional topics: decided together with participants	Supervision; Debriefing by team members; Refresher training for peers
Online group messaging	Moderated by study team member; attended by peer supporters; only participants who agreed to join were included.	Throughout the study period.	No specific topic. Participants may raise issues or questions through the online messaging platform.	Participation
TB Talks	In each Primary Health Centre, one study participant and his/her family member.	Once during the study period; the specific time was agreed upon by Primary Health Centre staff and community leaders.	Basic topic: - Knowledge about TB, myths, and misperceptions. - Family support - Stigma and motivation to maintain health Topics can be expanded based on needs	Attendance list; Pre- and post-test; Supervision and debriefing

## Supporting information

S1 File Participants in Delphi survey and national participatory workshop (16–17 May 2026). (DOCX)

S2 File TB-CAPS Training Material Handouts: Peer Support. Guide to Providing Psychosocial Support for Individuals with Tuberculosis (PDF in English).
(PDF)

S3 File TB-CAPS Handout Materi Pelatihan, Dukungan Teman Sebaya. Panduan Dalam Memberikan Dukungan Psikososial Bagi Pejuang TB (PDF in Bahasa Indonesia).
(PDF)
