# Supplementary material for: Community-based, peer-led psychosocial support to address stigma and reduce depression among adults with tuberculosis in Indonesia: A prospective interventional cohort study
Source: PLOS Glob Public Health. 2026 Jul 16;6(7):e0006754. doi: 10.1371/journal.pgph.0006754 (PMC13375138; doi:10.1371/journal.pgph.0006754)
Supplement: S1 File — (DOCX) [file pgph.0006754.s001.docx]

# Supplementary file

**S1 File**. Participants in Delphi survey and national participatory workshop (16-17 May 2026)

| **Stakeholders** | **Invited organisations/institutions** | **Numbers** |
| --- | --- | --- |
| TB Program officers | - - District level, Depok | 2 |
|  | - - District level, Padang | 3 |
|  | - - Public Primary Care Center, Depok | 3 |
|  | - - Public Primary Care Center, Padang | 2 |
|  | - - Hospital, Depok | 1 |
| TB-related Civil Society Organisations | - - Indonesian TB Patient Organization, *Perhimpunan Organisasi Pasien* (POP-TB) | 2 |
|  | - - Stop TB Partnership Indonesia (STPI) | 1 |
|  | - - Pejuang Tangguh (PETA) West Java | 1 |
|  | - - Terus Berjuang (Terjang) West Java | 2 |
|  | - - PUSAKO, West Sumatera | 1 |
|  | - - Penabulu-STPI Consortium | 2 |
|  | - - Mentari TB Muhammadiyah | 1 |
|  | - - Lembaga Kesehatan NU (LKNU) | 2 |
|  | - - Yayasan KNCV Indonesia | 1 |
|  | - - Indonesian Tuberculosis Elimination Organization, *Perkumpulan Pemberantasan Tuberkulosis Indonesia* (PPTI) | 1 |
| Researchers and Research Institutions/Bodies | - - Indonesian Expert Committee for TB (Komli-TB) | 1 |
|  | - - Indonesian TB Research Network (JetSet-TB) | 1 |
|  | - - Indonesian Psychiatrist Association (PDSKJI) | 1 |
|  | - - Community Medicine, Faculty of Medicine, Universitas Indonesia | 1 |
|  | - - Gadjah Mada Centre for Tropical Medicine | 1 |
|  | - - The Indonesian Respiratory Programmatic Implementation and Research Institute (RPRI) | 2 |
| Study participants | - - Study participants, Depok | 2 |
|  | - - Study participants, Padang | 2 |
| Peer supporters | - - Peer supporters, Depok | 3 |
|  | - - Peer supporters, Padang | 2 |
| **Total** |  | **41** |
