# Supplementary material for: Community-based, peer-led psychosocial support to address stigma and reduce depression among adults with tuberculosis in Indonesia: A prospective interventional cohort study
Source: PLOS Glob Public Health. 2026 Jul 16;6(7):e0006754. doi: 10.1371/journal.pgph.0006754 (PMC13375138; doi:10.1371/journal.pgph.0006754)
Supplement: S2 File — Guide to Providing Psychosocial Support for Individuals with Tuberculosis (PDF in English). (PDF) [file pgph.0006754.s002.pdf]

TB CAPS

Training Material Handouts

Peer Support

Guide to Providing Psychosocial Support

for Individuals with Tuberculosis

Fakultas Kedokteran Universitas Indonesia, Indonesia

Primary Health Care Center Cluster IMERI FKUI, Indonesia

Liverpool School of Tropical Medicine, Liverpool, UK

2024

INTRODUCTION

We are pleased to introduce this Peer Support module. In a dynamic and evolving landscape of health and wellness, peer support has become not just valuable, but essential. This module serves as a guide to meaningful relationships, deeper understanding, and empowerment.

On the journey toward holistic health, we often find that the most meaningful support comes from those who have walked a similar path. Peer support moves beyond traditional frameworks, drawing on the power inherent in community and the potential for positive change through shared experience.

This module is more than a collection of lessons — it is an invitation to explore the depth and richness of peer support across a range of contexts. Whether you are a health professional, a peer support worker, or someone seeking support yourself, the pages ahead offer insights meant to inform, inspire, and guide.

As we explore the essence of peer support, recurring themes emerge: empathy, resilience, and the remarkable capacity of individuals to uplift one another. Through practical tools, real-life stories, and evidence-based strategies, this module seeks to harness the true potential of peer support. By embracing its principles, we not only strengthen our own well-being but also help build a more compassionate and supportive community.

The drafting team wishes to extend its deepest gratitude to all who contributed their expertise, insight, and enthusiasm to bring this module to life. We hope it serves as a source of inspiration, a catalyst for positive change, and a reminder that, together, we can build a world where peer support is not merely a concept — but a lived reality.

Authors

Contents

1. TB, STIGMA, AND PSYCHOSOCIAL IMPACT OF TUBERCULOSIS.. 5

2. THE CONCEPT OF PSYCHOSOCIAL SUPPORT AND PEER SUPPORTS.. 8

3. ACTIVE LISTENING, EFFECTIVE LISTENING, AND COMMUNICATION SKILLS 12

4. USING CLOSED AND OPEN QUESTIONS 21

5. ROLES AND LIMITATIONS, CHALLENGING SITUATIONS, AND CONFIDENTIALITY 25

6. MOTIVATING SKILLS 28

7. RECOGNIZING AND RESPONDING TO DISTRESS, INCLUDING REFERRAL TO A MENTAL HEALTH PROFESSIONAL FOR FURTHER SUPPORT 30

8. SKILLS FOR GUIDING SUPPORT GROUP 33

REFERENCES 38

1. TB, STIGMA, AND PSYCHOSOCIAL IMPACT OF TUBERCULOSIS

Tuberculosis, Stigma, and Peer Support

Tuberculosis (TB) is an infectious disease that accounts for 10 million new cases every year and causes one million deaths every year in the world. TB disease in Indonesia is still very high, and Indonesia is the country with the second highest number of TB cases in the world. The TB problem is not only related to the availability of vaccines and medicines, but is also related to social problems that often arise when someone experiences TB. One of these social problems is stigma.

Tuberculosis and Stigma

The term 'stigma' refers to "exclusion, rejection, blame, or devaluation of a person arising from experiences or concerns about adverse social views". Likewise, stigma refers to a complex and inherent social process when someone experiences certain diseases, including tuberculosis (TB). For example, people who have TB can sometimes be wrongly considered to be cursed, dirty, or needing to be ostracized and kept away from society. The association of TB with certain judgments such as this results in individuals with TB being judged unfairly and they are often characterized by qualities and behavior that are undesirable to the general public.

Historically, TB has been stigmatized due to its contagious nature and a lack of knowledge among the general public about its causes, modes of transmission, or treatment. This stigma is also often associated with disadvantaged groups, for example those who live below the poverty line, who are incarcerated, who are living with HIV/AIDS, or are migrants.

Forms of Stigma

There are various forms of stigma that can arise both in themselves and towards people who have TB.

1. **Experienced stigma (Enacted or Experienced Stigma).** This stigma is related with behavior that is directly felt by someone who has TB. For example, someone who has TB is viewed with cynicism by their neighbors, considered bad by their family, or ostracized at work.

2. **Anticipated stigma.** This stigma is related to worry or fear of being treated differently because other people know that they have TB. For example, because of fear of being recognized as having TB, a person may decide not to go to a health facility to have their complaints checked or not take TB medication a health facility regularly. A person's worry about losing friends or being shunned by others when they find out about their illness is also included in this stigma.

3. **Internal or self-stigma.** This stigma occurs when people who have TB accept negative stereotypes and may act in accordance with these stereotypes. For example, someone who feels he has embarrassed his family and feels low value because he has TB.

4. **Secondary or external stigma.** This stigma is a negative attitude from the community or people around them that is experienced by family members, caregivers, friends or TB health workers because they live with or have close contact with people who have TB. For example, children of people who have TB are also ostracized from society.

The negative impact of TB stigma

Fear of being identified as someone with TB can lead a person to conceal their symptoms and avoid seeking care at a health facility, resulting in delays in diagnosis and treatment. Likewise, TB stigma can undermine treatment adherence, as patients worry that neighbors or friends will learn of their condition if seen visiting a health facility. Ultimately, TB that goes undiagnosed or inadequately treated leads to worse outcomes for the affected individual and greater transmission within the community.

TB stigma also generates feelings of shame and diminished self-worth, which heighten stress and contribute to further psychological and social difficulties. It can compound the challenges patients already face in coping with their condition, strain social relationships, impose financial burdens, and infringe on their rights and dignity. Together, these effects are associated with mental health problems such as anxiety and depression, and with reduced quality of life.

Stigma and Peer Support

Both anticipated and experienced TB stigma remain high in Indonesia. A study by Fuady and colleagues (2023) found that TB stigma is associated with depression and reduced quality of life among people affected by TB.

People affected by TB therefore need more than treatment alone. They also need social support from family, community, and peers. Peer support is considered particularly valuable because the relationship between peers is non-hierarchical, making it easier for individuals to express the challenges they face.

Despite this need, peer support remains limited for many people with TB, both globally and in Indonesia specifically — many who need it are not receiving the support they would like. One key issue is the absence of a formal support structure. Unlike support from health workers and health cadres — delivered through personal counseling or home visits, and already institutionalized — peer support tends to arise more organically within the community of those affected by TB.

Several initiatives have sought to formalize peer support, particularly for people with drug-resistant TB, often through regular group meetings. In these settings, people affected by TB can share experiences, concerns, and coping strategies. This approach is considered effective because it allows individuals to express themselves more openly, receive meaningful feedback from others with shared experience, and develop a sense of belonging rather than isolation.

2. THE CONCEPT OF PSYCHOSOCIAL SUPPORT AND PEER SUPPORTS

Introduction to the Concept of Peer Support (Peer Support)

What is a Peer Supporter?

Peers are individuals who share similar characteristics — typically comparable values, experiences, and life circumstances, and often belonging to the same age group.

What is Peer Support?

Peer support refers to services provided by individuals who share similar life experiences with those they serve, enabling them to help one another based on shared affiliation and a deep understanding of common experiences (SAMHSA, 2015). Such support can take many forms, including individual assistance, group discussions, advice-giving, tutoring, and other activities involving interpersonal engagement.

Peer Supporter Role?

Peer supporters listen, advise, refer, and provide general support. This role can be carried out individually or within group settings. Trained peer supporters may serve as facilitators or leaders in support group discussions, and can also help train new peer supporters to expand the network of support.

Why offer Peer Support?

Peer support programs help people with TB access disease-related information and psychosocial support, while also reducing social isolation and connecting people with TB — and their caregivers — with others facing similar health challenges (Hui Joo, 2022). Peer supporters can also serve as a listening ear when professional help is unavailable, inaccessible,

or not yet felt to be helpful.

Peers can also help people with TB as a listening ear when professional help is not available, inaccessible, or deemed not yet helpful.

What Kind of Help Does Peer Support Provide?

Peer supporters may offer several types of individual assistance, including:

- Talking with people affected by TB about the challenges they face related to their illness
- Exploring solutions to common problems together
- Helping to find and provide needed information
- Referring peers to other sources of support within the community

Three Important Characteristics of Being a Peer Supporter

Important Traits Used in Helping Skills

Adapted from Brown (1998).

Acceptance	Genuineness	Empathy
• **Accepting** people as they are<br>• **Respecting** individual beliefs<br>• **Appreciating** them and their views<br>• **Withholding judgement**<br>• **Listening actively**<br>• **Being attentive**<br>• Maintaining **confidentiality**	• Showing your true self<br>• Frankness<br>• Sincerity<br>• Honesty<br>• Transparency	• **Caring to understand** what is truly happening for a person.<br>• **Asking relevant questions** about a person's circumstances<br>• **Thinking, acting and feeling** in alignment with the person's feelings and interests<br>• **Refraining** from making assumptions or judgments

Acceptance

When helping others, it is important to show that, as a helper, you accept and respect them for who they are. They need to feel safe to share and explore their concerns without fear of embarrassment or judgment.

Sincerity (genuineness)

To help others effectively, you must be genuine — both as a person and as a peer supporter. A sincere, authentic presence builds the foundation of trust needed for a relationship of mutual assistance.

Empathy

A simple definition of empathy is the ability to put oneself in another person's shoes. Empathy is often confused with sympathy, but the two are different: empathy is not about feeling sorry for someone, becoming emotionally involved, or making assumptions about their experience. Rather, it means seeing things from another person's point of view, making a genuine effort to understand what things mean to them, and seeing the world as they see it.

Helping Through Self Help and Support Groups as Peers

1. **Providing support.** Mutual support among group members is essential so that no one feels alone in facing their problems. Every member has an equal opportunity to interact with and support one another.

2. **Obtaining information.** Group members exchange information with one another throughout the interaction process, helping each person better understand and process what is shared during sessions.

3. **Fostering a sense of belonging.** A sense of belonging develops through the ongoing exchange of information, including members sharing the conditions and experiences they face. This can cultivate a shared sense of responsibility for the problems at hand, motivating members to support one another.

4. **Sharing knowledge based on lived experience.** Through sharing and exchanges within the group, members contribute their own knowledge and enrich one another's understanding of the problems they face.

5. **Teaching coping strategies.** By sharing knowledge, support, and experience during discussions, the group helps members discover effective ways to cope with and overcome the challenges they face.

3. ACTIVE LISTENING, EFFECTIVE LISTENING, AND COMMUNICATION SKILLS

"The key to active listening is sincerity and a genuine desire to help others."

In active listening, the key lies in cultivating sincerity and a genuine desire to help others. Once we have prepared and demonstrated this sincerity in listening, we will naturally be able to listen to them actively.

Below are some detailed points that can help in the process of active listening:

1. **Let them speak** – Don't dominate the conversation when talking with the other person. We cannot listen well to someone if we keep talking ourselves. Focus your attention on the words, thoughts, and feelings of the person you're speaking with.

2. **Don't respond too quickly** – Avoid interrupting the other person, and give them the time and space to say what they want to say.

3. **Look at the person you're speaking with** – Observe their face, mouth, eyes, and hands, as well as the body language that helps them communicate with you. This can also help you concentrate, and it lets them feel that you are truly listening.

4. **Eliminate distractions** – Prepare yourself and your surroundings before the conversation begins. Keep your phone, computer, or anything else that might divert your attention out of reach while you talk.

5. **React to the idea, not the person** – Focus on what is being discussed. Don't let your personal feelings toward the speaker — for example, if you dislike them — influence how you respond to or interpret what they're saying. Their ideas may still be valuable, even if you don't like them as a person.

6. **Pay attention to their emotions while speaking** – Notice the speaker's emotions and behavior during the conversation, such as welling tears, trembling lips, or restless movement of the legs. This can strengthen your understanding of, and focus on, what they

are communicating.

7. **Allow time and space for reflection** – Conversations often pause — for instance, when someone is unable to continue speaking due to emotion, or hesitates to answer out of discomfort. Try to let that silence be, giving the other person time to gather their thoughts. If you have time constraints (for example, due to another commitment), communicate this limitation clearly at the outset.

8. **Avoid making assumptions** – Assumptions can hinder our ability to understand others. Don't assume that they:
   a. Use words in the same way we do
   b. Feel the same way we feel
   c. Are distorting the facts, simply because what they say doesn't align with what we think

   d. Are lying, simply because they interpret the facts differently than we do
   e. Are being unethical, because they seem to be trying to persuade us to agree with them
   f. Are angry, simply because they lack enthusiasm in expressing their views. Assumptions like these may sometimes be accurate, but more often they become an obstacle to understanding and to reaching compromise or agreement with the person we're speaking with.

9. **Resist the urge to solve the problem** – We are there to listen, not to solve the problems the other person is facing. Avoid focusing on finding answers or solutions, and only offer advice if it is genuinely requested.

Effective Listening

According to Egan (1994), conversation involves two important aspects:

1. Verbal messages
2. Non-verbal messages

It is important to hear and understand someone's core message, and then to communicate that understanding back to them clearly. A verbal message has three components:

1. **Experiences** – things that have happened to, or been experienced by, the person
2. **Behavior** – the things they choose to do or not do
3. **Feelings** – the emotions that arise

A problematic situation often becomes clearer when explored through these three components. For this reason, when your peer is speaking, try not to focus on how you will respond. Instead, listen carefully and identify the three parts of their verbal message. If someone is speaking only about their experiences, for example, you might gently ask about their behavior or feelings as well. Exploring all three components of a verbal message can help clarify the situation as a whole.

Listening is an active process. To listen effectively, it is important to check that the meaning we've understood is accurate. Understanding someone's situation well — and reflecting that understanding back to them — deepens empathy within the conversation.

The second aspect is non-verbal communication, through which we read a person's implicit cues and behavior. Recognizing these non-verbal messages is essential, but they should be neither over-interpreted nor overlooked.

Non-verbal behavior can shape a conversation in various ways — for instance, by denying, reinforcing, or creating confusion around the verbal message. With practice and experience, we become better able to read and interpret these cues across different situations.

Beyond understanding the body language of the person we're speaking with, we must also remain aware of our own. In a helping relationship, our non-verbal behavior and cues can shift

the meaning of the verbal messages we convey.

Important Non-Verbal Indicators:

- Body movements, such as posture, movements, and gestures
- Facial expressions, such as smiling, frowning, raised eyebrows, a tilted mouth, or grimacing
- Voice-related behavior, such as tone, pitch, volume, and intensity
- Observable autonomic physiological responses, such as rapid breathing, rashes, flushing, paleness, or temporary pupil dilation
- Physical characteristics, such as fitness level, height, weight, and complexion
- General appearance, such as grooming and clothing

Communication Skills

Reflecting & Paraphrasing

Reflection is an important tool used to convey the meaning and feelings behind what someone says (Gilliland & James, 1993; Moursund, 1990). Reflecting on the content of a conversation typically involves repeating the last few words the speaker said, communicating that their point of view has been understood.

Example:<br><br>A friend who had just been diagnosed with TB was anxious about telling his family. He says to you, "I can't do that. I'll meet them and just stay silent!"<br><br>Possible reflection:<br><br>*"...and just stay silent?"*

Paraphrasing is the ability to restate what someone has just said in your own words (Gilliland & James, 1993; Moursund, 1990). In conversation, listeners may repeat the message using their own phrasing, or draw on concrete illustrations, examples, or metaphors to convey their understanding. Paraphrasing brings together thoughts, feelings, and actions to create a clearer picture for both people in the conversation. It also allows speakers to hear their own statement reflected back, reassuring them that they are being listened to well — making paraphrasing a valuable form of feedback during a conversation. Beyond simply reflecting what was said, effective paraphrasing also invites the speaker to explore the topic more deeply or understand it more fully.

Example:<br><br>A person with TB described a heated argument with his father, who had refused to lend him money for TB treatment. As he spoke about his anger, his eyes filled with tears.<br><br>Possible paraphrase:<br><br>*"You're angry at your father for not lending you the money, and at the same time, you feel hurt."*

This paraphrase accounts for the content (the father's refusal to lend money), the feelings

expressed verbally (anger), and the non-verbal behavior observed (tears).

For paraphrasing to be effective, it must be reasonably accurate. Even so, you should still attempt to paraphrase what someone has said, even when you're unsure whether you've understood correctly. Giving the other person the chance to confirm or correct your paraphrase is valuable — it allows you to catch and correct any misperceptions.

Further Examples:

1. "I really can't come to the family meeting. It's not that I don't want to — I just can't stand the thought of being asked about my illness." Paraphrase: "It sounds like you want to come to the family meeting, but you're staying away because you're afraid of being asked about your illness."

2. "I'm confused. I don't know whether I still need to come to the clinic or not." Paraphrase: "It sounds like you're unsure whether you still need to come to the clinic."

Guidelines for paraphrasing:

- **Be tentative** and **share your impression** of what the other person said.
- **Avoid telling** or **defining** other people's perceptions.
- **Be respectful**: don't judge, ignore, or use sarcasm.
- **Use your own words**. Repeating exactly what the speaker said (parroting) is not paraphrasing.
- Try to **adapt to other people's language**. Use appropriate, non- offensive words when describing an event or situation, for example. "injured" is not "disabled".
- **Listen to how deep the feelings are expressed** in the person's voice and reflect on your response accordingly.
- **Don't add to what the person says**, and avoid interpretations and evaluation.
- **Be sincere** and **don't pretend to understand** if you don't.
- Paraphrase **briefly and directly.**

Summarizing

Summarizing involves gathering the main points of a discussion and organizing them so they can be reviewed, confirmed, or corrected (Moursund, 1990). Summarizing a conversation can serve several purposes, including:

- Prioritizing and focusing thoughts and feelings
- Closing a discussion on a particular theme
- Opening the door to further discussion or transitioning to a new theme
- Checking understanding of the conversation so far
- Quickly exploring an idea in depth
- Refocusing a conversation that has begun to drift or lose direction
- Considering ways to move the conversation forward

Example:

In the response below, the listener summarizes what their partner has said and adds a question to help the conversation move forward (Moursund, 1990):

"May I check that I understand you correctly? You've mentioned a few options you're considering — handling things on your own, making an appointment with a psychologist, or joining a counseling group — but none of these feels like the right solution to you. What do you see as the advantages and disadvantages of each?"

Barriers to Communication

When a conversation touches on a familiar or repetitive topic, listeners run the risk of a communication breakdown by falling into unhelpful response patterns. Common barriers to effective listening include:

a. **Comparing** – When someone shares something, we may find ourselves comparing their situation to our own. For example: *"I actually went through something worse than this."*

b. **Mind-reading** – While someone is speaking, we may jump to assumptions about their

situation rather than listening to what they're actually saying. For example: *"He keeps complaining about the doctor, even though I think the doctors are doing a great job — maybe he just has a personal issue with this one."*

c. **Rehearsing** – We may begin constructing imagined scenarios of how the conversation will unfold, becoming so absorbed in planning our response that we stop paying attention to what's actually being said. For example: *"If I respond this way, he'll probably deny it."*

d. **Filtering** – We may selectively attend to only the parts of the story we're interested in hearing, tuning out the rest.

e. **Judging** – We may enter a conversation with a fixed opinion of the speaker, which then colors how we interpret everything they say. For example: assuming someone is "too spoiled," and attributing everything they say to that trait.

f. **Being reminded of one's own experience** – Something the speaker says may trigger memories of a similar experience of our own, pulling our attention away from them and into our own thoughts.

g. **Interrupting to relate or advise** – When a story reminds us of something in our own lives, we may be tempted to interrupt and redirect the conversation toward ourselves, or to jump in too quickly with suggestions and solutions — taking over the conversation before the speaker has finished, even though our advice may not be what they actually want.

h. **Being judgmental or dismissive** – We may respond to someone's opinion or story by openly disagreeing and using words that belittle them. For example, responding to someone's struggle to earn an income due to illness with: *"You're just not trying hard enough — that's why you should have studied technology so you could sell things online."*

i. **Being defensive** – We may become so convinced we're right that we're unable to accept feedback or correction from others, closing ourselves off to reflection and adjustment.

j. **Losing interest** – When a conversation continues for a while, we may grow bored and try to change the subject or make a joke to relieve that boredom.

k. **People-pleasing** – We may feel a strong urge to make the other person happy, leading us — consciously or not — to agree with everything they say, even when we suspect it isn't entirely accurate, without genuinely evaluating the truth of what's being said.

a. When someone expresses an opinion or tells a story, we express our disagreement with his thoughts and **argue using words that demean him**. *For example: when someone tells us about their difficulty in earning an income because of their illness, we will say "you are the one who is not trying enough, that's why you have always studied technology so you can sell in online shops."*

b. We have the potential to have an attitude of **feeling right that** we cannot accept criticism or correction from others in order to reflect and adjust.

c. In a conversation that is taking place, we have the potential to feel bored so we try to **change the topic** or make a joke to relieve that boredom.

d. We potentially want to make other people happy so that consciously or unconsciously, we **tend to agree with everything they say** even though we realize that not everything is good. **We tend not to try to think about the truth of the ideas conveyed.**

4. USING CLOSED AND OPEN QUESTIONS

In mentoring peers and facilitating group meetings, the skill of asking questions is essential to master. Well-phrased questions support the mentoring process, while poorly phrased ones can hinder it — even making communication more difficult. For this reason, questions should be used thoughtfully to genuinely benefit the conversation.

Guidelines for Asking Questions

Have a purpose. Ask questions with a clear intent, so the direction of the conversation remains well-defined.

Have substance. Avoid empty questions. Substantive questions help elicit meaningful answers, gather useful information, and encourage the other person to reflect.

Take your time. Don't rush into asking questions — proceed slowly and gradually. Many people need to build trust before they feel comfortable answering, especially when asked about something personal.

Ask in moderation. Avoid asking too many questions in succession, so the other person doesn't feel "interrogated."

We should also be aware of certain question types that have the potential to cause problems:

a. **Leading questions.** These questions are typically built on assumptions already formed in our minds, as though we already know the answer or expect the other person to respond in a particular way. For example: *"Isn't that easy? When are you going to tell your partner about your illness?"*

b. **"Why" questions.** The open-ended "why" question can be useful for exploring someone's reasoning. However, it can also make people defensive — especially when delivered with an interrogative tone — as it may imply that the person should already know the answer, or that we doubt their judgment. For example: *"You said you couldn't get over your feelings of guilt. Why? Why don't you understand your own feelings?"*

c. **Overly intimate questions.** Questions that are intimate or overly personal, without clear context or relevance, are inappropriate. This can be difficult to recognize in mentoring, particularly with someone experiencing stigma, discrimination, or mental health difficulties. It's important to build toward more intimate topics gradually, using earlier questions to open the conversation naturally. We should always remain respectful and treat others as we would wish to be treated ourselves — avoiding any questions of a sexual or voyeuristic nature. For example: *"If you're having problems with your husband because of this illness, what do you do when you're alone in the room?"* This question might seem reasonable in the context of exploring how relationships have changed following a TB diagnosis, but it becomes problematic due to its sensitive and intrusive nature.

d. **Untimely or interrupting questions.** We often feel the urge to ask about something that occurs to us mid-conversation. However, doing so can disrupt the natural flow of someone's story. Beyond being impolite, it can also abruptly cut the conversation short. For example: *"If they act like that, what do you do?"* (asked in the middle of someone describing their experience of being abandoned by friends).

Closed and Open Questions

When asking questions, we can draw on two main types: closed questions and open questions.

Closed Questions

Closed questions often begin with words like "whether," "could," or "would," and can typically be answered with a simple "yes" or "no." These are useful when we need specific factual information, or when checking facts and seeking clarification.

When used appropriately, closed questions can be useful in the following ways:

1. **Clarification.** For example: *"So, are you feeling stressed about this person's attitude?"*
2. **Checking information.** For example: *"It sounds like you're saying you don't like how they've been treating you — is that right?"*
3. **Confirming facts.** For example: *"Did you go to the health center after you noticed the bloody cough?"*

4. **Focusing attention on a specific issue.** For example: *"You mentioned earlier that you weren't invited to eat with friends at work — is that something that bothers you specifically?"*

However, closed questions can limit discussion, since they tend to produce only one-word answers. For example: *"Are you satisfied with the health services at the Puskesmas?"* The person will typically answer only "yes" or "no" — and the conversation ends there.

Closed and Open Questions: Notice the Difference!

A woman, approximately 30 years old, has just been diagnosed with TB. She seems anxious about her illness and feels that her friends, relatives, and colleagues have begun to distance themselves from her because of her frequent coughing. She works as a cashier at a leading supermarket, and her boss has told her that if her cough doesn't improve within the next week, she will have to be let go.

She has tried her best to treat the cough with various medications, but it hasn't improved. When she went to the community health center, she was told she might have TB, and that if diagnosed, she would need to undergo six months of treatment. This news made her even more anxious, as she fears losing her job — despite being the main breadwinner for her family. She also feels guilty for not eating enough or regularly, and has recently started drinking alcohol.

Examples of Closed Questions:

- Do you share your concerns with your friends?
- How many people work around you?
- Do you enjoy your job?
- Are there other workers who cough like you?
- Does your family know about your possible illness?

Examples of Open Questions:

- What do you think might help reduce these concerns?

- How have your co-workers treated you so far?
- How happy are you with this job — can you tell me more?
- What would you do if other employees had a similar cough?
- How would you like to tell your family about the possibility of this illness?

Open Questions

Open questions are used to elicit more complete and meaningful responses, particularly when we need someone to explain or describe something in greater depth. These questions encourage the other person to explore and share their story, helping to bring more clarity and detail to the issues being discussed. When used well, open-ended questions help someone articulate their specific experiences, behaviors, and feelings.

Suggestions for Using Open-Ended Questions:

1. **To open a discussion**, use question words like "What" (rather than "whether") and "How." For example: *"How did you feel when you heard the TB diagnosis? How did you feel when the officer shared the test results with you?"*
2. **To ask for a description**, phrase the question as a request. For example: *"Can you describe what you thought when you saw blood while coughing?"*
3. **To give someone space to elaborate**, invite them to expand on what they've said. For example: *"You mentioned feeling shocked by the diagnosis. Can you tell me more about what you felt at that moment? When do these feelings tend to arise?"*
4. **To focus on feelings.** For example: *"How did you feel when you were treated that way?"*
5. **To focus on next steps.** For example: *"Now that you know treatment will take six months, what are you planning to do?"*

5. ROLES AND LIMITATIONS, CHALLENGING SITUATIONS, AND CONFIDENTIALITY

Roles and Boundaries in the Helping Relationship

- Be aware of both your own values as a supporter and those of your peers, as well as the mentoring process itself
- Be clear about the knowledge and experience you bring
- Be firm about confidentiality and boundaries
- Remember that the relationship and conversation are about the other person, not about yourself
- Ensure that any self-disclosure is done appropriately
- Seek help or advice when you feel unsure or overwhelmed
- Refer peers to mental health professionals when necessary
- Understand the limits of what you can offer in supporting people with TB
- Feel comfortable discussing the scope of your involvement, and be willing to say no when necessary
- Take care of yourself throughout the process of supporting people with TB

Confidentiality

Peer supporters must understand when confidentiality should be maintained and when it may need to be broken. As a general rule, confidentiality should always be maintained unless doing so risks direct harm to the person with TB or to others. For this reason, peer supporters are responsible for clarifying the boundaries of confidentiality with their peers at the outset of the mentoring relationship.

If a person with TB discloses information you would normally keep confidential, but you feel it may need to be shared, assess the situation by asking yourself:

- Why am I considering breaking confidentiality?
- How serious is the situation?
- What risks might my peer face?
- Do their actions put others at significant risk?
- If I disclose this information, what are the potential positive and negative consequences?
- What impact could this have — on others, and on myself?

If you have a clear and well-founded reason to breach confidentiality, try to limit any negative consequences by:

- Explaining to the person why you are breaking confidentiality
- Clarifying the following:
- Why are you breaking confidentiality?
- Who will you tell?
- What will you say to them?
- What might they do with this information?
- What consequences might this have for the person involved?

When facing a difficult decision about breaching confidentiality, **discuss the situation with a qualified professional** — such as a counselor or doctor — without disclosing names or identifying details, in order to preserve confidentiality. They can help you determine the most appropriate course of action. If you feel overwhelmed by the information you've received, seek support for yourself as well, whether from a trusted adult or another professional.

Handling Disclosure

Here are some general suggestions for responding to someone who shares their problems with you:

- **Listen carefully.** Your support and encouragement matter a great deal.
- **Know your capabilities and limits.** Some situations are beyond what you can — or should — handle on your own. If in doubt, refer the person to a professional.
- **Seek consultation when needed.** Let them know you'd like to speak with someone more knowledgeable before offering direct advice. Consult that source, then follow up with the person as soon as possible.
- **Maintain confidentiality**, and reassure them of this — unless you believe that doing so could put them or someone else at risk.
- **Continue to offer support as needed.** Make sure they know you're available and will support them as they work through their problems. That said, there may be times when you're unable to remain available, and it's important that they know this in advance, so they can turn to other trusted people for support.

6. MOTIVATING SKILLS

Motivation in Mutual Support Groups

Motivation can be understood as the internal drive that moves a person toward a particular goal or change in behavior. In the context of mutual support and peer groups, motivation plays a central role in helping members move from simply understanding their condition to actively taking steps toward managing it — whether that means adhering to TB treatment, disclosing their diagnosis to family, or making lifestyle changes that support recovery. Because motivation can fluctuate depending on a person's emotional state, environment, and life circumstances, facilitators play an important role in nurturing and sustaining it throughout the mentoring process, rather than assuming it is fixed or one-time achievement.

The principles needed to carry out motivational skills are:

1. Focus on the participant's current condition (ask)
2. Obtain permission to give suggestions to participants (offer)
3. Focus on the participant's future, such as the impact of the advice we give, the way the participant implements the suggestion, the participant's next plans after meeting us.

The main role of a facilitator is to guide participants or members, which requires building strong communication so that members feel free to share their full story about their current circumstances — and so that the motivation offered can be tailored to their actual needs. Other essential skills for building motivation include giving clear direction (*directing*), and listening closely to understand and follow the natural flow of what participants are sharing (*following*).

The Keys to Building Motivation

There are four key elements to fostering motivation:

1. **Acceptance.** Facilitators need the ability to accept members as they are, in order to understand their circumstances and guide them toward change.

2. **Collaboration.** Motivation is built collaboratively between facilitator and member, working together to develop strategic steps the participant can use to make meaningful changes.

3. **Understanding the context.** Facilitators must understand the participant's context and circumstances in order to tailor their guidance effectively, helping direct participants toward the changes they hope to achieve.

4. **Understanding potential** Facilitators must understand each participant's capabilities, so that the guidance offered aligns with what the participant is realistically able to implement — helping them work toward achievable, realistic goals.

7. RECOGNIZING AND RESPONDING TO DISTRESS, INCLUDING REFERRAL TO A MENTAL HEALTH PROFESSIONAL FOR FURTHER SUPPORT

What, when, and where will refer

What

Making a referral means helping someone access other, more professional sources of support.

When

1. The problem requires the help of a mental health professional
2. You identify that it difficult to handle by yourself while you can’t be sincere, honest, and impartial anymore
3. You are worried the person may harm themselves or become more deeply entrenched in their problem
4. You are concerned about your own mental health condition due to the burden you have to dela with
5. The issue falls into one of these categories requiring urgent professional help:
    - Depression and/or thoughts of ending one's life
    - Extreme stress, anxiety, or panic
    - Prolonged grief
    - Alcohol or substance use problems
    - Eating disorders

- Legal issues
- Family difficulties or problems

Where to Refer

Mental health professionals at nearby hospitals or secondary level of care.

How to tell the participant you are planning to make a referral

- Make sure you understand the problem clearly before making a referral
- Choose your words carefully
- Consider using phrases such as:
    - "You may find it helpful if..."
    - "You might consider talking to..."
    - "Would you be open to talking to...?"
    - "I think this could be useful for you..."
    - "I think it would help to meet with..." or "...to talk to..."
- Be open and honest with them — explain why you may not be the best person to help, or why someone else may be better positioned to help at this time
- You can continue to offer support by staying in touch and checking in on how things are going
- Know your limitations, and don't try to go beyond your capabilities
- Remember that, ultimately, this is the other person's problem to work through, not yours — don't feel overwhelmed or guilty if you're unable to help further

Tips for Making Referrals When a Peer Is Reluctant

If you believe a peer would benefit from professional help, be honest about your reasons and share your concerns about the issue they're experiencing openly.

Sometimes people feel reluctant or embarrassed to accept help. Here are some suggestions:

- Dispel common myths about seeking help. Explain that speaking with a counselor or mental health professional can be beneficial, and doesn't imply that someone is experiencing a serious mental health disorder. *[Reviewer note: this section needs rewriting for clarity.]*
- Encourage your peer to schedule an appointment with a professional
- Frame seeking help as a positive step, not a last resort
- Some people may feel uncomfortable seeing a counselor or mental health professional, but may be open to seeing a general practitioner instead. Others may prefer speaking with a religious leader or joining a support group. It's helpful to present all available options and discuss them together.
- If a peer is reluctant to seek help, ask what's behind that hesitation — it may stem from a previous negative experience, or simply a lack of understanding about what to expect
- Exploring the source of their reluctance may help you address their specific concerns
- Be clear that the issue falls outside your own area of expertise
- If they're not ready to seek help now, consider giving them a name and contact number they can use later, when they feel ready
- Offer to help them schedule an appointment
- If you do arrange an appointment, share any specific concerns you have with the professional involved
- If the situation feels urgent — for example, if you believe there is a risk of harm to the peer or others — and the person remains reluctant to see a professional, you may need to speak with someone else who can help.

8. SKILLS FOR GUIDING SUPPORT GROUP

By the end of this section, participants should be able to:

1. **Describe the facilitator's role in guiding a support group** — understanding the balance between active guidance and allowing members to drive group dynamics, without acting as either a dictator or a passive observer.
2. **Identify the key steps in starting a support group session**, including setting a flexible schedule, establishing a shared mission or goal, distributing responsibilities among members, and building group identity.
3. **Recognize the four core skill areas needed to run and maintain an effective session** — communication, affective (emotional) skills, relationship-building, and problem-solving — and give examples of how each is applied in practice.
4. **Anticipate common challenges that can disrupt a support group's progress**, such as inconsistent attendance, premature labeling of new members, emotional strain from more severe cases within the group, and the risks of facilitation by an inexperienced or unqualified facilitator.
5. **Understand the boundaries of the facilitator's role**, including recognizing when a group's needs exceed the facilitator's competence and require referral to a more qualified professional.

The process of driving a support group

The facilitator's role is to guide the group toward its goals and to introduce activities that support that progress. A facilitator is neither a dictator nor a passive observer — they must work actively to help every group member engage and play an active role in the group's dynamics. Below are several key elements of guiding a support group:

Starting a Session

When beginning a session, several steps can help ensure that all group members participate meaningfully and work toward the group's shared goals:

a. Set a schedule

A regular meeting and discussion schedule helps group counseling run smoothly. That said, the schedule should be based on the availability and preferences of group members, and should remain flexible, ideally revisited after each session. Members should also be encouraged to arrive on time as a shared commitment to the group.

b. Establish a shared mission or goal

This helps keep discussions focused during each session. The mission or goal should be determined collectively, based on the agreement of all members, and should remain flexible over time. An example of a shared goal might be: *"To feel more confident, despite living with TB."*

c. Share responsibilities

Distributing responsibilities among group members can increase participation. Responsibilities — including the facilitator's own — should be shared as equally as possible. Examples of tasks that can be rotated include leading the next session, choosing the discussion topic, proposing the time and location, or leading an icebreaker activity.

d. Give the group a name

Naming the group can help build a sense of togetherness and shared ownership among members.

Running And Maintaining Sessions

To run an effective session, a facilitator must continually develop and refine their guiding skills. These generally fall into four categories: communication skills, affective (emotional) skills, relationship-building skills, and problem-solving skills. Mastering these skills helps ensure that group dynamics function well.

1. **Communication skills** — the facilitator's ability to communicate effectively with the group.
2. **Affective skills**, which include:
    a. Recognizing and exploring feelings
    b. Gathering feedback
    c. Scanning the group for emotional cues
    d. Recognizing non-verbal cues
    e. Recognizing reluctance or hesitation
    f. Validating feelings
    g. Helping the group sit with and process feelings
    h. Interpreting or translating feelings for the group
3. **Relationship-building skills**, which include:
    a. Establishing boundaries
    b. Setting structure
    c. Clarifying roles
    d. Encouraging consensus
    e. Making connections between members' experiences

f. Identifying shared similarities among members

g. Encouraging members to take on active roles within the group

4. **Problem-solving skills**, which include:

a. Identifying decision-related needs and supporting implementation

b. Clarifying

c. Offering suggestions

d. Determining focus and priorities

e. Addressing problems directly

Through these communication skills, the facilitator can help shape and improve interactions among individuals within the group.

Reasons a Support Group May Not Go According to Plan

- **Unpredictable attendance.** When members attend inconsistently, other members may feel less comfortable sharing personal issues with people they don't know well.
- **Premature labeling of new members.** Established members may quickly — and sometimes negatively — label newer members. For example, if a new member shares difficulty adhering to their treatment, other members might label them as non-compliant, which can reduce the new member's motivation and increase feelings of stigma.
- **Disproportionate impact from more severe cases.** If one group member's condition is notably more advanced, it may cause distress or fear among members who are earlier in their own journey. Intense emotional expression from one member can also unintentionally burden others who are trying to cope. For example, if one participant repeatedly cries and draws the group's attention, other members may feel uncomfortable.

- **Misinformation or poor guidance from an inexperienced facilitator.** A facilitator lacking the skills to guide and direct the group effectively can cause sessions to veer off track.
- **Facilitators being asked to provide therapy or treatment beyond their competence.**

Guiding a support group effectively requires more than good intentions — it calls for intentional structure, a defined set of interpersonal skills, and ongoing self-awareness on the part of the facilitator. A well-run session begins with clear, shared expectations around scheduling, goals, and responsibilities, and is sustained through communication, emotional attunement, relationship-building, and problem-solving skills that the facilitator continues to develop over time.

Just as important as these skills, however, is recognizing their limits. No facilitator is expected to manage every challenge a group may face alone. Knowing when a situation calls for additional support — whether from a more experienced facilitator, a mental health professional, or another qualified resource — is itself a core part of responsible facilitation, not a sign of failure.

Support groups will not always unfold smoothly, and that is expected. Attendance may be inconsistent, group dynamics may shift, and difficult emotions will surface. These moments are not signs that a group has failed, but opportunities for the facilitator to apply the very skills outlined in this section — patience, structure, and a willingness to guide the group back toward its shared purpose.

REFERENCES

Fuady, A. et al. (2024) 'Stigma, depression, quality of life, and the need for psychosocial support among people with tuberculosis in Indonesia: A multi-site cross-sectional study, PLOS Global Public Health, 4(1). doi:10.1371/journal.pgph.0002489.

Core competencies for peer workers in Behavioral Health Services - Samhsa (no date) www.samhsa.gov.Available

at:https://www.samhsa.gov/sites/default/files/programs_campaigns/brss_tacs/core--competencies_508_12_13_18.pdf (Accessed: 19 January 2024).

Joo, J.H. et al. (2022) 'The benefits and challenges of established peer support programmes for patients, informal caregivers, and healthcare providers', Family Practice, 39(5), pp. 903-912. doi:10.1093/fampra/cmac004.

Directorate General of Disease Prevention and Control, K. K. R. (2020). Tennis Instructions for Supporting People with Drug-Resistant TB by the Community.

Kracen, A., Naughton, A., O'reilly, J., Panoutsakopoulou, V., & Rooney, N. (2003). Peer Support Training Manual Written and compiled by Staff Members of the Student Counselling Service. http://www.tcd.ie/Student Counselling/

Menaldi, A., Hanum, L., & Asih, S. R. (n.d.). Support Group Guiding Skills Refresher Module. CEPAT- LKNU & Faculty of Psychology UI.
