# Supplementary material for: Community-based, peer-led psychosocial support to address stigma and reduce depression among adults with tuberculosis in Indonesia: A prospective interventional cohort study
Source: PLOS Glob Public Health. 2026 Jul 16;6(7):e0006754. doi: 10.1371/journal.pgph.0006754 (PMC13375138; doi:10.1371/journal.pgph.0006754)
Supplement: S3 File — Panduan Dalam Memberikan Dukungan Psikososial Bagi Pejuang TB (PDF in Bahasa Indonesia). (PDF) [file pgph.0006754.s003.pdf]

UNIVERSITAS INDONESIA
Veritas, Probitas, Iustitia
UI PUBLISHING

Handout Materi Pelatihan

Dukungan Teman Sebaya

Panduan Dalam Memberikan
Dukungan Psikososial Bagi Pejuang TB

TB CAPS

Fakultas Kedokteran
Universitas Indonesia

Primary Health Care
Research and Innovation
Center Cluster IMERI FKUI

Liverpool School of Tropical Medicine, Liverpool, UK.

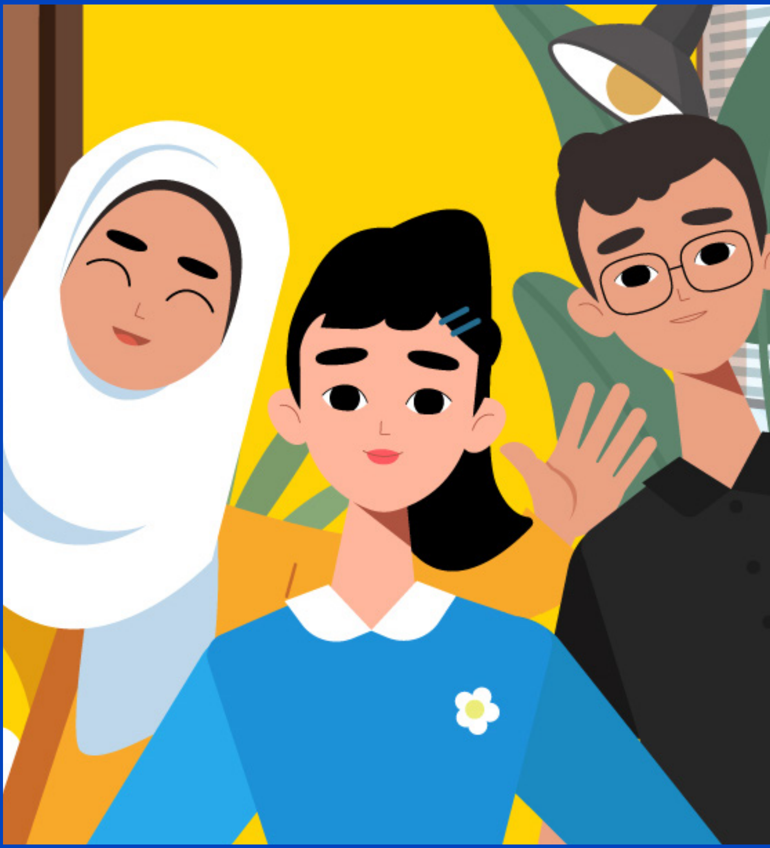

Handout Materi Pelatihan

Dukungan Teman Sebaya

Panduan Dalam Memberikan
Dukungan Psikososial Bagi Pejuang TB

dr. Ahmad Fuady, M.Sc, Ph.D
dr. Trevino Pakasi, Sp. KKLP., Ph.D
Dr. dr. Finny Fitry Yani, SpA(K)
dr. Marinda Asiah Nuril Haya, Ph.D
dr. Feranindhya Agiananda, Sp. KJ
Matsna Haniifah, S.K.M, M.P.H
dr. Mariska Anindhita
dr. Artasya Karnasih, Sp. KJ
Imelda Aliska, S.K.M, M.Epid
Dr. Tom Wingfield, MbChB(Hons), PhD, FRCP, DTMH, DipHIV DELE, PGCMedE

UNIVERSITAS INDONESIA
Veritas, Probitas, Iustitia
UI PUBLISHING

Materi Pelatihan: Dukungan Teman Sebaya
Panduan Dalam Memberikan Dukungan Psikososial
Bagi Orang Dengan Tuberkulosis

Penulis:
dr. Ahmad Fuady, M.Sc, Ph.D
dr. Trevino Pakasi, Sp. KKLP., Ph.D
Dr. dr. Finny Fitry Yani, SpA(K)
dr. Marinda Asiah Nuril Haya, Ph.D
dr. Feranindhya Agiananda, Sp. KJ
Matsna Haniifah, S.K.M, M.P.H
dr. Mariska Anindhita
dr. Artasya Karnasih, Sp. KJ
Imelda Aliska, S.K.M, M.Epid
Dr. Tom Wingfield, MbChB(Hons), PhD, FRCP, DTMH, DipHIV DELE, PGCMedE

Editor:
Matsna Haniifah, S.K.M, M.P.H
dr. Mariska Anindhita

Pengarah:
Dr. Tom Wingfield, MbChB(Hons), PhD, FRCP, DTMH, DipHIV DELE, PGCMedE

Penanggung Jawab:
dr. Ahmad Fuady, M.Sc, Ph.D

ISBN : 978-623-333-797-7
e-ISBN : 978-623-333-798-4 (PDF)
vi + 54 hlm.; 15,5 x 23 cm

©Hak Cipta Dilindungi Undang-Undang
Dilarang mengutip, memperbanyak dan menerjemahkan sebagian atau seluruh isi buku ini tanpa izin tertulis dari penulis dan penerbit

Cetakan 2024

Diterbitkan pertama kali oleh UI Publishing
Anggota IKAPI & APPTI, Jakarta
Jalan Salemba 4, Jakarta 10430
Tel. +62 21 319-35373; 319-30172; 319-30252
Kompleks ILRC Gedung B Lt. 1 & 2 Perpustakaan Lama Universitas Indonesia Kampus UI, Depok, Jawa Barat 16424
Tel. +62 21 788-88199; 788-88278
E-mail: uipublishing@ui.ac.id
Website: www.uipublishing.ui.ac.id

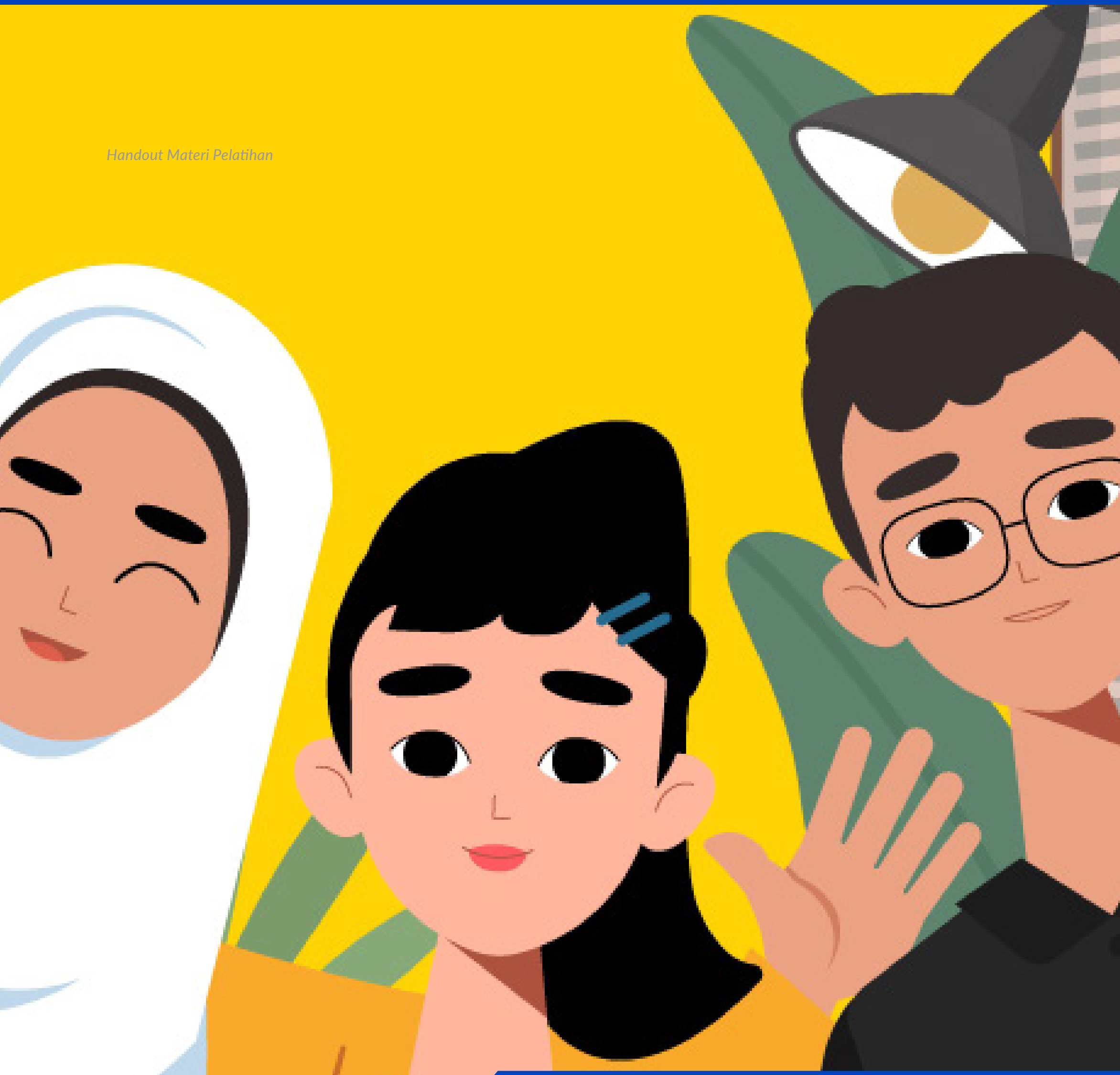

“Dalam lanskap kesehatan dan kesejahteraan yang dinamis, pentingnya dukungan teman sebaya menjadi kebutuhan yang harus dipenuhi. ”

Daftar **Isi**

Kata Pengantar
vi

01. Workshop 1
TBC, Stigma, dan Psikososial
1 - 5

02. Workshop 2
Konsep Pendampingan Psikososial dan Dukungan Teman Sebaya (*Peer Support*)
6 - 14

03. Workshop 3
Mendengarkan Aktif, Mendengarkan yang Efektif, Teknik Komunikasi
15 - 24

04. Workshop 4
Pertanyaan Tertutup dan Terbuka
25 - 30

05. Workshop 5
Peran dan Batasan, Situasi yang Menantang, dan Kerahasiaan
31 - 36

06. Workshop 6
Keterampilan Memotivasi
37 - 38

07. Workshop 7
Mengenali dan Merspons Masalah Mental, Kapan Kita Perlu Mencari Pertolongan dan Merujuk Pada Profesional Kesehatan Jiwa
39 - 45

08. Workshop 8
Keterampilan Memandu Dukungan Kelompok
46 - 49

09. Workshop 9
***Self-Care* (Perawatan Diri)**
50 - 52

Referensi
53 - 54

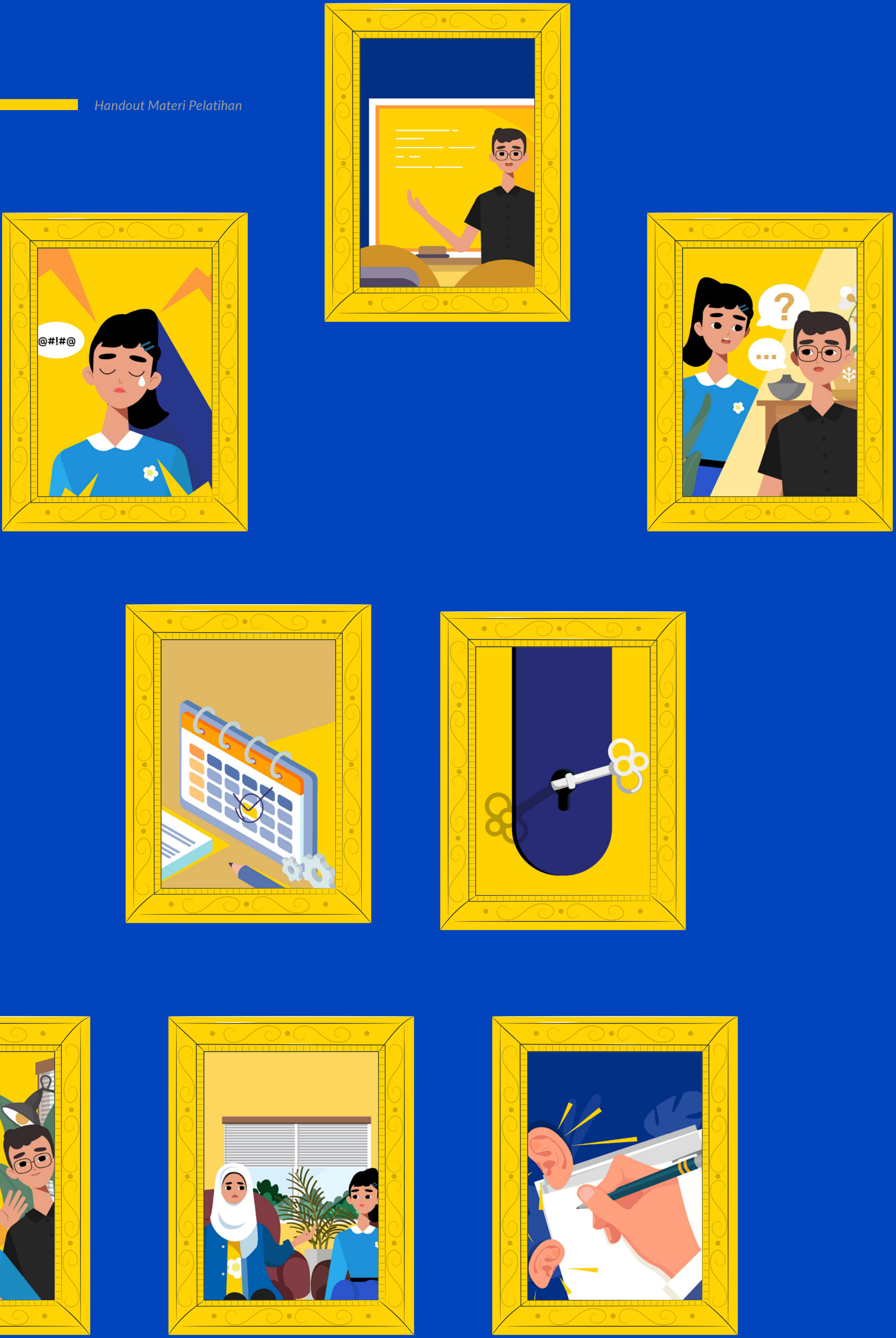

Kata Pengantar

Dengan senang hati kami memperkenalkan modul Dukungan Teman Sebaya ini. Dalam lanskap kesehatan dan kesejahteraan yang dinamis, pentingnya dukungan teman sebaya menjadi kebutuhan yang harus dipenuhi. Modul ini berfungsi sebagai pedoman dalam membangunpemahaman yang utuh mengenai peran dan pemberdayaan teman sebaya.

Dalam perjalanan dalam menyediakan layanan kesehatan holistik bagi orang dengan tuberkulosis (TBC), kita sering kali menemukan bahwa dukungan paling besar berasal dari mereka yang telah menempuh jalan serupa sebelumnya. Dalam hal ini, mereka ada teman sebaya yang pernah mengalami TBC atau dikenal dengan penyintas TBC. dukungan teman sebaya bagi orang dengan TBC dapat melampaui kerangka tradisional mengenai teman sebaya, namun juga mengakui dan memanfaatkan kekuatan yang tersedia dalam masyarakat dan potensi perubahan positif yang dapat digali melalui pengalaman bersama.

Modul ini bukan sekedar kumpulan pelajaran. Buku ini juga merupakan ajakan untuk mengeksplorasi kedalaman dan kekayaan dukungan teman sebaya dalam berbagai konteks. Buku ini berisi wawasan yang dapat menginspirasi, memberi informasi, dan membimbing siapapun yang membacanya, baik profesional kesehatan, aktivis dukungan teman sebaya atau penyintas TBC, kader kesehatan, atau seseorang yang mencari dukungan. Modul ini disusun melalui proses identifikasi kebutuhan, pengkajian, dan masukan dari modul-modul serupa yang telah dijalankan. Dengan demikian, modul ini diharapkan dapat memanfaatkan potensi sebenarnya dari teman sebaya dalam mendukung penyediaan dukungan psikososial bagi orang dengan TBC.

Tim Penyusun mengucapkan terima kasih yang sebesar-besarnya kepada berbagai pihak berdedikasi yang telah menyumbangkan keahlian, wawasan, dan semangat hingga akhirnya modul ini dapat tersusun dengan baik. Kami berharap agar modul ini menjadi sumber inspirasi, pendorong, perubahan positif dalam penanggulangan dan kontrol TBC di Indonesia, dan tentu saja mewujudkan cita-cita dalam menciptakan dunia yang aman dari TBC melalui kontribusi nyata dari teman sebaya.

Tim Penyusun

01.

Workshop 1
TBC, Stigma, dan Dampak Psikososial

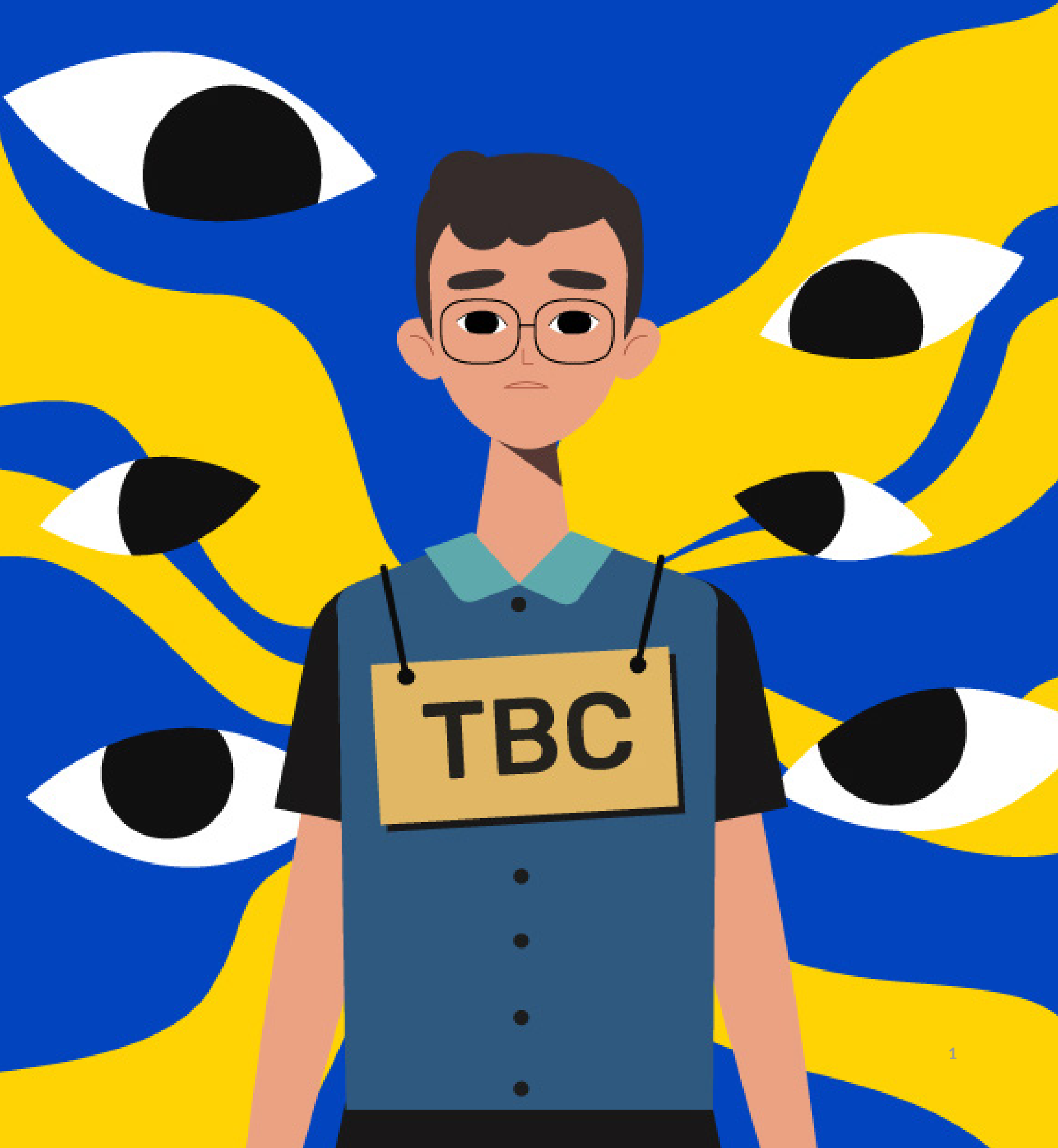

Tuberkulosis, Stigma, dan Dukungan Teman Sebaya *(Peer Support)*

Tuberkulosis (TBC) adalah penyakit infeksi kronik yang pada umumnya menyerang paru-paru. Jumlah orang yang terdiagnosis TBC mencapai 10 juta setiap tahun dan menyebabkan satu juta kematian setiap tahun di dunia. Penyakit TBC di Indonesia masih sangat tinggi, diperkirakan mencapai 939.000 kasus per tahun, dan ini menyebabkan Indonesia menjadi negara dengan jumlah kasus TBC kedua tertinggi di dunia. Masalah TBC tidak hanya berkaitan dengan ketersediaan vaksin dan obat-obatan, tetapi juga berkaitan dengan masalah sosial yang sering timbul ketika seseorang mengalami TBC. Salah satu masalah sosial tersebut adalah stigma.

Tuberkulosis dan Stigma

Istilah ‘stigma’ mengacu pada “pengucilan, penolakan, mempersalahkan, atau menurunnya nilai seseorang yang timbul dari pengalaman atau kekhawatiran terhadap pandangan sosial yang merugikan”. Demikian pula, stigma mengacu pada suatu proses sosial yang kompleks dan melekat ketika seseorang mengalami penyakit tertentu, termasuk TBC. Misalnya, orang yang mengalami TBC dianggap sebagai orang yang mendapatkan kutukan, kotor, atau harus dikucilkan dan dijauhkan dari masyarakat. Dihubungkannya TBC dengan penilaian tertentu seperti itu mengakibatkan seseorang yang mengalami TBC dinilai secara tidak adil dan mereka sering dicirikan dengan kualitas dan perilaku yang tidak diinginkan oleh masyarakat umum.

Secara historis, TBC sering distigmatisasi karena sifatnya yang menular dan ketidaktahuan masyarakat tentang penyebab, cara penularan, atau pengobatannya. Stigma ini juga sering dikaitkan dengan kelompok yang kurang beruntung, misalnya mereka yang berada di bawah garis kemiskinan, tinggal di penjara, mengalami HIV/AIDS, atau sebagai migran.

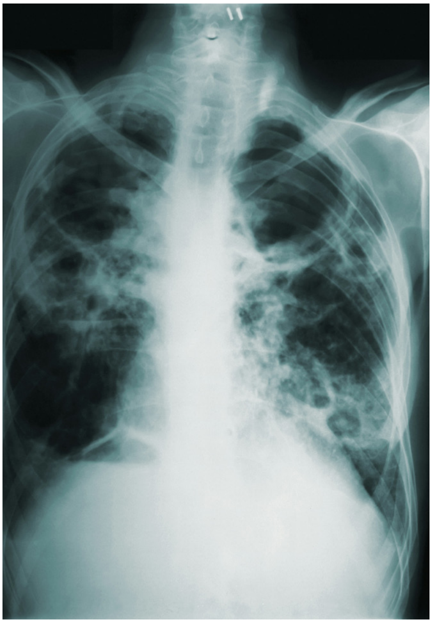

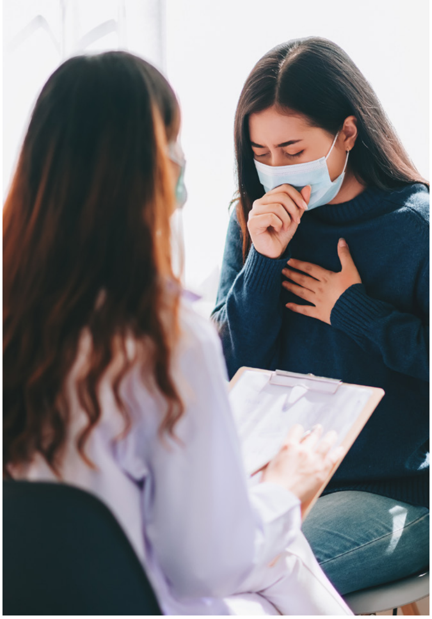

Sumber foto:
(i) Photo by CDC on Unsplash
(ii) Photo from Freepik

Bentuk Stigma

Ada beragam bentuk stigma yang dapat muncul baik di diri maupun terhadap orang yang mengalami TBC.

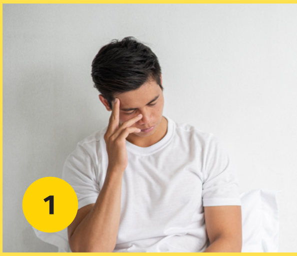

Stigma yang dialami
(Enacted atau Experienced Stigma)

Stigma ini berhubungan dengan perilaku yang langsung dirasakan oleh seseorang yang memiliki TBC. Misalnya, seseorang yang mengalami TBC dipandang sinis oleh tetangganya, dianggap buruk oleh keluarganya, atau dikucilkan di tempat kerjanya.

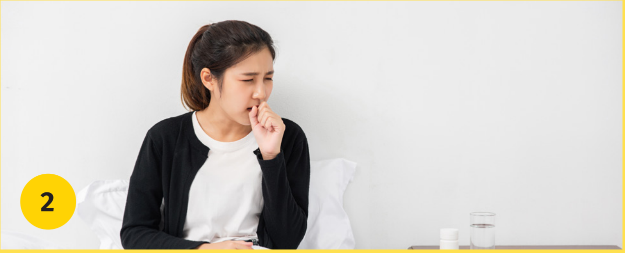

Stigma yang diantisipasi
(Anticipated Stigma)

Stigma ini berkaitan dengan kekhawatiran atau ketakutan diperlakukan berbeda karena orang lain mengetahui bahwa dirinya mengalami TBC. Misalnya, karena takut dikenali mengalami TBC, seseorang mungkin memutuskan untuk tidak pergi ke fasilitas kesehatan untuk memeriksakan keluhannya atau tidak mengambil obat TBC di fasilitas kesehatan secara teratur. Kekhawatiran seseorang kehilangan teman atau dijauhi orang lain ketika mereka tahu penyakitnya, juga termasuk dalam stigma ini.

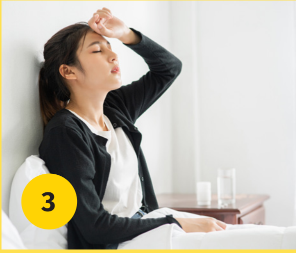

Stigma internal atau diri sendiri
(Internalized atau Self-Stigma)

Stigma ini terjadi ketika orang yang mengalami TBC menerima stereotip negatif dan mungkin bertindak sesuai dengan stereotip ini. Misalnya, seseorang yang merasa dirinya telah mempermalukan keluarga dan merasa bernilai rendah karena mengalami TBC.

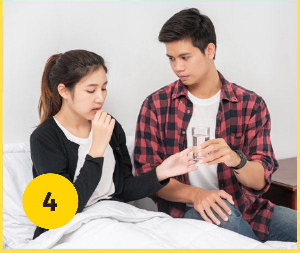

Stigma sekunder atau eksternal *(Secondary atau External Stigma).*

Stigma ini adalah sikap negatif dari masyarakat atau orang sekitar yang dialami oleh anggota keluarga, pengasuh, teman, atau pekerja kesehatan TBC karena mereka tinggal bersama atau memiliki kontak dekat dengan orang yang mengalami TBC. Misalnya, anak dari orang yang mengalami TBC juga dikucilkan dari masyarakat.

Sumber foto:
(i) (ii) (iii) (iv)
Photo from Freepik

Dampak negatif stigma TB

Karena takut dikenali sebagai orang yang mengalami TBC, seseorang mungkin saja memutuskan untuk tidak memeriksakan keluhannya yang terkait TBC ke fasilitas kesehatan. Mereka mungkin malu atau takut akan konsekuensi jika benar-benar didiagnosis TBC, misalnya khawatir dikucilkan oleh masyarakat atau dikeluarkan dari pekerjaannya. Hal ini sering mengakibatkan keterlambatan diagnosis dan keterlambatan memulai pengobatan. Selain itu, stigma TBC dapat membuat seseorang tidak patuh berobat. Karena mereka khawatir penyakitnya diketahui oleh tetangga atau teman mereka, atau dikeluarkan dari pekerjaan, mereka memilih tidak melanjutkan pengobatannya di fasilitas kesehatan. Pada akhirnya, TBC yang tidak didiagnosis dan ditangani dengan baik dapat menyebabkan penularan yang lebih luas di masyarakat.

Stigma TBC juga menyebabkan perasaan malu atau rendah diri terhadap identitasnya yang mengalami penyakit tersebut. Stigma TBC tersebut dapat memperburuk situasi yang mereka hadapi dalam mengatasi kondisinya, merusak hubungan sosial, menimbulkan beban keuangan, dan terlanggarnya hak dan martabat mereka yang terkena dampak dari stigma tersebut. Semua hal ini dapat meningkatkan tekanan atau stres dan dapat menimbulkan masalah psikologis dan sosial lainnya, seperti kecemasan dan depresi, serta menurunkan kualitas hidup. Studi Fuady dkk (2024) menunjukkan bahwa stigma TBC tersebut juga berkaitan dengan depresi dan menurunnya kualitas hidup orang yang mengalami TBC.

Stigma dan Dukungan Teman Sebaya
(Peer Support)

Stigma TBC, baik dalam bentuk antisipasi *(anticipated)* dan pengalaman *(experienced)*, masih tinggi di Indonesia. Oleh karena itu, orang yang mengalami TBC bukan hanya membutuhkan pengobatan terhadap penyakit fisiknya, tetapi juga membutuhkan dukungan sosial dari keluarga dan masyarakat sekitar, termasuk dari teman sebaya *(Peer)*. Teman sebaya dianggap dapat memberikan dukungan yang optimal karena sifat hubungan antar mereka tidak bertingkat *(hierarkikal)* sehingga orang dengan TBC dapat lebih mudah dan leluasa mengutarakan permasalahan yang dihadapinya.

Dukungan teman sebaya *(Peer Support)* saat ini masih dianggap kurang. Banyak orang dengan TBC yang membutuhkan Peer Support, tetapi tidak mendapatkan dukungan yang mereka harapkan tersebut. Salah satu persoalannya adalah belum adanya bentuk dukungan yang secara formal disediakan. Berbeda dengan dukungan dari tenaga kesehatan dan kader kesehatan, yang sudah tersedia secara formal, baik berupa konseling pribadi maupun kunjungan rumah, dukungan teman sebaya *(Peer Support)* lebih sering muncul secara natural (organik) di lingkungan mereka yang mengalami TBC.

Beberapa inisiatif sudah dilakukan untuk memberikan dukungan teman sebaya *(Peer Support)*, terutama bagi mereka yang mengalami TBC kebal obat. Bentuknya dapat berupa pertemuan berkelompok yang diselenggarakan secara rutin. Dalam pertemuan tersebut, mereka dapat berbagi pengalaman, keresahan, dan cara mengatasinya. Pendekatan ini dianggap efektif karena seseorang dapat menyampaikan perasaannya secara lebih terbuka dan mendapat umpan balik yang bermanfaat dari orang yang pernah mengalaminya.

Dukungan teman sebaya (Peer Support) saat ini masih dianggap kurang. Banyak orang dengan TBC yang membutuhkan Peer Support, tetapi tidak mendapatkan dukungan yang mereka harapkan.

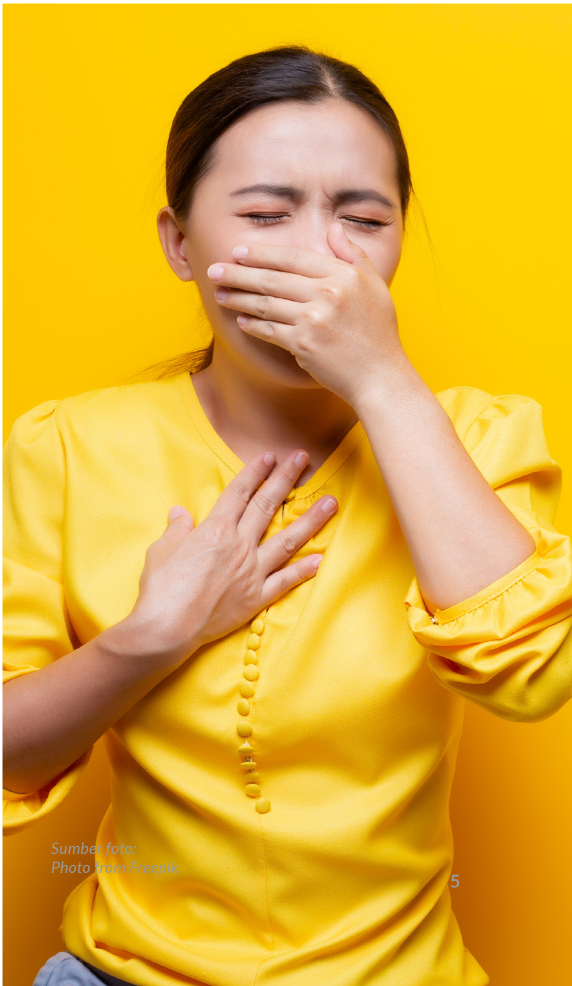

Sumber foto:
Photo from Freepik

02.

Workshop 2
Konsep Pendampingan Psikososial dan Dukungan Teman Sebaya *(Peer Support)*

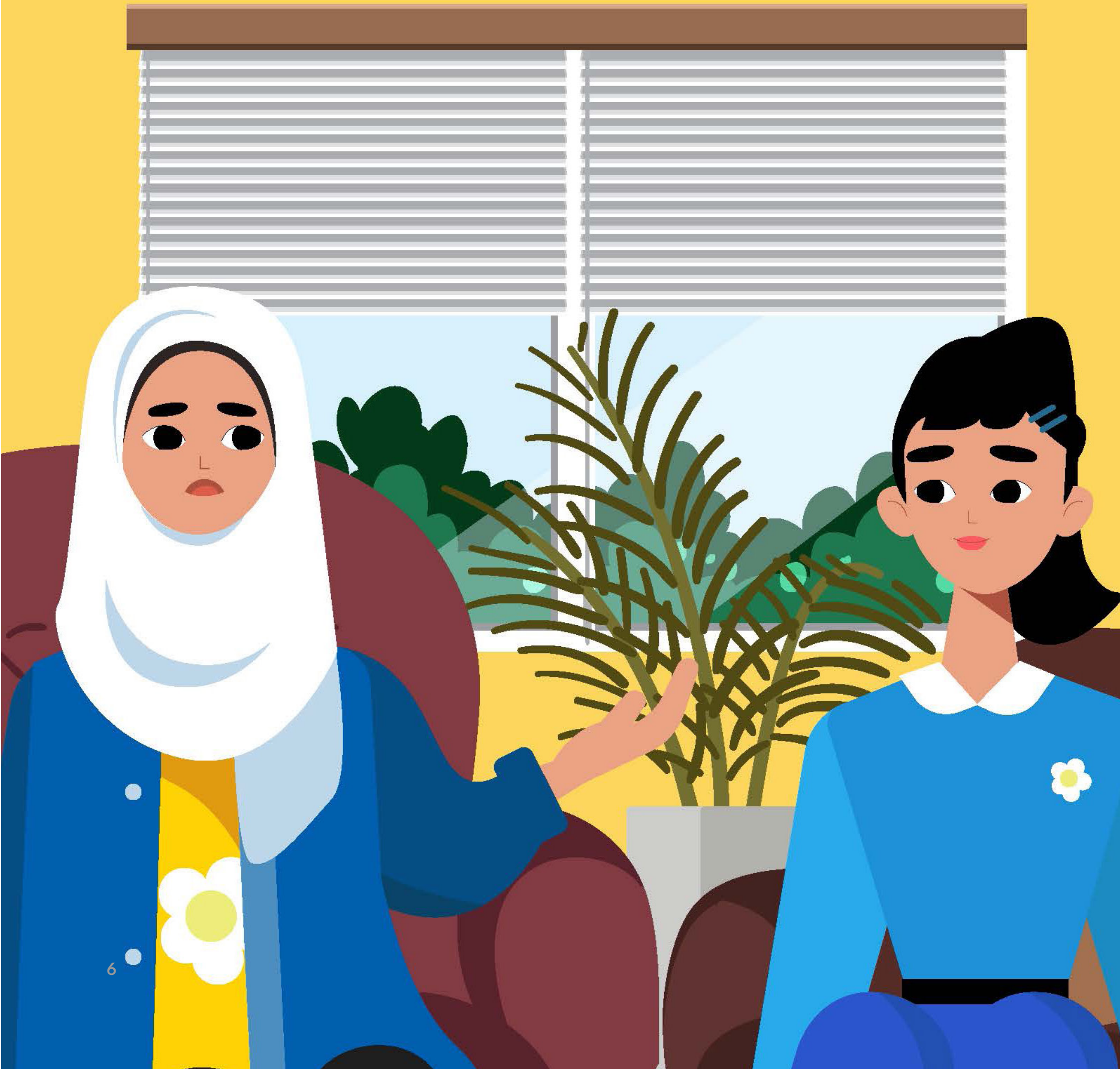

Pengenalan Konsep Pendampingan Psikososial

Seperti telah diurai sebelumnya bahwa orang dengan TBC berpotensi mengalami dampak psikososial, maka pendampingan psikososial penting untuk diberikan. Pendampingan psikososial ini bermanfaat dalam membantu dalam meningkatkan fungsi individu, baik sebagai perorangan atau anggota kelompok, sehingga mereka dapat melaksanakan tugas dan aktivitas kehidupannya, termasuk dalam menjalankan fungsi sosialnya.

Pendampingan psikososial pada dasarnya adalah memberikan dukungan terhadap aspek psikologis–yang berkaitan dengan pikiran, perasaan, nilai, dan kepercayaan masing-masing individu– dan aspek sosial yang berkaitan dengan hubungan individu dengan lingkungannya. Kedua aspek tersebut, yaitu psikologis dan sosial, memiliki hubungan yang dinamis dan saling berinteraksi secara berkelanjutan. Oleh karena itu, dukungan terhadap kedua aspek tersebut perlu diberikan secara utuh.

Tujuan Pendampingan Psikososial terhadap orang dengan TBC:

1. Membantu orang dengan TBC agar mereka mampu memahami persoalan yang mereka alami dari berbagai dimensi.
2. Membantu orang dengan TBC untuk mengambil keputusan yang terbaik bagi dirinya, dan keluarganya, dalam mengatasi masalah yang mereka hadapi saat ini.
3. Membantu orang dengan TBC untuk menemukan dan menggunakan potensi yang ada di dalam dirinya sendiri.
4. Membantu orang dengan TBC untuk mencegah terjadi masalah di masa depan dan menghadapinya.

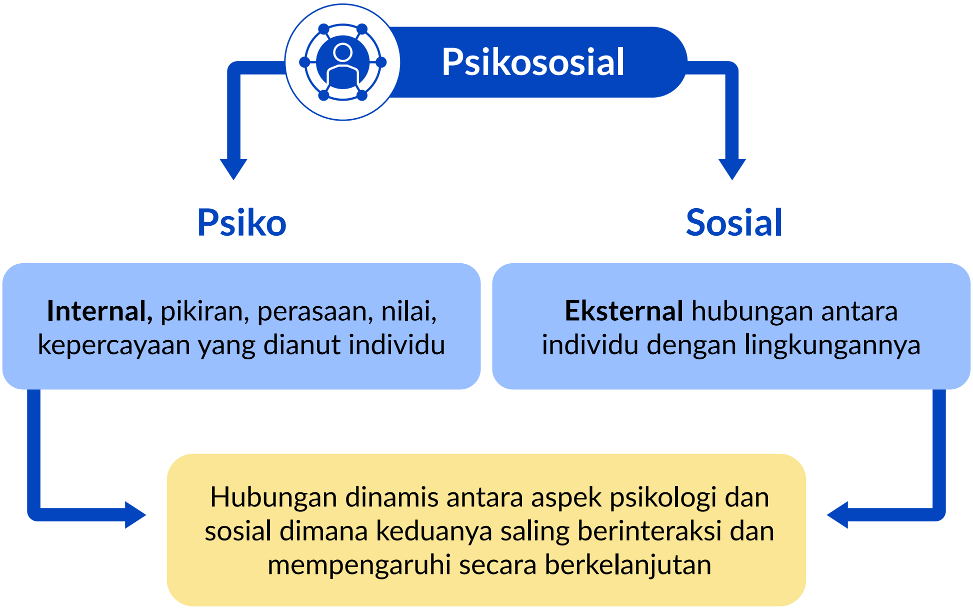

Gambar 1. Konsep Pendampingan Psikososial

Grafik diadaptasi dari materi pelatihan "Prinsip Dasar Pelaksanaan Pendampingan Psikososial", Ikatan Psikolog Klinis Indonesia

Jenis Dukungan Psikososial bagi orang dengan TBC

**Dukungan Informasi**	Memberikan penjelasan tentang situasi dan segala sesuatu yang berhubungan dengan masalah yang sedang dihadapi orang dengan TBC. Misalnya, pemberian informasi tentang pengobatan dan efek samping obat, siapa yang harus dikontak saat mengalami masalah medis, atau memberi tahu fasilitas kesehatan rujukan.
**Dukungan Emosional**	Memberikan pendampingan dengan empatik. Misalnya, mendengarkan curahan hati, bersikap terbuka, dan menunjukan sikap percaya terhadap apa yang dikeluhkan. Dukungan emosional akan membuat si penerima merasa berharga, nyaman, aman, terjamin, dan disayangi.
**Dukungan instrumental**	Memberikan bantuan yang bersifat fasilitas atau materi secara langsung. Misalnya, bantuan transportasi ke fasilitas kesehatan, memberikan makanan, atau bantuan fisik/program yang lain.
**Dukungan Nilai**	Memberikan dukungan yang berbentuk nilai, Misalnya, memberikan penilaian positif, penguatan (pembenaran) untuk melakukan sesuatu, umpan balik, atau menunjukan perbandingan sosial yang membuka wawasan seseorang.

Konsep Dukungan Teman Sebaya *(Peer Support)*

Apa itu Teman Sebaya *(Peer)*?

Teman sebaya *(Peer)* didefinisikan sebagai individu yang memiliki karakteristik yang sama. Individu-individu tersebut biasanya berbagi nilai-nilai, pengalaman, dan gaya hidup yang serupa, serta biasanya berada dalam kelompok usia yang sama.

Apa itu Dukungan Teman Sebaya *(Peer Support)*?

Dukungan Teman Sebaya umumnya diartikan sebagai bentuk dukungan yang diberikan oleh seseorang yang memiliki pengalaman hidup yang sama dengan orang yang mengalami hal yang sama, termasuk penyakit yang sama. Mereka dianggap memiliki kapasitas untuk membantu satu sama lain berdasarkan kesamaan afiliasi dan pemahamannya yang mendalam tentang pengalaman serupa. (SAMHSA, 2015).

Dukungan Teman Sebaya dapat berupa bantuan yang diberikan antar individu, dukungan melalui diskusi berkelompok, pemberian nasihat, *tutoring*, dan semua aktivitas yang melibatkan bantuan secara interpersonal.

Peran Pendukung Sebaya *(Peer Supporter)?*

Peran Pendukung Sebaya adalah memberikan dukungan psikososial dalam bentuk dukungan informasi, emosional, instrumental, maupun nilai, seperti yang telah dijelaskan sebelumnya. Dukungan tersebut dapat berupa mendengarkan dengan empatik, memberi informasi yang dibutuhkan, memberi nasihat ketika diminta, dan menyarankan rujukan ketika diperlukan.

Selain dilakukan dalam *setting* antar individu, Pendukung Sebaya juga dapat memberikan dukungan psikososail tersebut dalam kelompok. Di dalam kelompok, Pendukung Sebaya yang terlatih berperan menjadi asisten atau pemimpin dalam diskusi kelompok pendukung serta dapat membantu melatih kelompok pendukung sebaya yang baru.

Mengapa melakukan Dukungan Teman Sebaya *(Peer Support)?*

Program dukungan teman sebaya dapat membantu orang dengan TBC dalam mendapatkan dukungan informasi terkait penyakit dan dukungan psikososial, membantu orang dengan TBC untuk mengurangi perasaan dikucilkan (isolasi) secara sosial, dan menghubungkan mereka dan orang-orang yang merawatnya dengan orang lain yang memiliki masalah kesehatan serupa. (Hui Joo, 2022)

Teman sebaya juga dapat membantu orang dengan TBC dengan berperan sebagai pendengar ketika bantuan profesional tidak tersedia, tidak dapat diakses, atau dianggap belum bermanfaat.

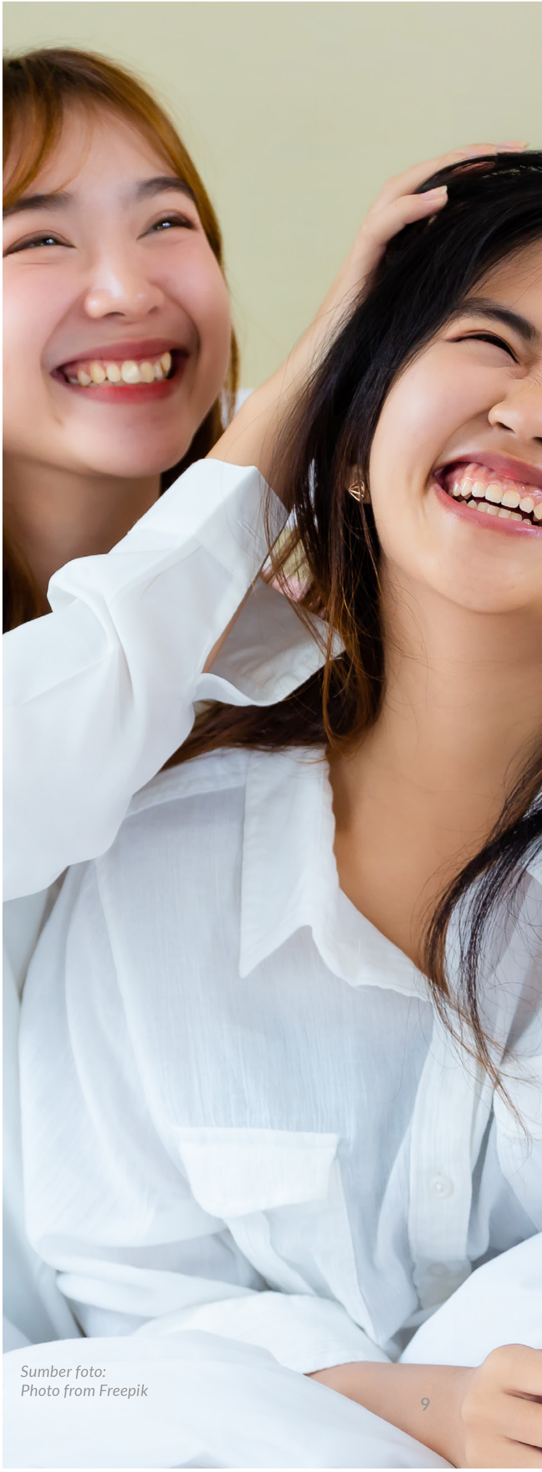

Sumber foto:
Photo from Freepik

Tiga Sifat Penting Menjadi Pendukung Sebaya *(Peer Support)*

Sifat Penting Yang Digunakan Dalam Keterampilan Membantu

Diadaptasi dari Brown (1998).

Menerima *(acceptance)*	Ketulusan *(genuineness)*	Empati
• **Menerima** mereka apa adanya<br>• **Menghormati** keyakinan individu<br>• **Menghargai** mereka dan pandangan mereka<br>• Bersikap **tidak menghakimi**<br>• **Mendengarkan** secara aktif<br>• Bersikap penuh **perhatian**<br>• Menjaga **kerahasiaan**	• Menjadi diri sendiri<br>• Terus terang<br>• Tulus<br>• Jujur<br>• Terbuka dan jelas	• **Peduli** untuk memahami apa yang sebenarnya "terjadi" pada seseorang<br>• **Mengajukan pertanyaan yang relevan** tentang keadaan seseorang<br>• **Berpikir, bertindak dan merasakan** kepentingan orang tersebut<br>• **Menahan diri** dari membuat asumsi atau penilaian

Saat membantu orang lain, perlu menunjukkan bahwa dalam membantu mereka, Anda menerima dan menghormati mereka apa adanya. Mereka perlu merasa aman untuk berbagi dan mengeksplorasi masalah tanpa ancaman rasa malu atau cemoohan.

Untuk membantu orang lain secara efektif, Anda harus tulus sebagai pribadi dan sebagai pendukung sebaya. Menunjukkan pribadi yang tulus dapat membangun landasan kepercayaan untuk hubungan dalam saling membantu.

Definisi sederhana dari empati adalah kemampuan untuk menempatkan diri pada posisi orang lain. Orang sering tertukar antara empati dengan simpati. Empati bukan bersimpati atau merasa kasihan terhadap orang tersebut, atau terlibat secara emosional atau membuat asumsi. Melainkan tentang melihat sesuatu dari sudut pandang orang lain, berusaha keras untuk memahami makna suatu hal dari orang lain dan melihat dunia sebagaimana orang lain melihatnya.

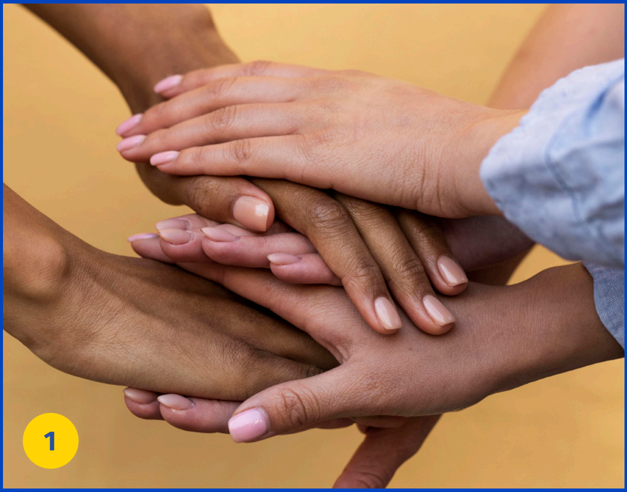

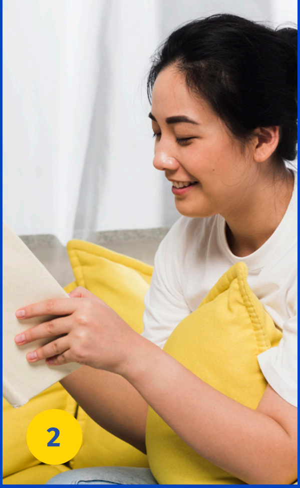

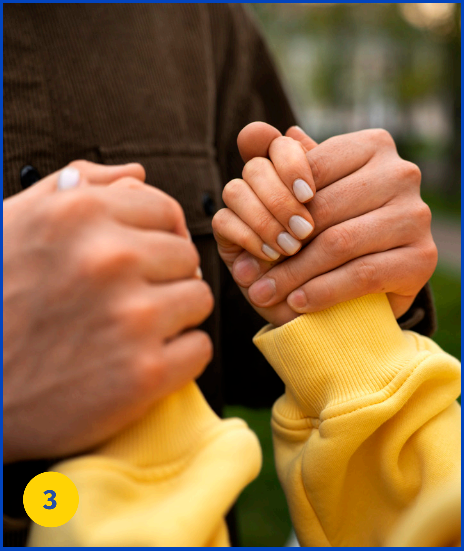

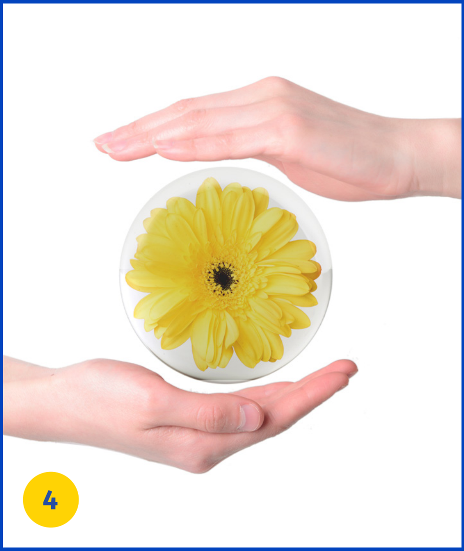

Sumber foto:
Photo from Freepik

Bentuk Bantuan Teman Sebaya dalam Support Group

1 Memberikan dukungan

Teman sebaya dapat memfasilitasi atau menjadi bagian dari kelompok yang saling memberikan dukungan antara anggota dalam kelompok. Ini dibutuhkan supaya masing-masing anggota tidak merasa sendirian dalam menghadapi masalahnya. Setiap anggota kelompok memiliki kesempatan yang besar dan sama dalam berinteraksi satu sama lain.

2 Mengkomunikasikan pengetahuan sesuai dengan pengalaman yang dimiliki

Teman sebaya membantu agar setiap anggota kelompok bisa saling bertukar informasi ketika berinteraksi dalam kelompok. Ini akan mendukung kelengkapan informasi yang diperoleh ketika mengikuti sesi.

3 Mendampingi dalam proses tumbuhnya rasa saling memiliki *(sense of belonging)*

Untuk menumbuhkan rasa saling memiliki, Teman Sebaya dapat mengawalinya dengan proses pertukaran informasi, termasuk kondisi yang dialami masing-masing anggota kelompok. Hal ini dapat menumbuhkan rasa senasib sepenanggungan akibat masalah yang dihadapi sehingga dapat menggerakkan setiap anggota untuk saling membantu.

4 Mengajarkan metode *coping*

Teman Sebaya membantu memfasilitasi anggotanya untuk saling berbagi pengetahuan, dukungan, maupun pengalaman ketika membahas suatu masalah. Pengalaman dan dukungan tersebut dapat membantu anggota kelompok menemukan cara efektif dalam mengatasi masalah *(coping)*.

Bantuan yang diberikan melalui dukungan teman sebaya *(Peer Support)*

Jenis Dukungan	Contoh dukungan psikososial
Dukungan Informasi	• Memberikan informasi tentang pengobatan dan pengalaman efek samping obat<br>• Memberi saran atau nasihat, ketika diminta<br>• Memberi tahu siapa yang harus dikontak saat mengalami masalah medis<br>• Memberi tahu fasilitas kesehatan rujukan atau orang yang dapat menjawab pertanyaannya dengan lebih baik
Dukungan Emosional	• Berbicara dengan rekan-rekan orang dengan TBC terkait masalah yang dialami penyakitnya<br>• Mendengarkan curahan hati atau keluhan mereka<br>• Mengeksplorasi solusi untuk beberapa masalah
Dukungan instrumental	Mendampingi pergi ke fasilitas kesehatan untuk berobat
Dukungan Nilai	• Menguatkan sikap dan keputusan untuk melakukan sesuatu, yang dapat membantu pengobatannya<br>• Memberikan umpan balik terhadap nilai yang disampaikan ketika berbicara

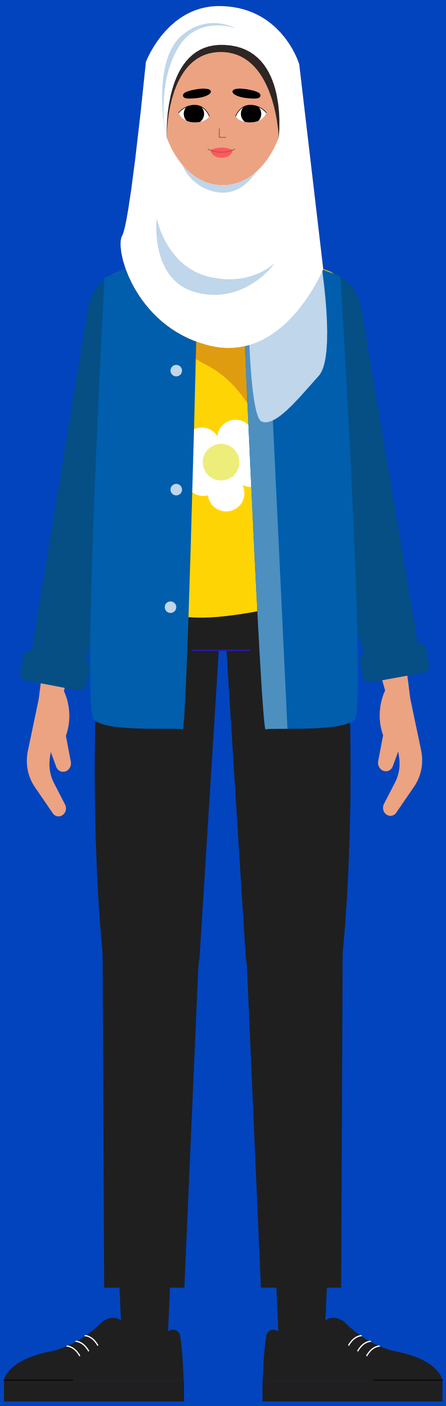

Teman sebaya (Peer) didefinisikan sebagai individu yang memiliki karakteristik yang sama. Individu-individu tersebut biasanya berbagi nilai-nilai, pengalaman, dan gaya hidup yang serupa, serta biasanya berada dalam kelompok usia yang sama.

03.

Workshop 3
Mendengarkan Aktif, Mendengarkan yang Efektif, Teknik Komunikasi

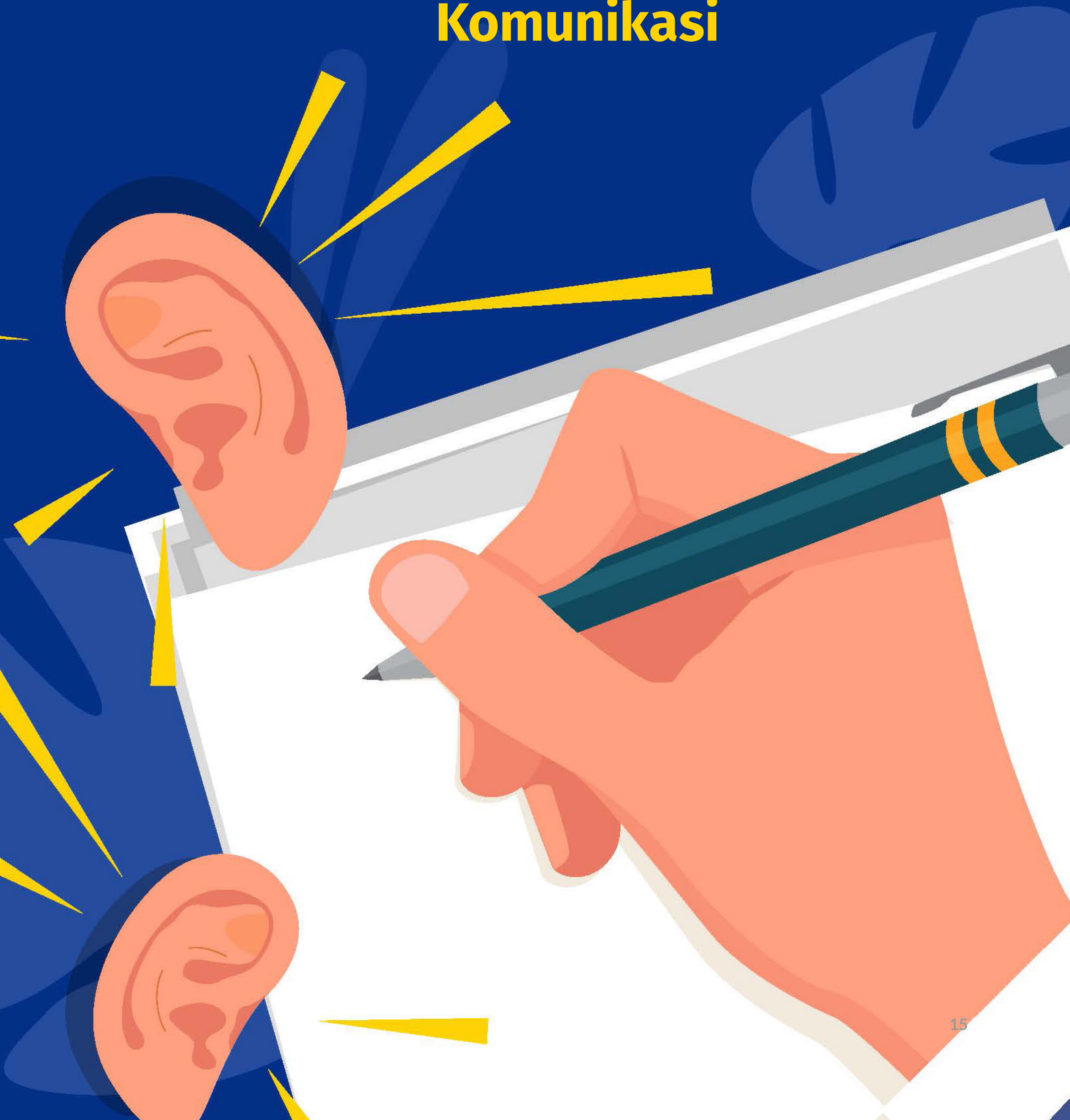

Kunci Mendengarkan Aktif

"Kunci utama dalam mendengarkan aktif adalah ketulusan dan keinginan membantu orang lain."

Dalam mendengarkan aktif, kunci utamanya adalah mempersiapkan ketulusan hati dan keinginan untuk membantu orang lain. Jika kita sudah mempersiapkan dan menunjukkan ketulusan dalam mendengarkan, secara otomatis kita akan mampu mendengarkan mereka secara aktif.

Berikut ini beberapa hal detail yang dapat membantu dalam proses mendengarkan aktif

1. **Biarkan mereka berbicara – Jangan mendominasi pembicaraan saat berbicara dengan lawan bicara.** Kita tidak dapat mendengarkan lawan bicara dengan baik jika kita terus berbicara. Fokuskan perhatian kita pada kata-kata, pemikiran, dan perasaan lawan bicara kita.

2. **Jangan terlalu cepat merespons** – Jangan menginterupsi lawan bicara kita dan berikan mereka waktu dan kesempatan untuk mengatakan hal yang ingin mereka sampaikan.

3. **Lihat lawan bicara kita** – Lihat wajah, mulut, mata, dan tangan mereka, bahasa tubuh yang membantu mereka dalam berkomunikasi dengan kita. Hal ini juga dapat membantu kita dalam berkonsentrasi. Buat mereka merasa bahwa kita mendengarkan mereka.

4. **Menyingkirkan distraksi** – Siapkan diri dan tempat kita sebelum berbicara. Letakkan handphone, komputer, atau apapun yang dapat mengalihkan perhatian kita jauh dari jangkauan kita selama berbicara.

5. **Bereaksi terhadap idenya, bukan pada orangnya** – Fokuskan diri kita pada apa yang dibicarakan. Jangan biarkan perasaan kita terhadap lawan bicara, misalnya kita tidak suka pada orang tersebut, mempengaruhi bagaimana kita merespons dan menginterpretasi pembicaraannya. Ide mereka bisa saja sangat baik meskipun kita tidak menyukai orang tersebut.

6. **Memperhatikan emosinya saat berbicara** – Saat berbicara, perhatikan emosi dan perilaku lawan bicaranya, misalnya air matanya menggenang, bibirnya bergetar, atau kakinya terus bergerak-gerak. Hal itu dapat menguatkan pemahaman dan fokus kita pada isi pembicaraannya.

7. **Memberikan waktu dan kesempatan untuk berpikir** – Seringkali pembicaraan terhenti, misalnya mereka tidak mampu melanjutkan kata-katanya karena emosional atau tidak menjawab karena sungkan. Cobalah untuk membiarkan kesunyian tersebut, dan berikan lawan bicara waktu untuk memikirkan hal yang akan mereka sampaikan. Jika kita memiliki keterbatasan waktu (misalnya karena kita memiliki agenda lain), sampaikan batasan waktu sejak awal kepada lawan bicara kita.

8. **Menghindari asumsi** – Asumsi dapat menghalangi kita dalam memahami orang lain. Jangan berasumsi bahwa mereka:
    a. Menggunakan kata-kata sama seperti yang kita gunakan
    b. Merasakan sama seperti yang kita rasakan
    c. Memutarbalikan fakta, karena yang mereka katakan tidak sesuai dengan yang kita pikirkan

d. Berbohong, karena mereka menginterpretasikan fakta berbeda dengan kita

e. Tidak etis, karena berusaha membujuk kita agar setuju dengan mereka

f. Marah, karena mereka tidak antusias dalam menyampaikan pandangan mereka Asumsi seperti ini bisa jadi benar, tetapi lebih sering menjadi penghalang bagi kita untuk memahami dan berkompromi/ mencapai kesepakatan dengan lawan bicara.

9. **Menahan keinginan untuk memecahkan masalah** – Kita hadir untuk mendengarkan mereka, bukan memecahkan masalah yang mereka hadapi. Jangan berfokus pada mencari jawaban/ solusi, dan baru berikan saran jika memang diminta oleh mereka.

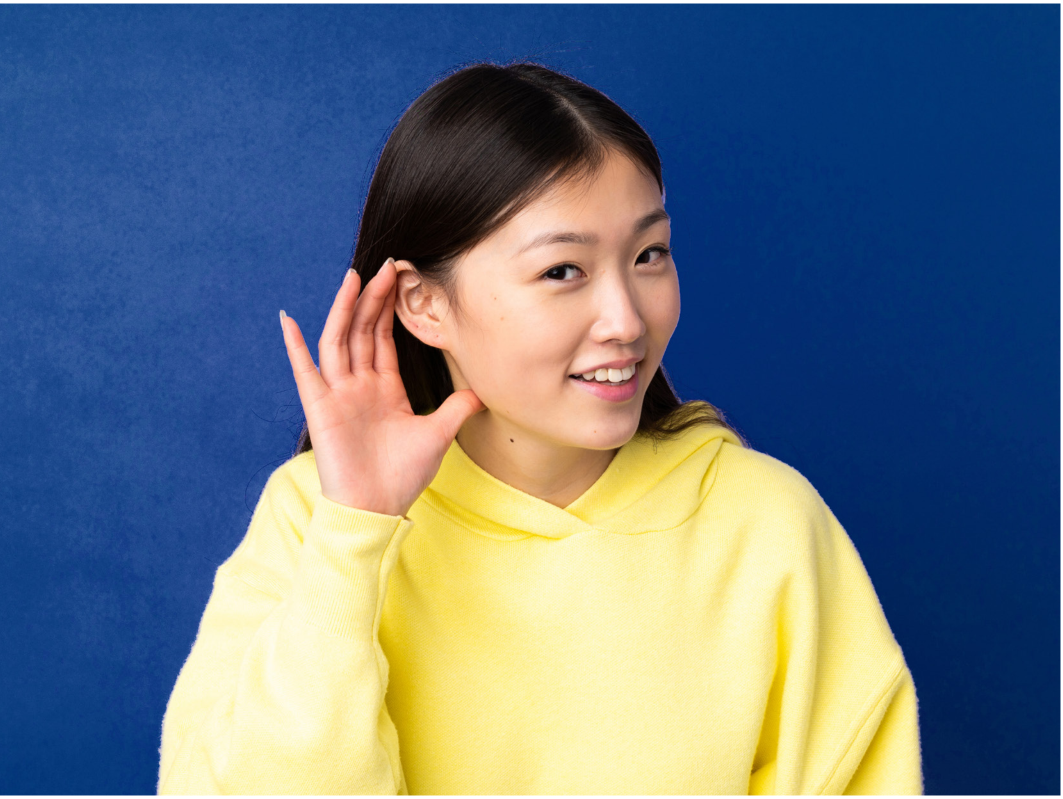

Sumber foto:
Photo from Freepik

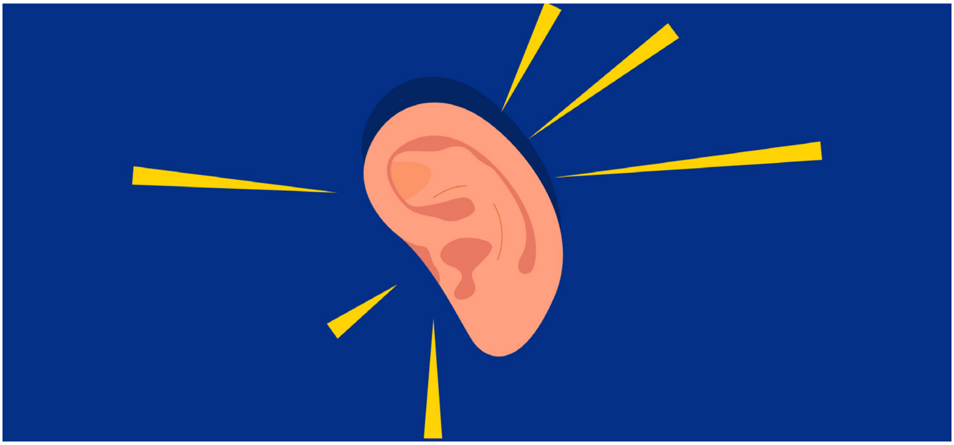

Mendengarkan Efektif

Menurut Egan (1994), terdapat 2 aspek penting dalam percakapan:

1. **Pesan verbal**
2. **Pesan non-verbal**

Penting untuk mendengar dan memahami pesan inti dari seseorang, kemudian mengkomunikasikan pemahaman tersebut dengan baik. Terdapat 3 bagian dalam pesan verbal:

1. Pengalaman – hal yang telah terjadi dan dialami oleh mereka
2. Perilaku – hal yang mereka putuskan untuk lakukan atau tidak lakukan
3. Perasaan – emosi yang muncul

Situasi yang problematik dapat menjadi jelas jika dijabarkan dalam 3 aspek tersebut. Oleh karena itu, ketika sebaya Anda berbicara, cobalah untuk tidak memikirkan bagaimana meresponsnya. Dengarkan dengan baik dan identifikasi ketiga bagian pesan verbal. Jika seseorang hanya berbicara tentang pengalamannya, sebagai sebaya Anda mungkin ingin bertanya tentang perilaku atau perasaannya. Mengeksplorasi ketiga bagian pesan verbal dapat memperjelas situasi.

Saat mendengarkan, ada baiknya untuk tetap mengikuti apa yang disampaikan. Untuk itu, ingat pertanyaan-pertanyaan ini:

- Apa tema inti dalam pembicaraan?
- Bagaimana sudut pandang orang tersebut?
- Apa hal paling penting bagi orang tersebut?
- Apa yang lawan bicara harapkan untuk Anda mengerti?

Mendengarkan adalah suatu proses yang aktif. Untuk menjadi seorang pendengar yang efektif, penting untuk mengklarifikasi bahwa makna yang kita tangkap sesuai. Memahami situasi seseorang dengan baik dan mengkomunikasikannya kembali kepada orang tersebut akan meningkatkan empati dalam percakapan.

Berikutnya adalah komunikasi non-verbal, dimana kita membaca petunjuk implisit dan perilaku seseorang.

Sangat penting untuk mengenali pesan-pesan non-verbal tersebut tanpa menggunakannya secara berlebihan maupun kekurangan. Perilaku non-verbal dapat mempengaruhi (misalnya menyangkal, menguatkan, atau merancukan). Dengan latihan dan pengalaman, kita dapat membaca dan mengartikannya dalam situasi apapun.

Selain memahami bahasa tubuh lawan bicara, kita juga harus menyadari bahasa tubuh kita. Dalam hubungan yang membantu, bisa saja kita mengubah pesan verbal yang kita sampaikan dengan perilaku dan pesan non-verbal.

Indikator Non-Verbal Penting:

- **Gerak-gerik tubuh,** seperti postur, gerakan tubuh, gestur
- **Ekspresi wajah,** seperti senyuman, kerutan dahi, mengangkat alis mata, bibir yang dimiringkan, atau menyeringai
- **Perilaku yang berhubungan dengan suara,** seperti suara, nada, kekerasan suara, intensitas
- **Respons otonomi fisiologis** yang dapat dilihat, seperti napas yang cepat, ruam, kemerahan, pucat, atau dilatasi pupil yang bersifat sementara
- **Ciri fisik,** seperti kebugaran, tinggi, berat, dan rona wajah
- **Penampilan umum,** seperti dandanan dan pakaian

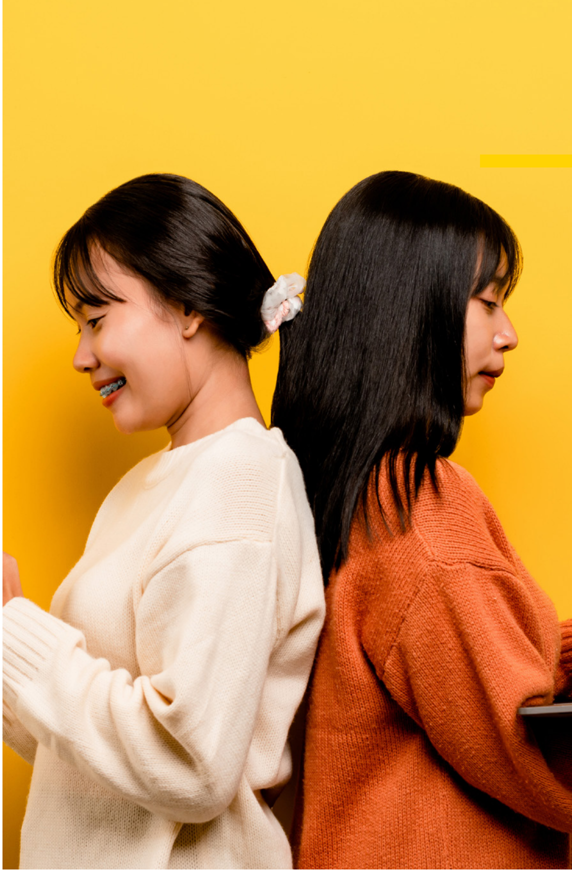

Teknik Komunikasi

Merefleksikan & Memparafrase

Refleksi merupakan alat yang sangat penting yang digunakan untuk mencerminkan makna dan perasaan dari apa yang dikatakan seseorang (Gilliland & James, 1993, Moursund, 1990). Biasanya **merefleksikan isi percakapan dilakukan dengan menyampaikan kembali isi percakapan yang lawan bicara sampaikan.** Hal ini bertujuan untuk mengkomunikasikan pemahaman tentang sudut pandang pembicara. Terdapat dua tipe dalam refleksi pembicaraan.

Refleksi isi :

Merefleksikan isi pembicaraan dengan menyampaikan kembali poin-poin penting yang disampaikan oleh lawan bicara dengan kata-kata kita sendiri

Contoh

"Saya dan orang tua saya seringkali bertengkar akibat penyakit TB saya yang dianggap mengurangi pemasukan keuangan keluarga"

Anda dapat merefleksikan isi dengan mengatakan:

"Jadi anda dan orang tua anda sering berdebat mengenai biaya yang dikeluarkan akibat penyakit TB anda"

Refleksi perasaan :

Merefleksikan emosi atau perasaan yang diungkapkan langsung atau ditampilkan secara nonverbal dari lawan bicara

Contoh

"Semenjak saya didiagnosis dengan penyakit TB, hubungan saya dan suami menjadi merenggang" (mata lawan bicara terlihat berair)

Anda dapat merefleksikan isi dengan mengatakan:

"Saya melihat anda merasa sedih karena hubungan dengan suami anda yang tidak lagi dekat"

Memparafrase adalah kemampuan untuk menyatakan kembali apa yang baru saja dikatakan oleh lawan bicara dengan kalimat sesuai pemahaman anda (Gilliland & James, 1993, Moursund, 1990). Dalam percakapan, pendengar mengulang pesan dengan kata-kata mereka sendiri, atau bisa menggunakan ilustrasi konkret, contoh kejadian, atau perumpamaan untuk menyampaikan pemahaman mereka terhadap isi pembicaraan. Memparafrase berarti menggabungkan pikiran, perasaan, dan/atau tindakan untuk memberikan gambaran yang lebih jelas bagi kedua orang yang terlibat dalam percakapan. Memparafrase juga memungkinkan pembicara untuk mendengar kembali pernyataan yang mereka buat.

Terakhir, memparafrase tidak hanya sekedar mencerminkan apa yang dikatakan orang lain, melainkan juga mengundang orang tersebut untuk menggali lebih dalam atau memahami lebih baik apa yang sedang dibahas.

Contoh

Seorang dengan TBC menggambarkan pertengkaran sengit antara dirinya dan ayahnya mengenai penolakan ayahnya untuk meminjamkan uang kepadanya untuk pengobatan TBC. Saat dia berbicara tentang kemarahannya, matanya berkaca-kaca.

Kemungkinan parafrase

“Kamu marah pada ayahmu karena tidak memberimu pinjaman, dan pada saat yang sama kamu merasa kesal.”

Parafrase ini memperhitungkan isi (ayah tidak mau meminjamkan uang), perasaan yang diungkapkan secara verbal (marah), dan perilaku nonverbal (air mata).

Pedoman dalam memparafrase:

- **Bersikaplah tentatif** dan **sampaikan kesan Anda** terhadap apa yang dikatakan orang lain.
- **Hindari sikap memberi tahu** atau **mendefinisikan persepsi** orang lain.
- **Bersikap hormat:** jangan menghakimi, mengabaikan, atau menggunakan sarkasme.
- **Gunakan kata-katamu sendiri.** Mengulang persis apa yang dikatakan pembicara (parroting) bukanlah parafrase.
- Cobalah untuk **menyesuaikan diri dengan bahasa orang lain.** Gunakan kata-kata yang tepat dan tidak menyinggung ketika menggambarkan suatu peristiwa atau situasi, misalnya. “terluka” bukan “cacat”.
- **Dengarkan seberapa dalam perasaan yang diungkapkan** dalam suara orang tersebut dan renungkan tanggapan Anda sesuai dengan itu.
- **Jangan menambah apa yang dikatakan** orang tersebut, dan hindari interpretasi dan evaluasi.
- **Bersikaplah tulus** dan **jangan berpura-pura memahami** jika Anda tidak memahaminya.
- Sampaikan parafrase **secara singkat dan langsung.**

Agar parafrase menjadi efektif, kalimat yang disampaikan harus akurat. Meskipun demikian, parafrase juga tetap dapat dilakukan ketika anda tidak yakin apakah pemahaman anda akan isi percakapan sudah benar. Dengan melakukan parafrase, anda dapat mengkonfirmasi pemahaman anda apakah sudah tepat dan memperbaiki pemahaman tersebut.

Beberapa kemungkinan kalimat pengantar untuk parafrase:

- Jadi, apa yang Anda katakan adalah...
- Saya mendengar Anda mengatakan...
- Jika saya memahaminya dengan benar, Anda...
- Anda mengatakan kepada saya bahwa...
- Sepertinya Anda...

BUKAN:

- Anda tidak mengatakannya dengan jelas, ...
- Anda belum mengungkapkan hal itu dengan baik, ...

Contoh Dalam Parafrase

1. Saya benar-benar tidak bisa melamar pekerjaan itu. Bukannya saya tidak bisa melakukannya, saya hanya tidak tahan memikirkan pada saat diwawancara itu.

   Parafrase tanggapan: Anda merasa mampu melakukan pekerjaan tersebut, namun tidak dapat melamar karena merasa tidak mampu melakukan wawancara.

2. Saya sedang bingung. Saya tidak tahu apakah harus pindah ke pekerjaan lain atau tetap dalam pekerjaan yang sekarang.

   Parafrase tanggapan: Saya mendengar Anda bahwa Anda tidak yakin apakah Anda ingin berganti pekerjaan.

Merangkum

Merangkum adalah mengumpulkan poin-poin utama dari suatu diskusi dan mengaturnya sehingga dapat ditinjau, dikonfirmasi atau dikoreksi (Moursund, 1990). Merangkum sebuah pembicaraan dapat memiliki berbagai tujuan, termasuk untuk:

- **Memprioritaskan dan memfokuskan** pikiran dan perasaan
- **Menutup diskusi** terkait tema tertentu
- **Memulai diskusi yang lebih lanjut** atau ke tema selanjutnya
- **Memeriksa pemahaman** tentang percakapan yang berjalan
- **Mengeksplorasi cepat** suatu ide secara menyeluruh
- **Memfokuskan pembicaraan** yang mulai berlarut dan tidak bertujuan
- Mempertimbangkan cara-cara untuk **memajukan pembicaraan**

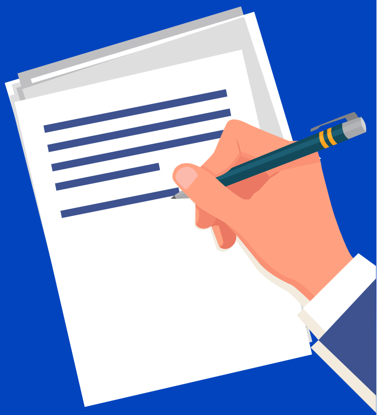

Pedoman dalam merangkum:

- **Menyatukan ide-ide utama dan perasaan** yang muncul dalam percakapan ke dalam satu pernyataan luas mengenai percakapan tersebut
- **Perhatikan tema percakapan dan emosi** dari pembicara
- Merangkum pembicaraan secara **singkat dan langsung**
- **Jangan menambah** apa yang dikatakan orang tersebut, dan **hindari asumsi**

Contoh

Dalam tanggapan berikut, pendengar merangkum apa yang dikatakan rekannya dan menambahkan pertanyaan untuk membantu percakapan terus berproses (Moursund, 1990).

Bolehkah saya memeriksa apakah saya memahami maksud anda dengan benar? Anda memberitahu saya tentang beberapa pilihan yang dapat anda jalani. Pilihan Anda untuk mengatasi hilangnya motivasi dalam berobat anda adalah dengan mengatasinya sendiri, membuat janji bertemu dengan psikolog, atau mendaftar dalam kelompok konseling. Namun, Anda merasa tidak ada satu pun pilihan yang terasa seperti solusi sempurna. Menurut Anda, apakah kelebihan dan kekurangan dari masing-masing kemungkinan ini?

Gangguan Dalam Berkomunikasi

Ketika sedang berlangsung sebuah percakapan dengan topik yang berulang dan kita sebagai pendengar, ada kemungkinan akan memicu gangguan komunikasi karena kita terjebak dalam respon yang kurang sesuai. Beberapa jenis hambatan dalam mendengarkan adalah:

a. Ketika seorang anggota kelompok sedang menyampaikan sesuatu, ada potensi muncul di benak kita keinginan untuk **membandingkan** situasi kita dengan dia. Misalnya: *"ah dulu saya lebih menderita lagi dibanding dia sekarang"*

b. Ketika seorang anggota kelompok sedang berbicara, maka kita berpotensi terpancing untuk **membaca pikiran** seperti dalam benak kita dapat muncul beragam asumsi yang kita paksa kaitkan dengan situasi lawan bicara kita. Misalnya: "Dia sering mengeluhkan perlakuan dokter, padahal saya lihat dokter itu baik, mungkin dia saja yang memang sentimen dengan dokter itu"

c. Saat seseorang sedang berbicara, maka kita berpotensi untuk **mengarang cerita** dengan muncul di benak kita rangkaian skenario kejadian atau pembicaraan yang akan terjadi sehingga kita tidak lagi memperhatikan konten yang disampaikan dia. Misalnya: "Kalau saya merespon begini, dia pasti akan menyangkalnya"

d. Saat seseorang sedang bercerita, kita berpotensi **memilah-milah informasi** mana yang ingin kita dengar dan yang tidak ingin kita dengar karena kita sudah tidak tertarik

e. **Sejak awal kita sudah memiliki penilaian** mengenai seseorang yang sedang berbicara dengan kita. Misalnya: kita menilai dia terlalu manja, sehingga hal-hal yang disampaikan akan kita nilai sebagai hasil dari sikap yang terlalu manja tersebut.

f. Ketika seseorang sedang berbicara mengenai sesuatu yang mengingatkan kita dengan hal atau kejadian serupa yang kita alami, kita akan berpotensi untuk **tidak fokus dan berkhayal** sehingga kita tidak memperhatikan dia

g. Saat seorang peserta yang sedang bercerita hal yang menarik, kita terpancing untuk **memotong pembicaraan** dan **mengaitkan dengan pengalaman pribadi** pada situasi yang sedang dia ceritakan. Selain itu, kita berpotensi untuk **terlalu cepat memberikan beragam saran dan solusi** yang kita anggap kita miliki. Sehingga pembicaraan akan terambil alih padahal dia belum selesai bercerita dan belum tentu saran tersebut diinginkan oleh dia.

h. Ketika seseorang menyampaikan pendapat atau bercerita, kita menyatakan ketidaksetujuan kita pada pemikirannya dan **mendebat menggunakan kata-kata yang merendahkan** dia. Misalnya: ketika seseorang bercerita tentang kesulitannya untuk mendapat penghasilan karena kondisi penyakitnya, kita akan mengatakan "kamu saja yang kurang berusaha, makanya dari dulu belajar teknologi supaya bisa berjualan di toko online"

i. Kita berpotensi untuk memiliki sikap **merasa paling benar** sehingga kita tidak bisa menerima kritik atau koreksi dari orang lain untuk berefleksi dan menyesuaikan diri.

j. Pada sebuah percakapan yang sedang terjadi, kita berpotensi untuk merasa bosan sehingga **kita berusaha mengganti topik** atau melontarkan lelucon untuk menghilangkan rasa bosan itu

k. Kita berpotensi ingin membuat orang lain senang sehingga secara sadar maupun tidak, kita **cenderung menyetujui semua yang disampaikan oleh dia** meskipun kita menyadari bahwa tidak semuanya bernilai baik. Kita cenderung **tidak berusaha untuk memikirkan kebenaran ide yang disampaikannya.**

04. Workshop 4 Pertanyaan Tertutup dan Terbuka

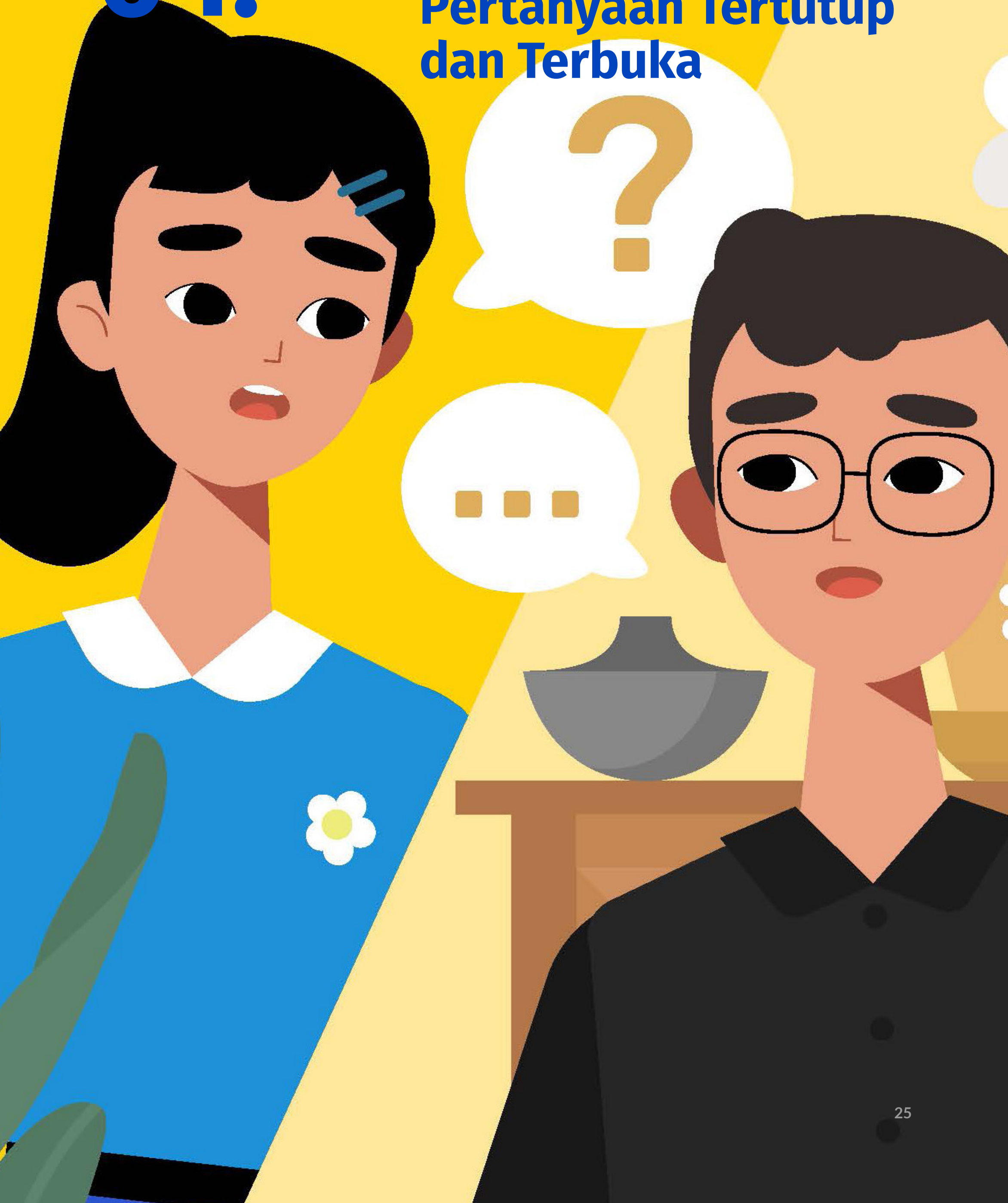

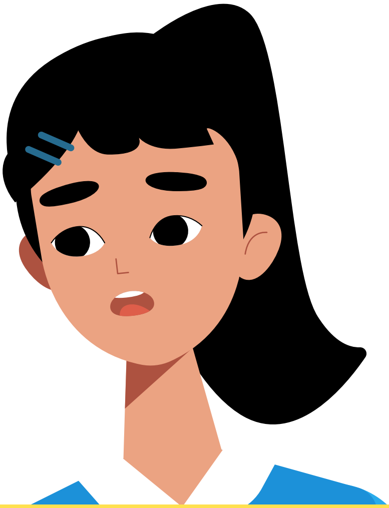

Dalam pendampingan Teman Sebaya *(Peer Support)* dan memfasilitasi pertemuan kelompok, keterampilan mengemukakan pertanyaan merupakan hal penting yang harus dikuasai. Ketepatan dalam bertanya akan membantu proses pendampingan. Sebaliknya, kesalahan dalam mengungkapkan pertanyaan dapat menghalangi proses pendampingan, bahkan mempersulit komunikasi. Untuk itu, pertanyaan harus dapat digunakan dengan tepat untuk dapat membantu pembicaraan berjalan lebih efektif.

Pedoman umum dalam memberikan pertanyaan:

- **Memiliki tujuan.** Ajukan pertanyaan yang memiliki tujuan sehingga arah pembicaraan dapat diketahui dengan pasti. Basa basi juga merupakan tujuan, jika memang dilakukan di awal untuk membuka pembicaraan, namun upaya tidak berlebihan.
- Pertanyaan "berisi". Ajukan pertanyaan yang memiliki isi atau substansi. Seperti di Poin 1, ketika kita memiliki tujuan bertanya, maka pertanyaan tersebut harus diisi dengan mengarahkan bentuk jawaban yang diharapkan. Dengan begitu, kita dapat mengumpulkan informasi yang berguna atau mendorong lawan bicara untuk berpikir tentang sesuatu.
- **Perlahan.** Jangan terburu-buru dalam menyampaikan pertanyaan. Lakukan dengan perlahan dan bertahap. Banyak orang perlu membangun kepercayaan dengan seseorang terlebih dahulu sebelum mereka merasa nyaman menjawab, terutama jika ditanyakan hal-hal yang bersifat pribadi.
- **Secukupnya.** Jangan terlalu banyak bertanya sehingga lawan bicara merasa "diinterogasi".

Selain itu, kita harus dapat memperhatikan hal-hal yang berpotensi memberikan masalah ketika bertanya.

1. **Pertanyaan pengarah.**

Jenis pertanyaan ini umumnya diawali dengan asumsi yang sudah terbentuk di kepala kita saat bertanya, seolah-olah kita sudah tahu jawabannya atau ingin orang lain menjawab sesuai harapan kita. Misalnya, saat menanggapi mengapa lawan bicara kita belum memberi tahu penyakitnya ke pasangannya, kita bertanya balik,

"Bukankah hal seperti itu mudah? Jadi, kapan Bapak/Ibu akan memberi tahu pasangan Bapak/Ibu mengenai penyakit ini?"

2. **Pertanyaan mengapa.**

Pertanyaan 'mengapa' sebenarnya bermanfaat untuk mengeksplorasi alas an lawan bicara. Namun, tipe pertanyaan 'mengapa' ini dapat membuat orang menjadi defensif, terutama jika disampaikan dengan intonasi interogatif. Pertanyaan semacam ini dapat menyiratkan bahwa orang tersebut seharusnya tahu jawaban atau seolah-olah kita meragukan penilaian mereka. Misalnya,

"Tadi Ibu bilang kalau Ibu tidak berhasil mengatasi perasaan bersalah itu. Mengapa? Mengapa Ibu tidak bisa memahami perasaan Ibu sendiri?"

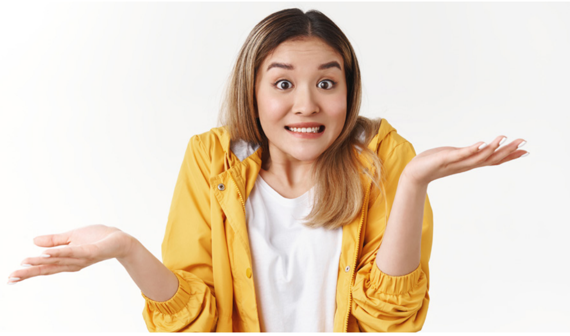

3. **Pertanyaan sensitif dan terlalu pribadi.**

Pertanyaan yang sensitif dan terlalu pribadi, tanpa ada konteks dan relevansinya, tidak pantas diajukan. Hal ini memang sulit dikenali dalam pendampingan, terutama ketika seseorang mengalami stigmatisasi, diskriminasi, atau masalah mental. Untuk itu, kita perlu mencoba mengajukan pertanyaan awalan yang dapat membantu membuka percakapan intim sebelum menanyakan hal tersebut. Selain itu, kita harus selalu bersikap hormat dan memperlakukan orang lain seperti yang kita inginkan diperlakukan. Hindari pertanyaan-pertanyaan yang bersifat seksual *(voyeuristic)*. Misalnya, kita sedang mencari tahu apakah ada dampak terhadap hubungan suami-istri setelah seseorang didiagnosis TBC. Kita bertanya,

"Jika Ibu mengalami masalah dengan suami karena penyakit ini, lalu apa yang Ibu lakukan ketika berdua di kamar?"

Pertanyaan ini sebenarnya tidak bermasalah saat kita ingin mengeksplorasi hubungan personal yang berubah karena seseorang terdiagnosis TBC. Tetapi, ini dapat menjadi masalah karena berkaitan dengan hal sensitif jika kita tidak memulainya dengan hati-hati dan memperhatikan situasi saat melakukan pembicaraan tersebut.

4. **Pertanyaan yang tidak tepat waktu atau memotong.**

Seringkali kita merasa perlu bertanya pada hal-hal yang muncul di tengah pembicaraan. Namun, mengungkapkan pertanyaan seperti ini dapat mengganggu alur seseorang menceritakan kisah mereka. Selain tidak sopan, pertanyaan ini dapat tiba-tiba mengakhiri percakapan. Misalnya,

"Kalau mereka bersikap seperti itu, apa yang Anda lakukan?" (ditanyakan di tengah-tengah seseorang bercerita mengenai pengalamannya ditinggalkan teman-temannya).

Pertanyaan Tertutup dan Terbuka

Dalam mengemukakan pertanyaan, ada dua jenis pertanyaan yang dapat kita gunakan, yaitu pertanyaan tertutup dan pertanyaan terbuka.

Pertanyaan tertutup

Pertanyaan tertutup sering dimulai dengan kata kerja, seperti apakah, dapatkah, atau akankah, yang dapat dijawab dengan respons 'Ya' atau 'Tidak'. Jika kita memerlukan informasi spesifik yang dapat dijawab dengan 'Ya' atau 'Tidak', maka pertanyaan tertutup dapat digunakan. Selain itu, pertanyaan tertutup berguna untuk memeriksa fakta dan meminta klarifikasi.

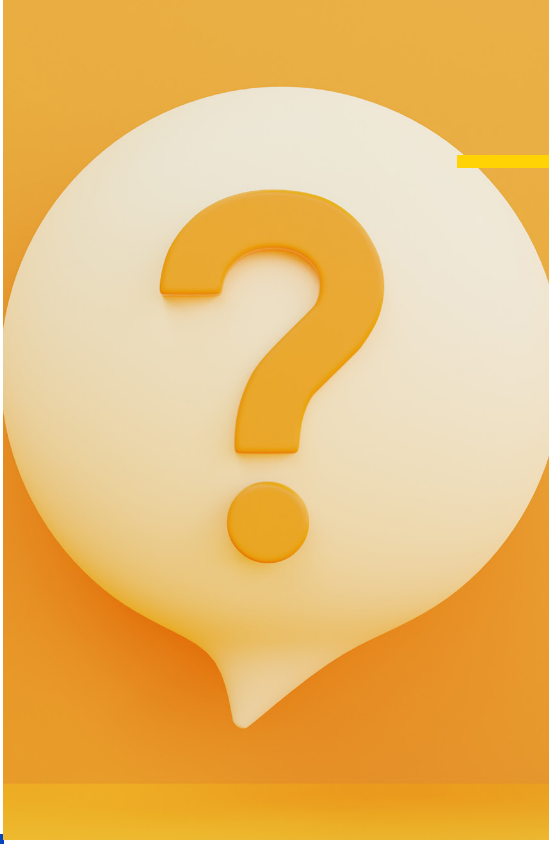

Jika digunakan dengan tepat, pertanyaan tertutup dapat berguna dalam hal berikut.

1. **Klarifikasi.** Misalnya, *"Jadi, apakah Anda merasa tertekan dengan sikap orang tersebut?"*
2. **Memeriksa informasi.** Misalnya, *"Sepertinya, Anda ingin mengatakan bahwa Anda tidak menyukai sikap yang mereka tampakkan kepada Anda. Apakah ini benar?"*
3. **Memastikan fakta.** Misalnya, *"Apakah Anda pergi ke Puskesmas setelah mengalami batuk berdarah tersebut?"*
4. **Membuat seseorang fokus pada isu tertentu.** Misalnya, *"Anda tadi menyebut soal tidak diajak makan bersama teman di tempat kerja. Apakah itu masalah khusus untuk Anda?"*

Namun, harus diperhatikan bahwa pertanyaan tertutup dapat membatasi diskusi karena hanya menimbulkan jawaban satu kata.

Misalnya, 'Apakah Anda puas dengan pelayanan kesehatan yang diberikan di Puskesmas?'. Lawan bicara umumnya hanya menjawab, 'Ya' atau 'Tidak'. Selesai.

Pertanyaan Tertutup dan Terbuka: Perhatikan Perbedaannya!

Seorang perempuan, berusia sekitar 30 tahun, baru saja didiagnosis TBC. Dia tampak khawatir dengan penyakitnya tersebut dan merasa orang-orang di lingkungannya mulai menjauhinya karena ia sering batuk. Ia bekerja sebagai kasir di sebuaht toko swalayan terkemuka. Bosnya sudah mengatakan bahwa jika batuknya tidak sembuh dalam sepekan ke depan, dia terpaksa harus diberhentikan.

Dia berusaha sekuat mungkin untuk mengobati keluhan batuknya dengan minum beberapa obat, tetapi tidak sembuh juga. Saat ke Puskesmas, ia diberitahu kemungkinan mengalami TBC dan, jika terbukti terdiagnosis TBC, ia harus menjalani pengobatan selama enam bulan. Informasi itu membuatnya semakin cemas karena ia khawatir kehilangan pekerjaan, sedangkan ia masih merupakan tulang punggung keluarganya. Ia merasa bersalah karena selama ini tidak makan dengan cukup dan teratur, serta belakangan mulai mencoba minum-minum beralkohol.

Contoh pertanyaan tertutup:

- Apakah Ibu sudah mencoba berbagi kekhawatiran tersebut dengan teman Ibu?
- Berapa banyak orang yang bekerja di sekitar Ibu?
- Apakah Ibu menikmati pekerjaan Ibu saat ini?
- Apakah ada pekerja lain yang batuk seperti Ibu?
- pakah keluarga Ibu tahu tentang kemungkinan penyakit Ibu?

Contoh pertanyaan terbuka:

- Apa yang menurut Ibu dapat mengurangi kekhawatiran tersebut?
- Bagaimana teman kerja Ibu selama ini bersikap kepada Ibu?
- Seberapa senang Ibu dengan pekerjaan ini, bisa ceritakan kepada saya?
- Bagaimana jika ada pekerja lain yang batuk seperti Ibu?
- Bagaimana Ibu ingin menceritakan kemungkinan penyakit ini kepada keluarga Ibu?

Pertanyaan Terbuka

Pertanyaan terbuka dapat digunakan untuk mendapatkan jawaban yang lebih lengkap dan bermakna, dan saat kita membutuhkan penjelasan atau gambaran tentang sesuatu. Pertanyaan terbuka seperti ini mendorong lawan bicara untuk bereksplorasi dengan cerita mereka. Tujuannya adalah untuk mendapatkan lebih banyak detail dan pemahaman sehingga masalah yang dibahas menjadi lebih jelas. Ketika digunakan, pertanyaan terbuka membantu seseorang untuk mengembangkan pengalaman, perilaku, dan perasaan yang relevan secara spesifik.

Berikut ini beberapa saran bagaimana menggunakan pertanyaan terbuka.

1. **Untuk memulai diskusi, gunakan kata tanya 'Apa' (bukan 'apakah') dan 'Bagaimana'.** Misalnya, *"Bagaimana perasaan Bapak ketika Bapak mendengar diagnosis TBC? Apa yang Bapak rasakan saat petugas Puskesmas menyampaikan hasil pemeriksaan tersebut?"*
2. **Untuk meminta deskripsi, kalimat pertanyaan dapat dimodifikasi menjadi permintaan.** Misalnya, *"Jelaskan atau ceritakan apa yang Ibu pikirkan waktu Ibu melihat ada darah di tangan Ibu saat batuk."*
3. **Untuk memberi kesempatan pada seseorang untuk memperluas apa yang dikatakan.** Misalnya, *"Tadi Mbak bilang bahwa Mbak merasa kaget dengan diagnosis tersebut. Ceritakan apa yang Mbak rasakan saat itu. Dalam hal apa saja perasaan itu biasanya muncul?"*
4. **Fokus pada perasaan.** Misalnya, *"Bagaimana perasaan Bapak saat diperlakukan seperti itu?"*
5. **Fokus pada rencana.** Misalnya, *"Bagaimana rencana Ibu setelah tahu pengobatan ini harus dilakukan enam bulan?"*

05.

Workshop 5
Peran dan Batasan, Situasi yang Menantang, dan Kerahasiaan

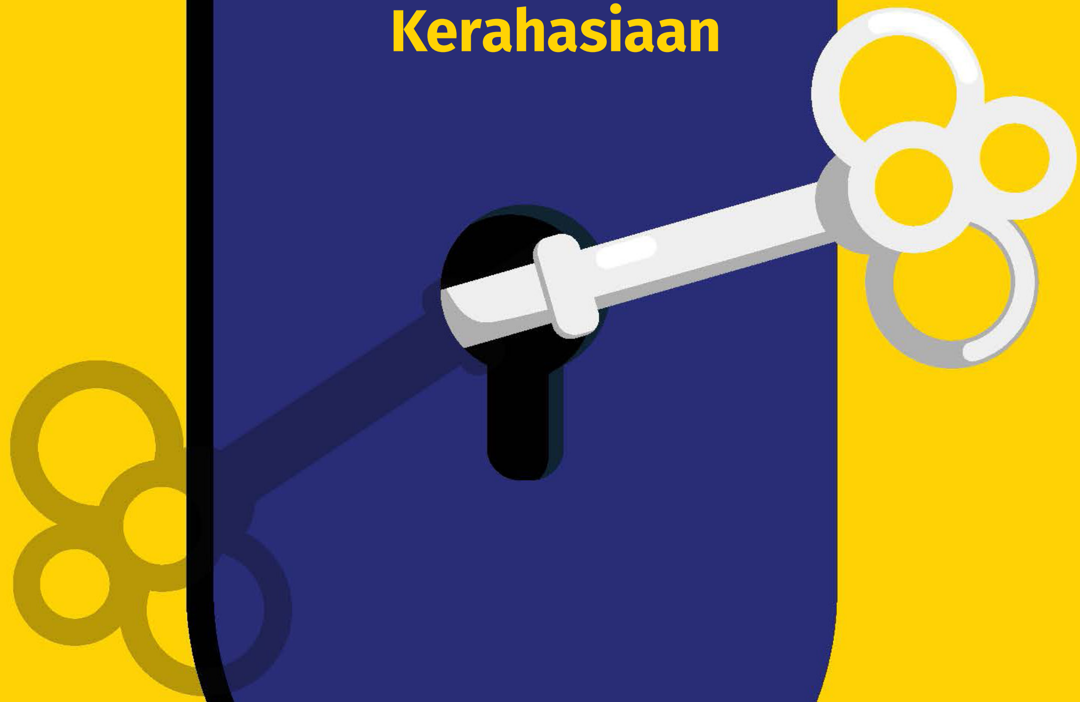

Peran dan Batasan

The Helping Relationship

- Menyadari nilai yang dianut oleh pendukung dan teman sebaya, serta proses pendampingan yang dilakukan
- Memperjelas mengenai pengetahuan dan pengalaman yang dimiliki
- Bersikap tegas mengenai kerahasiaan dan batasannya
- Mengingat bahwa hubungan dan percakapan yang terjadi mengenai orang lain, bukan diri kita
- Pastikan bahwa mengungkap kerahasiaan status dilakukan dengan tepat
- Mendapatkan bantuan dan meminta saran jika dirasa tidak yakin atau merasa kewalahan
- Menyarankan teman sebaya ke profesional kesehatan mental jika diperlukan
- Ketahui keterbatasan dalam membantu teman sebaya
- Merasa nyaman untuk mendiskusikan keterlibatan pendukung dan mengatakan tidak jika diperlukan
- Memproteksi diri sendiri saat proses pemberian dukungan bagi teman sebaya

Prinsip Etika dalam Pendampingan Teman Sebaya

Ada enam prinsip utama dalam Pendampingan Teman Sebaya

Otonomi
(Autonomy)

Tindakan tanpa bahaya
(Non-maleficence)

Adil
(Justice)

Jujur
(Veracity)

Menepati Janji
(Fidelity)

Menjaga Kerahasiaan
(Confidentiality)

Tindakan tanpa bahaya

Dalam prinsip ini, kita harus memastikan bahwa apa yang kita lakukan terhadap orang dengan TBC dalam proses pendampingan psikososial tidak sampai membahayakan mereka. Oleh karena itu, kita harus melakukan tindakan sesuai standar atau protokol yang telah ditetapkan atau menyingkirkan keadaan atau kondisi spesifik yang dapat merugikan mereka

Adil

Prinsip ini mendorong kita untuk selalu memperlakukan orang dengan TBC sesuai dengan haknya, tanpa membeda-bedakan dan diskriminatif, dengan alasan apapun. Misalnya, memperlakukan semua orang dengan TBC mendapatkan pendampingan psikososial sesuai dengan standar dan protokol yang berlaku, tanpa membeda-bedakan berdasarkan latar belakang suku, agama, ras dan antar golongan (SARA).

Jujur

Dalam prinsip ini, kita harus mengatakan dan memperlakukan orang dengan TBC sesuai dengan hal yang sebenarnya, tidak dibuat-buat, atau tidak disertai dengan kebohongan. Misalnya, kita memberikan informasi yang benar meskipun mungkin tidak sesuai dengan apa yang mereka harapkan. Sampaikan informasi tersebut dengan susunan bahasa yang baik dan cara yang nyaman, serta tidak membohongi pasien

Menepati Janji

Prinsip ini mendorong kita untuk selalu memperlakukan pasien sesuai dengan komitmen yang telah disepakati bersama. Dalam memberikan dukungan sosial, misalnya, kita memulai pertemuan kelompok tepat waktu, menepati janji atau poin-poin kesepakatan yang disepakati di pertemuan sebelumnya. Jika ada sesuatu yang perlu diubah, kita harus memberitahu dan berkomunikasi terlebih dahulu kepada mereka dan menjelaskan tentang penyebab, efek, dan segala dampak perubahannya.

Menjaga Kerahasiaan

Prinsip ini menuntun Pendukung teman sebaya *(Peer Supporter)* untuk mengetahui kapan **kerahasiaan harus dijaga dan kapan perlu diberitahukan. Prinsip umumnya adalah kerahasiaan harus selalu dijaga, kecuali dapat menyebabkan kerugian langsung pada diri teman sebaya *(Peer Support)* atau orang lain.** Misalnya, tidak membagikan informasi personal orang dengan TBC ke orang lain, memastikan bahwa tidak ada report berisi data personal yang tercecer, dan memastikan identitas personal disamarkan di laporan yang bisa diakses oleh masyarakat luas atau bahan presentasi.

Jika teman sebaya *(Peer)* mengungkapkan informasi yang ingin kita tetap rahasiakan, namun kita merasa bahwa hal tersebut perlu diberitahukan, lihat dan nilailah situasinya dengan bertanya pada diri sendiri mengenai:

- Mengapa kita merasa perlu untuk melanggar kerahasiaan?
- Seberapa serius situasinya sehingga kita harus membocorkan kerahasiaan?
- Risiko apa yang akan dihadapi oleh teman sebaya *(Peer)*?
- Apakah rahasia ini akan berakibat pada risiko yang signifikan?
- Jika rahasia dilanggar, apa implikasinya, baik positif dan negatif?
- Dampak apa yang dapat ditimbulkan (bagi orang lain dan diri sendiri)?

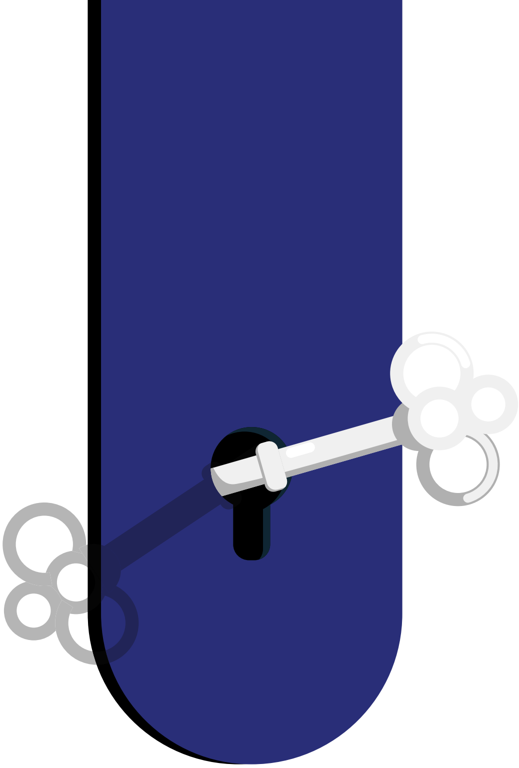

Jika kita memiliki alasan yang jelas dan baik untuk melanggar kerahasiaan, cobalah membatasi konsekuensi negatifnya dengan:

- Menjelaskan kepada teman sebaya *(Peer)* mengenai alasan kita melanggar kerahasiaan
- Memperjelas tentang:
  - Mengapa kita mengungkapkan kerahasiaan?
  - Siapa yang akan kita ceritakan?
  - Apa yang akan kita katakan kepada mereka?
  - Apa yang mungkin mereka lakukan dengan informasi tersebut?
  - Apa konsekuensinya bagi teman sebaya (Peer)?

Dalam membuat keputusan sulit yang memaksa kita mengungkapkan kerahasiaan, kita harus mendiskusikan situasi tersebut dengan orang yang memiliki keahlian atau kualifikasi, seperti tim peneliti, konselor, atau dokter, tanpa menyebutkan nama atau detail (sehingga tetap mempertahankan kerahasiaan). Mereka dapat membantu kita memutuskan tindakan terbaik yang harus diambil. Selain itu, jika kita merasa tidak sanggup untuk memutuskan informasi yang akan diungkapkan, dapat berbicara dengan orang yang lebih berpengalaman atau profesional lainnya untuk mendapatkan pandangan atau pendapat mereka.

Menangani *Disclosure* (pengungkapan)

Berikut adalah beberapa saran umum untuk menghadapi orang yang menceritakan masalahnya kepada kita:

- **Dengarkan baik-baik.** Dukungan dan dorongan kita sangat penting.
- **Ketahui kemampuan dan batasan kita.** Terdapat beberapa situasi yang tidak bisa kita lakukan (dan tidak seharusnya) kita menanganinya sendiri. Jika ragu, rujuk teman sebaya *(Peer)* kepada profesional.
- **Berkonsultasi. Beri tahu teman sebaya *(Peer)* bahwa kita perlu berbicara dengan orang yang lebih berpengalaman sebelum memberikan nasihat langsung. Bicaralah dengan sumber tersebut, dan kemudian menghubungi teman sebaya *(Peer)* kembali sesegera mungkin.**
- **Yakinkan teman sebaya *(Peer)*** bahwa kita akan **menjaga kerahasiaan kecuali kita merasa bahwa hal tersebut dapat mengancam diri mereka sendiri atau individu lain.**
- **Lanjutkan untuk melakukan pendampingan pada teman sebaya *(Peer)* tersebut, jika diperlukan**. Pastikan mereka mengetahui bahwa kita hadir untuk mereka dan kita akan mendukung mereka saat mereka melalui permasalahannya. Jika kita tidak dapat mendampingi mereka, beritahukan kepada mereka sehingga mereka dapat berkonsultasi dengan orang lain yang dapat mereka percaya.

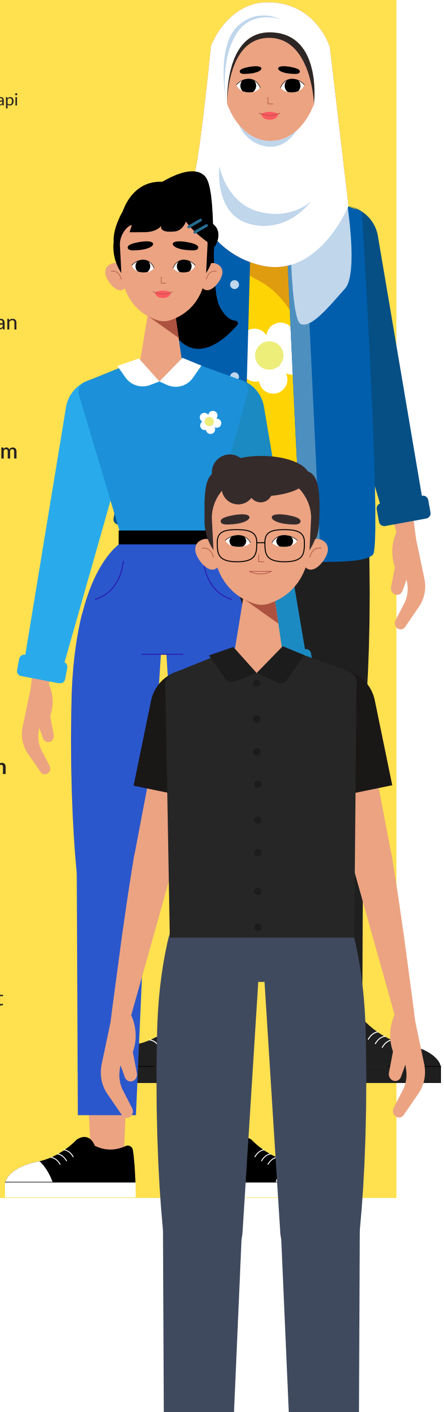

06. Workshop 6 Keterampilan Memotivasi

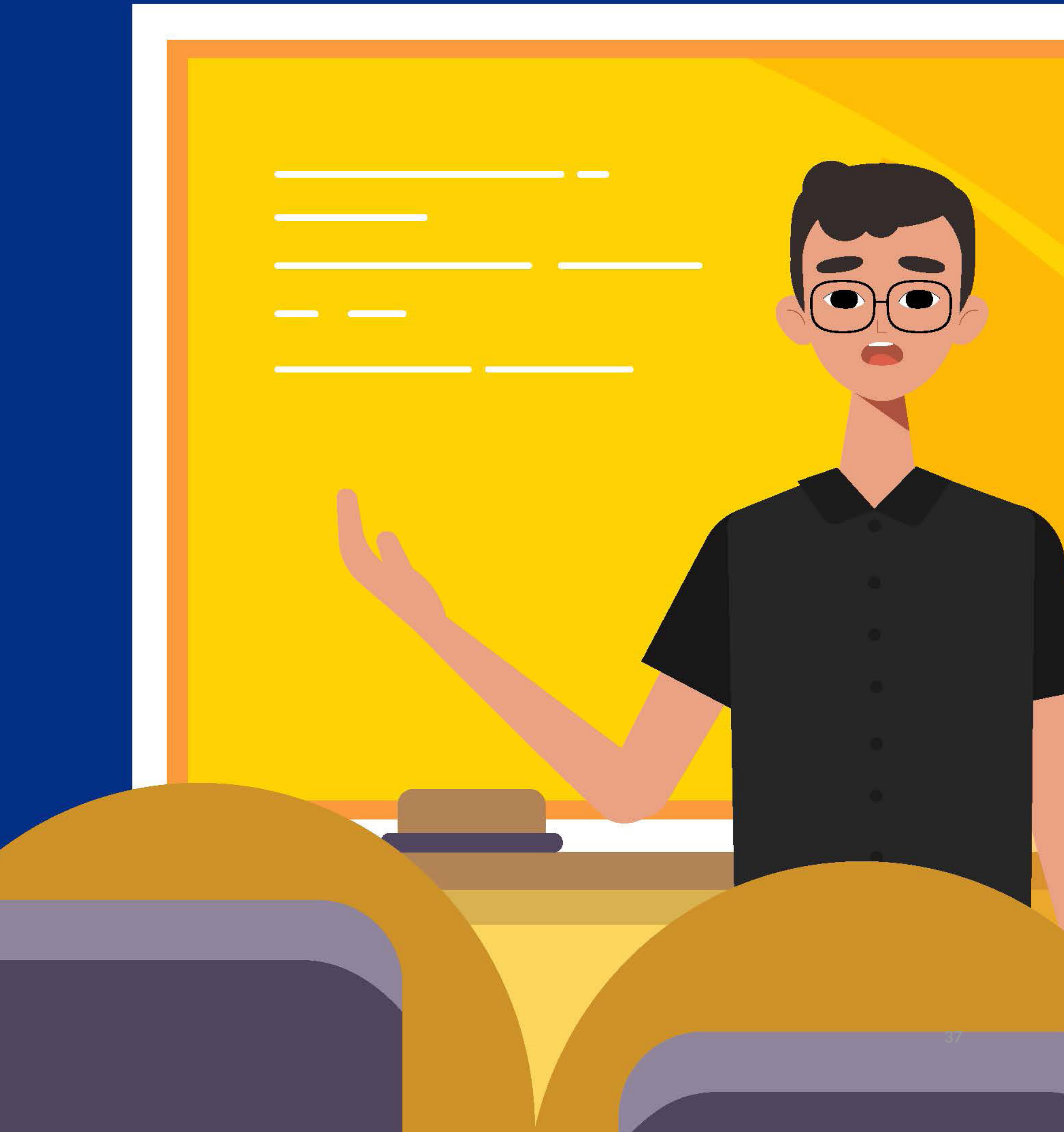

Prinsip Keterampilan Memotivasi

Peran utama seorang fasilitator di kelompok adalah memberikan bimbingan kepada anggota kelompoknya atau peserta group meeting. Dengan demikian, fasilitator kelompok perlu membangun komunikasi yang baik agar anggota kelompok dapat leluasa bercerita secara utuh terkait kondisinya saat ini. Motivasi yang diberikan pun juga dapat sesuai dengan kebutuhannya saat ini. Peranan lain yang diperlukan dalam **keterampilan memotivasi adalah memberikan instruksi *(directing)*, dan mendengarkan dan memahami, mengikuti alur berbicara peserta/anggota *(following)*.**

Prinsip yang diperlukan dalam menjalankan keterampilan memotivasi adalah:

1. Fokus pada kondisi peserta kelompok saat ini
2. Pastikan sudah di izinkan untuk memberikan saran kepada peserta kelompok
3. Fokus pada apa yang akan terjadi pada peserta kelompok, misalnya dampak dari saran yang kita berikan, cara peserta dalam melaksanakan saran tersebut, rencana peserta berikutnya setelah bertemu dengan kita

Kunci Membangkitkan Motivasi

Terdapat empat kunci dalam membangkitkan motivasi:

1. **Penerimaan**

   Fasilitator perlu memiliki kemampuan dalam menerima anggota dengan apa adanya untuk memahami kondisi mereka dan membimbingnya menuju perubahan

2. **Kolaborasi**

   Terjadi kolaborasi antara fasilitator dan anggota agar dapat bekerja sama dalam menyusun langkah strategis untuk bisa dimanfaatkan oleh peserta dalam melakukan perubahan

3. **Pemahaman konteks**

   Fasilitator memahami konteks atau kondisi peserta untuk menyempurnakan motivasi yang diberikan sehingga dapat membantu fasilitator dalam mengarahkan peserta untuk perubahan yang diinginkan

4. **Pemahaman potensi**

   Fasilitator memahami potensi peserta sehingga bimbingan yang diberikan dapat sesuai dengan kemampuan pelaksanaan peserta untuk dapat membantu terwujudnya tujuan yang realistis dari perubahan yang akan dilakukan

07.

Workshop 7
Mengenali dan Merespons Masalah Mental, Kapan Kita Perlu Mencari Pertolongan dan Merujuk Pada Profesional Kesehatan Jiwa

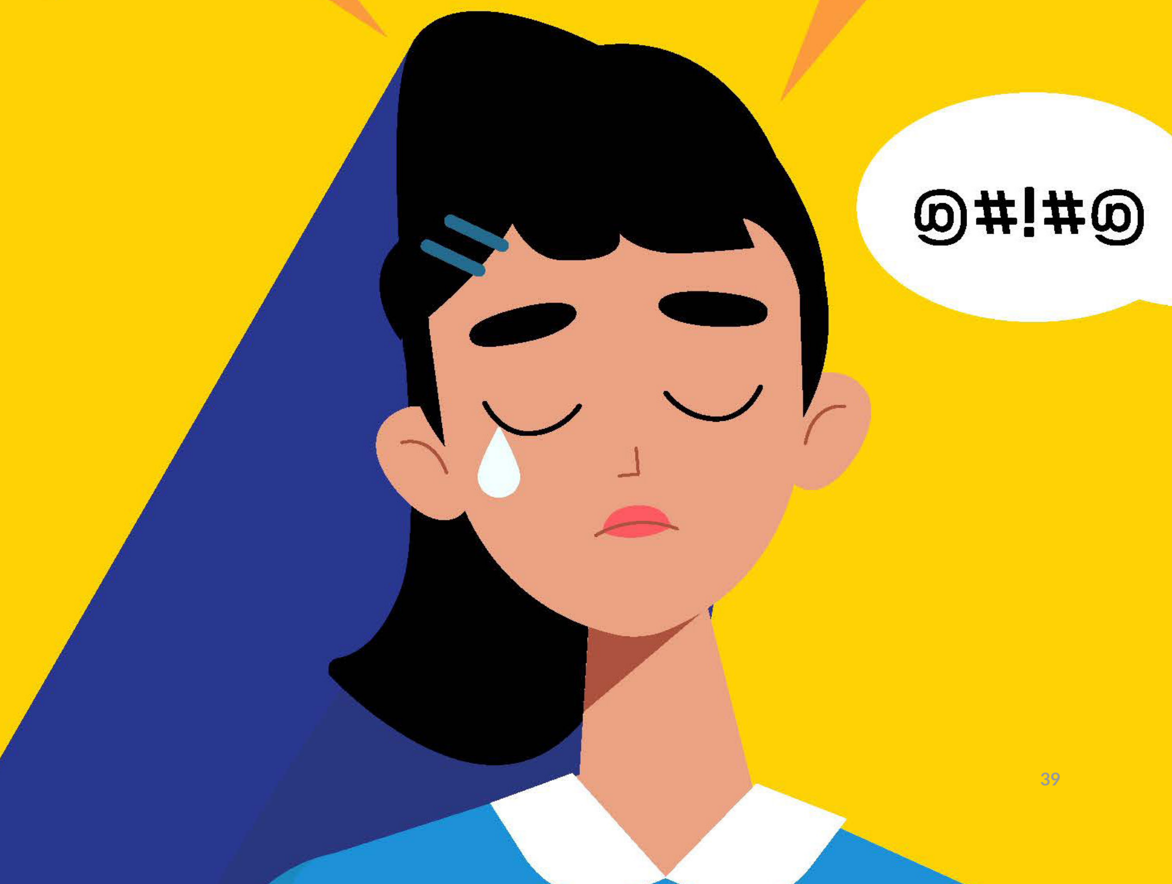

Kesehatan Jiwa

Pengertian Kesehatan Jiwa?

Kesehatan Jiwa adalah kondisi di mana seorang individu dapat berkembang secara fsik, mental, spiritual, dan sosial, sehingga individu tersebut menyadari kemampuan sendiri, dapat mengaa tasi tekanan, dapat bekerja secara produktf, dan mampu memberikan kontribusi untuk komunitasnya.

Undang-Undang Nomor 18 tahun 2014 tentang Kesehatan Jiwa

Apa, Kapan, dan Kemana Akan Merujuk?

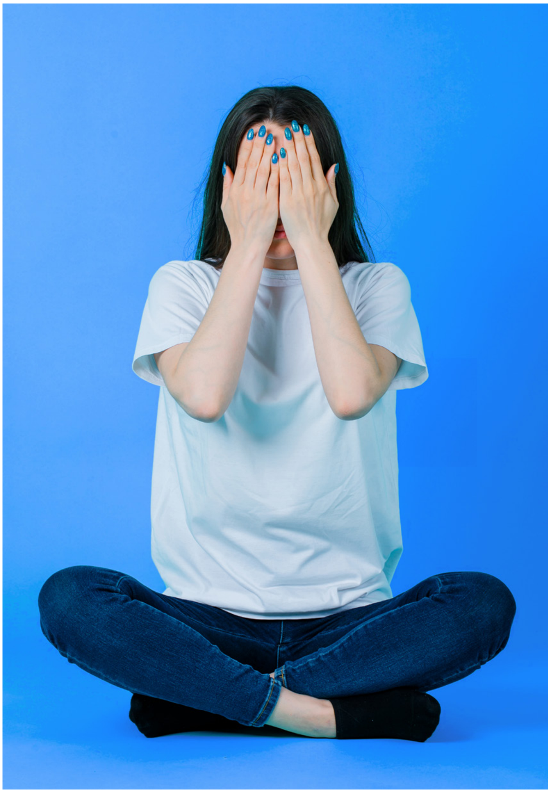

Apa

Membuat rujukan untuk membantu seseorang mengakses sumber dukungan lain yang lebih profesional untuk mengatasi masalah kejiwaan yang dialaminya.

Kapan

1. Masalah yang memerlukan bantuan profesional Kesehatan jiwa

2. Anda khawatir orang tersebut akan menyakiti dirinya atau terlibat semakin dalam di dalam masalahnya

Anda mengkhawatirkan kondisi Anda sendiri (terdapat masalah yang dialami saat ini)

Masalah kesehatan jiwa yang membutuhkan bantuan profesional.

Dalam modul ini, akan dipaparkan dua masalah kejiwaan utama yang perlu diidentifikasi dan dirujuk, jika diperlukan, yaitu depresi dan cemas *(anxiety)*.

Perasaan Cemas

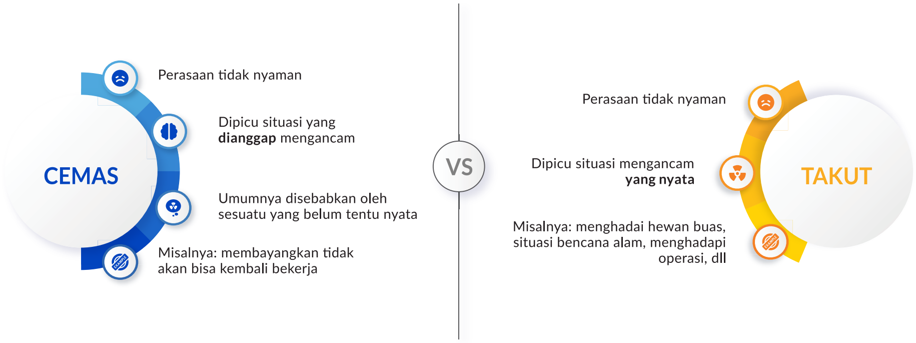

Efek pengobatan TBC yang dapat bervariatif pada setiap individu → Orang dengan TBC tidak dapat mendeteksi atau memperkirakan apa yang akan mereka alami di waktu yang akan datang

Untuk dapat menggali kecemasan yang dialami oleh orang dengan TBC, kita perlu mahir bertanya, terutama mengenai hal apa yang dipikirkan oleh mereka. Seringkali banyak orang belum memahami perbedaan antara takut dan cemas. Perasaan takut dipicu oleh situasi mengancam yang nyata, sedangkan cemas muncul karena situasi yang dianggap mengancam.

Gejala Cemas

01	**FISIK**	• Jantung berdebar lebih cepat<br>• Otot tegang	• Tarikan nafas menjadi pendek<br>• Sakit perut	• Tangan berkeringat<br>• Gemetar
02	**PSIKOLOGIS**	Sulit konsentrasi	Sulit mengingat	Pikiran terus
03	**PERILAKU**	Fidgeting	Menghindari situasi	Kesulitan untuk tidur

Beberapa hal yang dapat menimbulkan cemas pada orang dengan TBC:

- Membayangkan ketidaknyamanan karena efek minum obat
- Membayangkan apakah dirinya tidak bisa kembali bekerja
- Pikiran mengenai ia tidak dapat kembali ke kehidupan sebelumnya
- Pemikiran mengenai respon orang sekitar saat mengetahui dirinya diagnosis TBC, misalnya kecemasan akan dikucilkan atau ditinggalkan.

Deteksi dini penting dilakukan sebelum kondisi perasaan cemas menjadi lebih parah.

Depresi

Depresi adalah keadaan ketika seseorang mengalami gangguan suasana hati yang ditandai dengan penurunan emosi atau perasaan sedih yang berkelanjutan dan kehilangan minat terhadap aktivitas yang sebelumnya dinikmati.

Secara umum, gejala depresi pada orang dengan TBC dapat dilihat melalui dua kondisi, yaitu psikis dan fisik.

Gejala Psikis	Gejala Fisik
• Merasa rendah diri, putus asa, dan tidak berharga.<br>• Merasa khawatir dan cemas berlebihan.<br>• Sangat sensitif, seperti mudah marah, tersinggung, atau sedih.<br>• Sulit untuk memusatkan fokus dan konsentrasi.<br>• Kesulitan untuk berpikir dan mengambil keputusan.<br>• Cenderung menutup diri dari lingkungan sosial.<br>• Menurunnya ketertarikan, minat, atau motivasi untuk melakukan apa pun.	• Terlalu banyak tidur atau bahkan insomnia.<br>• Peningkatan atau bahkan penurunan nafsu makan secara drastis.<br>• Mudah lelah dan tidak bertenaga.<br>• Nyeri pada bagian tubuh tertentu tanpa diketahui penyebab pastinya (gangguan psikosomatik).<br>• Berat badan turun ataupun naik secara drastis.<br>• Penurunan gairah seksual.

Identifikasi

Penapisan masalah kejiwaan dapat dilakukan menggunakan kuesioner yang sudah valid. Dalam hal ini, kita menggunakan kuesioner Patient Health Questionnaire (PHQ-9) untuk mengidentifikasi depresi. Meski demikian, kita tetap perlu melakukan observasi terhadap orang dengan TBC sehingga dapat melakukan identifikasi kemungkinan seseorang mengalami masalah kejiwaan.

PHQ-9 merupakan instrumen yang digunakan untuk penapisan gangguan kejiwaan, khususnya identifikasi gangguan depresi. Pertanyaan-pertanyaan pada instrumen ini dibuat berdasarkan kriteria diagnosis depresi dalam Diagnostic and Statistical Manual of Mental Disorder, 4th edition (DSM-IV), berisi sembilan pertanyaan pendek mengenai kondisi individu dalam dua minggu terakhir.

Instrumen ini dapat diisi secara mandiri atau ditanyakan oleh petugas kesehatan atau tim peneliti. Pengisian instrumen dilakukan dengan memberikan penilaian pada setiap pernyataan menggunakan rating angka 0 sampai 3. Hasil penjumlahan nilai jawaban yang dipilih pada seluruh pertanyaan menjadi acuan interpretasi apakah seseorang mengalami depresi atau tidak.

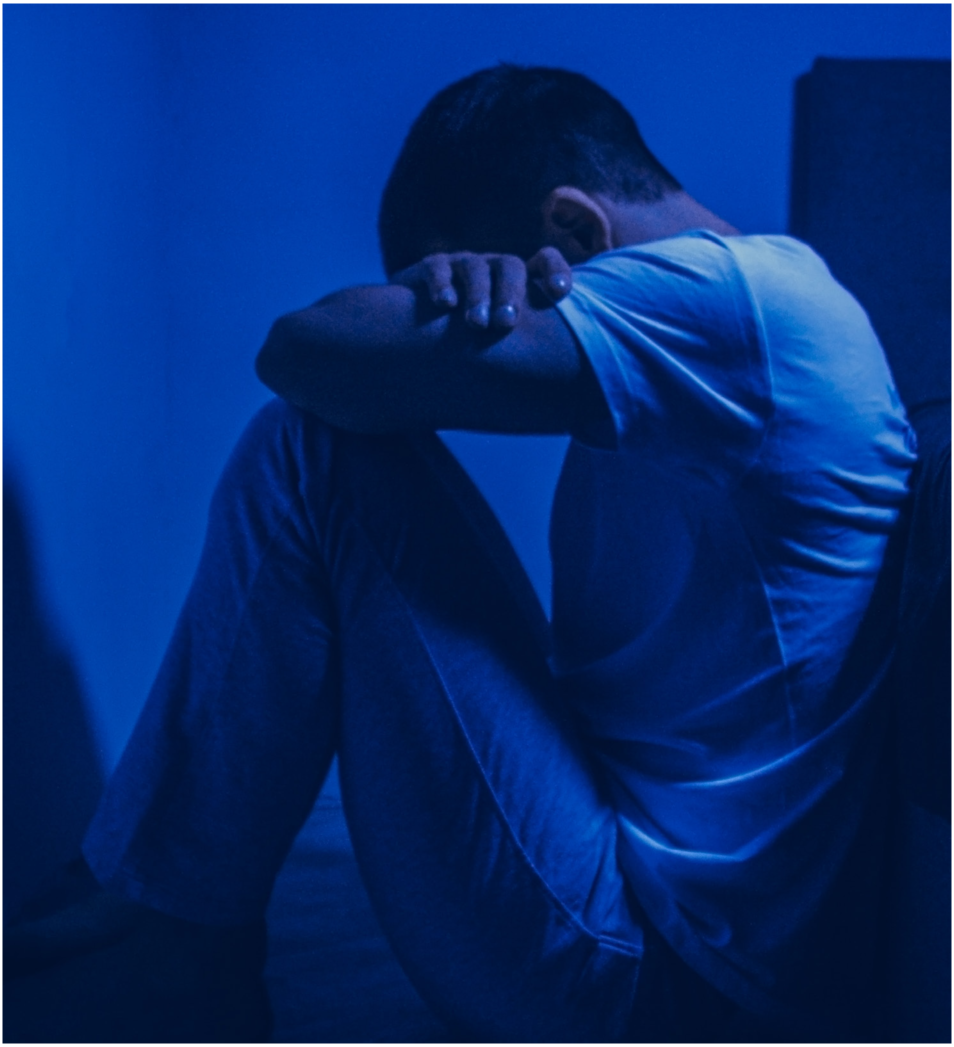

Kuesioner PHQ-9

Di bawah ini ada beberapa pernyataan yang menggambarkan bagaimana situasi yang Anda rasakan selama dua pekan terakhir. Silakan memberikan pendapat Anda dalam skala 0-3 sesuai dengan pernyataan berikut.

0 = “Tidak sama sekali”;
1 = “beberapa hari”;
2 = “Lebih dari tujuh hari”;
3 = “Hampir setiap hari”

C. Selama 2 pekan terakhir, seberapa sering Anda terganggu oleh beberapa masalah berikut ini?	Tidak sama sekali	Beberapa hari	Lebih dari 7 hari	Hampir setiap hari
1. Kurang tertarik atau bergairah dalam melakukan apapun				
2. Merasa murung, muram, atau putus asa				
3. Sulit tidur atau mudah bangun, atau terlalu banyak tidur				
4. Merasa lelah atau kurang bertenaga				
5. Kurang nafsu makan atau terlalu banyak makan				
6. Kurang percaya diri – atau merasa bahwa Anda adalah orang yang gagal atau telah mengecewakan diri sendiri atau keluarga				
7. Sulit berkonsentrasi pada sesuatu, misalnya membaca koran atau menonton televisi				
8. Bergerak atau berbicara sangat lambat sehingga orang lain memperhatiannya. Atau sebaliknya, merasa resah atau gelisah sehingga Anda lebih sering bergerak daripada biasanya				
9. Merasa lebih baik mati atau melukai diri sendiri dengan cara apapun				

Ke mana akan MERUJUK

Profesional Kesehatan jiwa di tim peneliti, Puskesmas (jika ada), atau Rumah Sakit terdekat.

Bagaimana cara membuat rujukan

- Kita harus mengetahui masalah yang dihadapi oleh orang dengan TBC tersebut sebelum kita membuat rujukan
- Pilih kata-kata dengan hati-hati
- Gunakan frasa seperti:
  - Bapak mungkin akan lebih terbantu jika…
  - Ibu mungkin ingin berbicara lebih lanjut dengan…
  - Apakah Mbak berpikir untuk berbicara dengan…
  - Saya pikir ini mungkin berguna bagi Bapak untuk…
- Berikan kata-kata dengan jujur. Beri tahu orang dengan TBC tersebut mengapa kita bukan orang terbaik atau mengapa orang lain mungkin bisa membantu lebih baik pada saat itu
- Kita dapat terus memberikan dukungan dengan tetap berhubungan dan menanyakan kabar
- Ketahuilah keterbatasan kita dan jangan melampaui batas kemampuan kita

Tips untuk membuat rujukan bagi mereka yang menolak

Jika kita yakin bahwa orang dengan TBC akan mendapat manfaat dari bantuan profesional, langkah terbaik adalah menyampaikannya dengan jujur mengenai alasan kita dan ungkapkan kekhawatiran kita tentang masalah yang dialami oleh mereka. Namun, terkadang orang enggan atau malu untuk meminta atau menerima bantuan, berikut beberapa saran yang dapat diberikan:

- **Hilangkan mitos seputar mencari bantuan masalah kejiwaan.** Jelaskan bahwa menemui seorang konselor atau profesional Kesehatan jiwa dapat membantu dan bukan berarti orang dengan TBC tersebut mengalami gangguan kesehatan jiwa
- Sarankan bahwa **mendapatkan bantuan adalah hal yang positif**
- Beberapa orang mungkin merasa tidak nyaman menemui konselor atau profesional Kesehatan jiwa, namun mereka akan setuju untuk mengunjungi dokter umum. Orang lain mungkin memilih untuk berbicara dengan pemuka agama atau menghubungi kelompok dukungan. Oleh karena itu, sangat membantu dengan **menyajikan semua opsi dan mendiskusikannya dengan orang dengan TBC tersebut**
- Jika orang dengan TBC tersebut enggan mencari bantuan, **tanyakan mengapa ia tidak tertarik untuk bertemu seseorang profesional.** Mungkin ini berkaitan dengan pengalaman negatif sebelumnya atau ketidakpahamannya
- **Jelaskan bahwa masalahnya berada di luar bidang keahlian Anda**
- Jika dia tidak yakin untuk mencari bantuan saat ini, ada baiknya jika Anda **memberikan bantuan kepada orang tersebut berupa nama dan nomor kontak yang dapat digunakan** di kemudian hari
- Jika kita yang mengatur janji temu dengan profesional kesehatan jiwa, **informasikan kepada profesional tersebut mengenai kekhawatiran spesifik kita terkait apa yang dialami orang dengan TBC** tersebut
- **Jika kita merasa situasinya darurat** (ada kemungkinan membahayakan dirinya atau orang lain) dan ia enggan menemui seorang profesional, kita mungkin perlu **berbicara dengan seseorang untuk membantu.**

08.

Workshop 8
Keterampilan Memandu Dukungan Kelompok

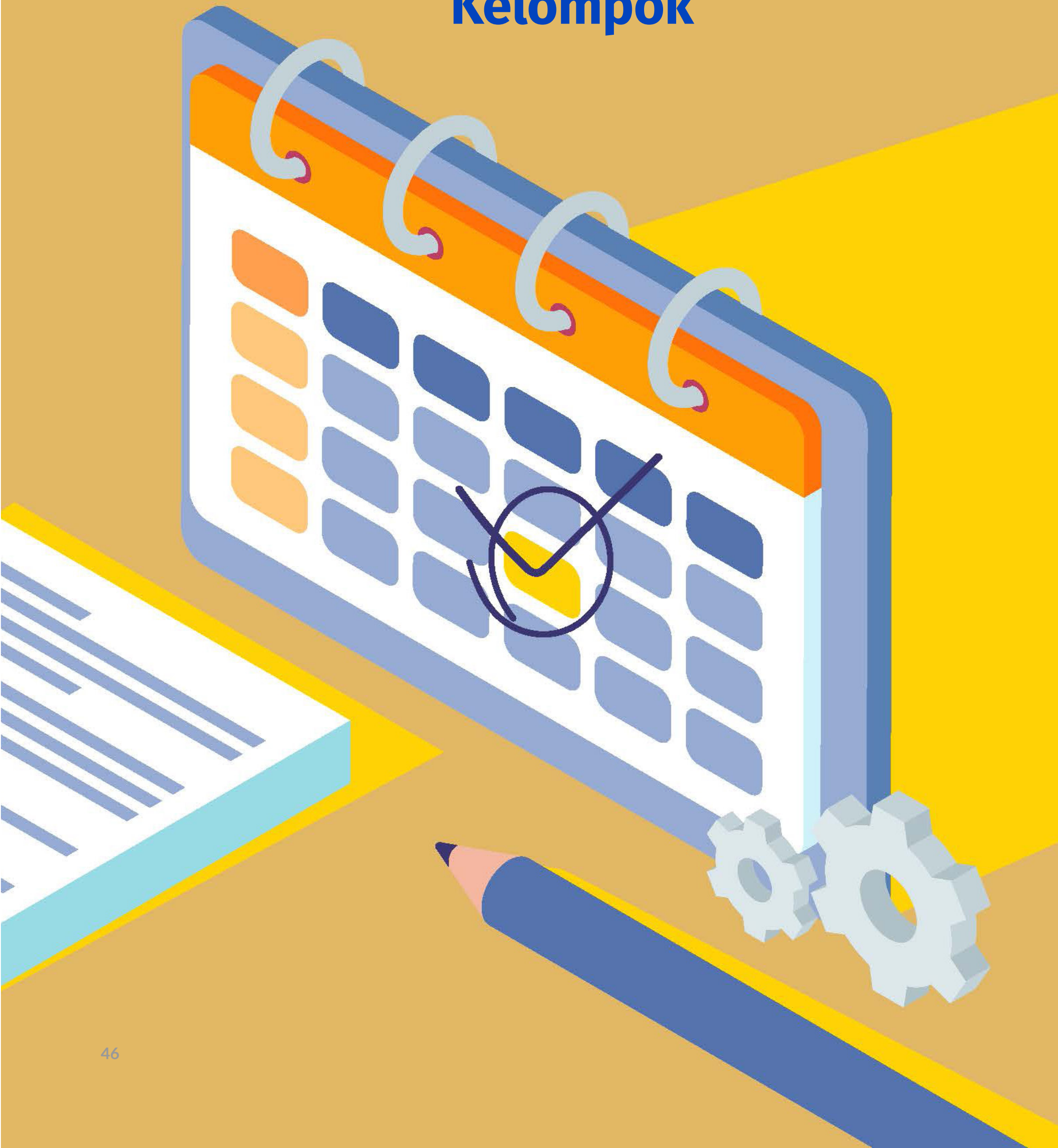

Proses memandu dukungan kelompok

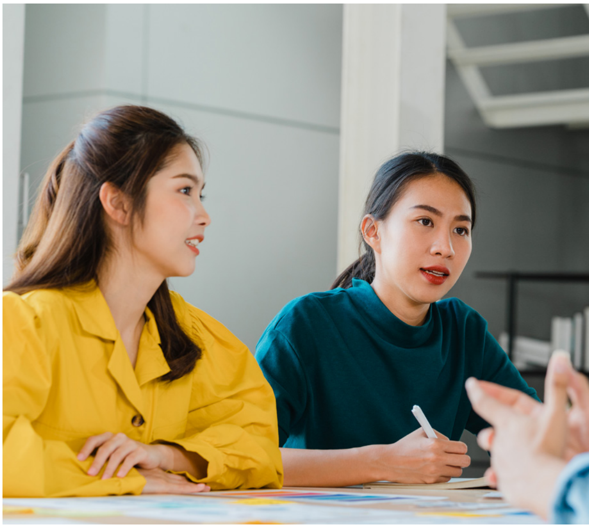

Fasilitator memiliki peran untuk mengupayakan proses dan kegiatan yang membantu kelompok mencapai tujuan. Fasilitator dalam kelompok bukanlah diktator maupun pengamat sehingga fasilitator perlu bekerja seoptimal mungkin untuk membuat setiap anggota kelompok bergerak dan berperan aktif melaksanakan dinamika kelompok. Berikut adalah beberapa hal yang dilakukan dalam memandu *support group.*

Memulai Sesi

Saat memulai sesi, terdapat beberapa hal yang dapat dilakukan agar seluruh anggota kelompok dapat berpartisipasi dalam konseling sehingga mencapai tujuan yang diinginkan.:

a. Menentukan jadwal

Penentuan jadwal terkait pertemuan maupun pembahasan dapat membantu pelaksanaan konseling grup yang lancar. Namun, pembuatan jadwal tentunya harus berdasarkan kesediaan anggota kelompok dan sebaiknya dibuat dalam jadwal yang fleksibel setelah satu sesi konseling selesai. Anggota kelompok juga diminta untuk hadir tepat waktu sebagai bentuk komitmen bersama.

b. Menyepakati tujuan bersama

Hal ini bertujuan agar pembahasan pada saat konseling grup lebih terarah, sesuai dengan persetujuan seluruh anggota kelompok. Tentunya, tujuan ini juga perlu bersifat fleksibel. Contoh dari tujuan bersama ini seperti “menjadi lebih percaya diri meskipun terkena penyakit TB”.

c. Membagi tanggung jawab

Pembagian tanggung jawab antar kelompok dapat meningkatkan partisipasi anggota kelompok. Setiap tanggung jawab dibagi sama besarnya termasuk dengan fasilitator. Beberapa tugas yang dapat dibagi adalah menjadi pemandu dalam pertemuan berikutnya, menentukan topik yang ingin dibahas, mengusulkan tempat dan waktu sesi, atau memandu ice breaking.

d. Memberi nama kelompok

Dengan memberi nama pada kelompok, rasa kebersamaan dan kepemilikan antar anggota kelompok diharapkan dapat tercipta.

Menjalankan dan mempertahankan sesi

Untuk menjalankan sebuah sesi, tentu fasilitator harus memiliki keterampilan memandu yang terus diasah. Umumnya, keterampilan yang dibutuhkan adalah keterampilan komunikasi, menunjukkan afeksi, membangun keterikatan, dan memecahkan masalah. Keterampilan tersebut perlu dikuasai sehingga dinamika kelompok akan berjalan dengan optimal.

1. **Keterampilan komunikasi,** merupakan keterampilan fasilitator dalam melakukan komunikasi dengan kelompok

2. Keterampilan afeksi, yang terdiri dari:

    a. Mengenali dan mengeksplorasi perasaan
    b. Mendapatkan umpan balik (feedback) dari peserta grup konseling
    c. Memindai perasaan dari peserta grup konseling
    d. Mengenali petunjuk nonverbal
    e. Mengonfirmasi perasaan
    f. Membantu kelompok bertahan pada perasaan agar tidak teralihkan dari perasaan peserta yang telah terbangun, sehingga perasaan dapat divalidasi
    g. Menerjemahkan perasaan

3. Keterampilan membangun keterikatan, yang terdiri dari:

    a. Menentukan batasan antar personal
    b. Menetapkan struktur dari kelompok yang sudah ditetapkan
    c. Menegaskan peran
    d. Mendorong kesepakatan kelompok
    e. Mengenali kesamaan dalam kelompok
    f. Mendorong semua anggota berperan

4. Keterampilan memecahkan masalah, yang terdiri dari:

    a. Mengenali adanya kebutuhan terkait keputusan dan membantu pelaksanaan
    b. Mengklarifikasi
    c. Memberikan saran
    d. Menentukan fokus dan prioritas
    e. Mengkonfrontasi masalah

Dengan keterampilan komunikasi tersebut, diharapkan fasilitator dapat membuat perubahan interaksi antar individu dalam kelompok.

Beberapa penyebab kemungkinan konseling kelompok tidak berjalan sesuai rencana:

- **Kedatangan anggota yang tidak dapat diprediksi,** seperti anggota yang kadang datang dan kadang tidak datang. Hal ini bisa menyebabkan anggota lain dalam kelompok merasa tidak nyaman untuk berbagi masalah dengan orang yang tidak terlalu dikenali
- Terdapat kemungkinan anggota lama **terlalu cepat memberi label (cenderung bersifat negatif)** kepada anggota baru. Misalnya, anggota baru yang menceritakan tentang kesulitan dalam mematuhi proses pengobatan dari dokter sehingga anggota lama memberikan label "payah" yang menyebabkan penurunan motivasi anggota baru dan berpotensi meningkatkan stigma dalam dirinya
- Ada kemungkinan kondisi **salah satu anggota kelompok mengalami kondisi penyakit yang lebih parah** sehingga membuat anggota lain yang dalam tahap awal menjadi ketakutan/tertekan
- **Ekspresi perasaan yang intens dari seorang anggota kelompok yang dapat menghambat** maupun membebani anggota lain yang sedang berusaha untuk sembuh. Misalnya, salah satu peserta yang menangis terus dan menyita perhatian kelompok sehingga peserta lain tidak nyaman
- **Ada kemungkinan misinformasi** karena dipimpin oleh fasilitator yang kurang profesional. Misalnya fasilitator tidak memiliki kemampuan dalam mendampingi dan mengarahkan kelompok sehingga kegiatan tidak berjalan sesuai rencana
- Ada kemungkinan **fasilitator diminta untuk memberikan terapi/pengobatan** namun tidak memiliki kompetensinya

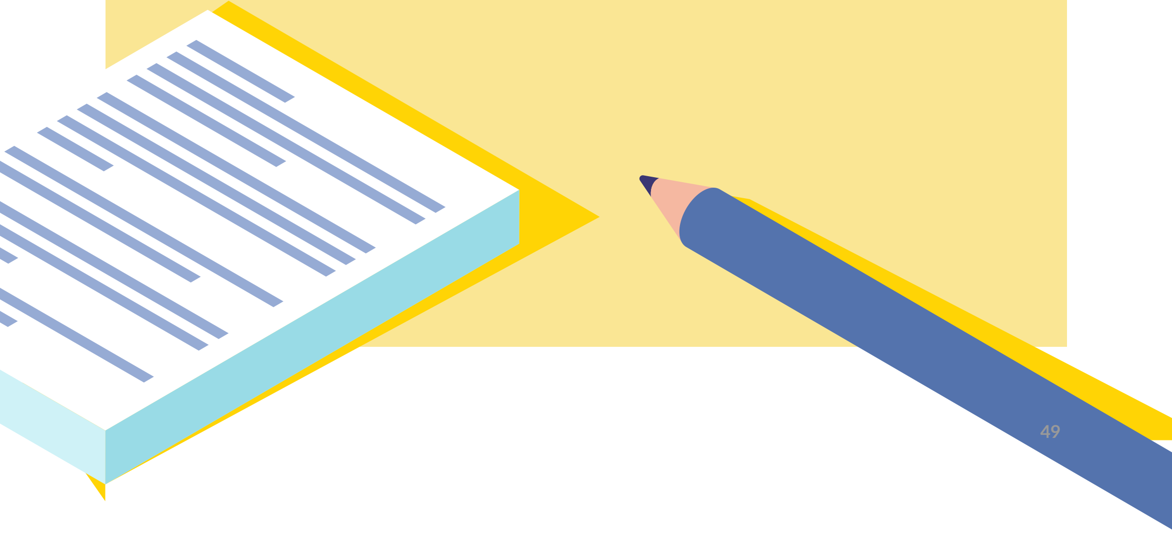

09.

Workshop 09
Self-Care
(Perawatan Diri)

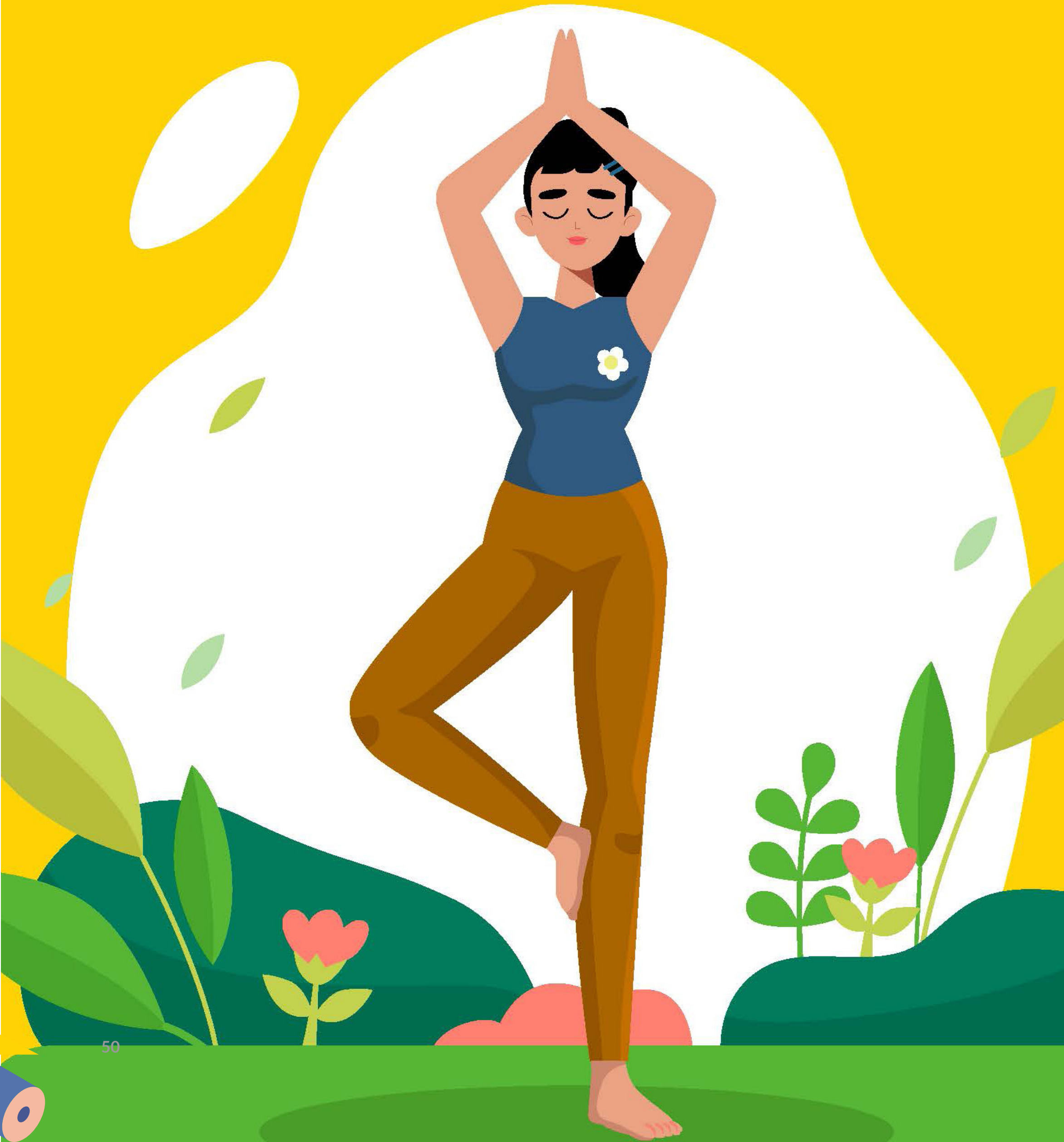

Perawatan Diri

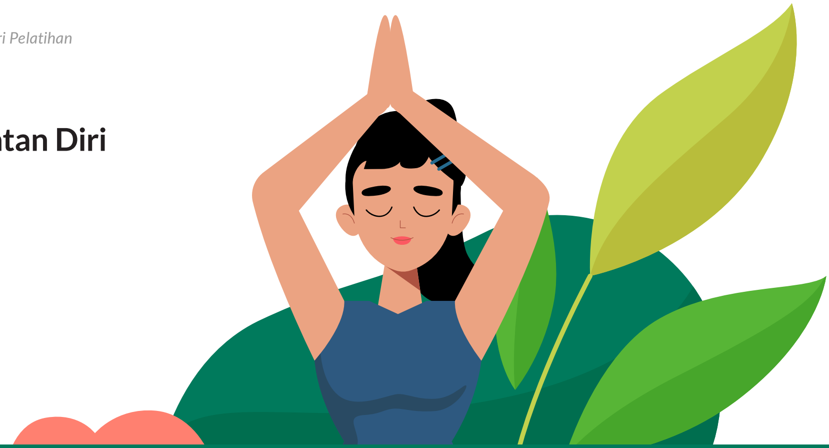

Stress Test: Siap Menghadapi Stres?

Salah satu cara paling efektif untuk meminimalkan stres yaitu menjaga diri sendiri. Cobalah baca beberapa pernyataan berikut dan checklist pernyataan yang mewakili gaya hidup Anda:

- Saya makan makanan seimbang setidaknya satu kali sehari
- Saya tidur tujuh hingga delapan jam sehari
- Saya mendapatkan kasih sayang dari orang sekitar
- Saya memiliki keluarga dekat yang dapat saya andalkan
- Saya berolahraga rutin, setidaknya dua kali dalam seminggu
- Saya tidak merokok
- Saya memiliki berat badan yang ideal
- Saya memiliki penghasilan yang cukup untuk memenuhi pengeluaran pokok saya dan keluarga
- Saya mendapatkan kekuatan dari keyakinan agama saya
- Saya rutin menghadiri kegiatan sosial di lingkungan saya
- Saya memiliki pertemanan yang luas
- Saya memiliki satu atau lebih teman yang dapat dijadikan tempat curhat mengenai masalah pribadi
- Saya mampu berbicara terbuka mengenai perasaan saya ketika marah atau khawatir
- Saya melakukan percakapan rutin dengan orang-orang yang tinggal bersama saya mengenai masalah rumah, pekerjaan, keuangan, dan kehidupan sehari-hari
- Saya melakukan sesuatu untuk bersenang-senang setidaknya satu kali dalam seminggu
- Saya dapat mengatur waktu saya dengan efektif
- Saya menyisihkan waktu untuk diri saya sendiri tanpa diganggu orang lain setiap harinya

Berapa banyak ceklis yang Anda miliki? Anda perlu mencentang lebih dari 50% kotak saat Anda sudah melengkapi formulir. Jika tidak, gaya hidup Anda tidak sepenuhnya membekali Anda keterampilan untuk mengatasi distres secara efektif

Kita Harus menjadi Penolong bagi Diri Kita Sendiri

Dalam keseharian, seringkali diri pendukung teman sebaya *(Peer Supporter)* seolah-olah diabaikan demi memenuhi pelayanan kepada orang lain. Kegagalan untuk mendengarkan apa yang terjadi di dalam diri kita, berlindung di bawah panji kepahlawanan, dapat meningkatkan distres dan memberikan beban yang berat. Hal ini juga membuat pendukung teman sebaya *(Peer Supporter)* rentan mengenali kebutuhan diri sendiri, sehingga aturan yang dicoba untuk diterapkan adalah pendukung teman sebaya *(Peer Supporter)* harus memperlakukan diri mereka seperti apa yang mereka lakukan pada orang lain, termasuk mendengarkan perasaan mereka sendiri dan menerimanya tanpa menghakimi.

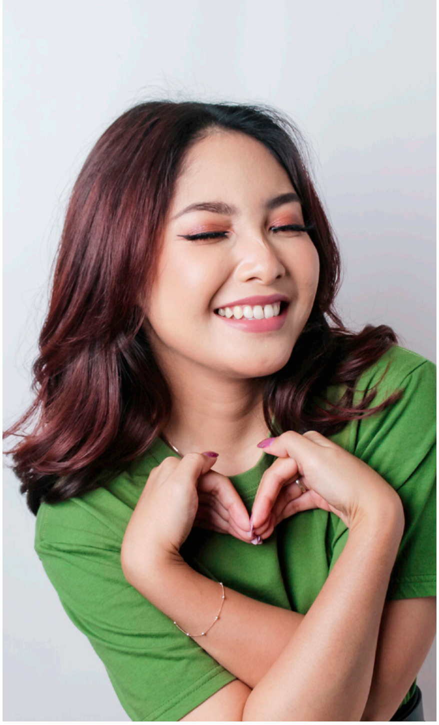

Caring for Yourself

1. Bersikap lembut pada diri sendiri. Ingatkan pada diri sendiri bahwa Anda adalah pendukung, bukan pesulap
2. Bangun jaringan dukungan Anda sendiri – keluarga, teman, kolega, dll.
3. Ubah rutinitas dan tugas Anda sesering mungkin.
4. Pelajari teknik relaksasi yang cocok untuk Anda dan ingatlah untuk menggunakannya.
5. Bersikaplah baik pada diri sendiri. Hadiahi diri Anda sendiri atas pekerjaan yang Anda lakukan.
6. Mengembangkan berbagai minat di luar jaringan dukungan teman sebaya.
7. Belajar mengenali perbedaan antara mengeluh yang meringankan dan mengeluh yang memperberat stres
8. Memberikan dukungan kepada teman sebaya (Peer Support). Belajarlah untuk menerimanya sebagai balasannya.
9. Ingatlah bahwa Anda tidak dapat mengubah orang lain. Anda hanya dapat mengubah cara Anda berhubungan yang lain.
10. Temukan tempat untuk menenangkan diri — gunakan setiap hari.
11. Fokuslah pada hal baik yang terjadi sepanjang hari.
12. Pastikan Anda mengonsumsi makanan yang sehat dan seimbang, sering berolahraga, dan cukup tidur.
13. Ingatlah bahwa diri Anda memiliki empat bagian penting (intelektual, emosional, spiritual dan fisik) yang perlu selalu dipupuk.
14. Beristirahatlah, jadwalkan jika diperlukan. Ingatlah untuk bersenang-senang selama waktu istirahat ini.

Referensi

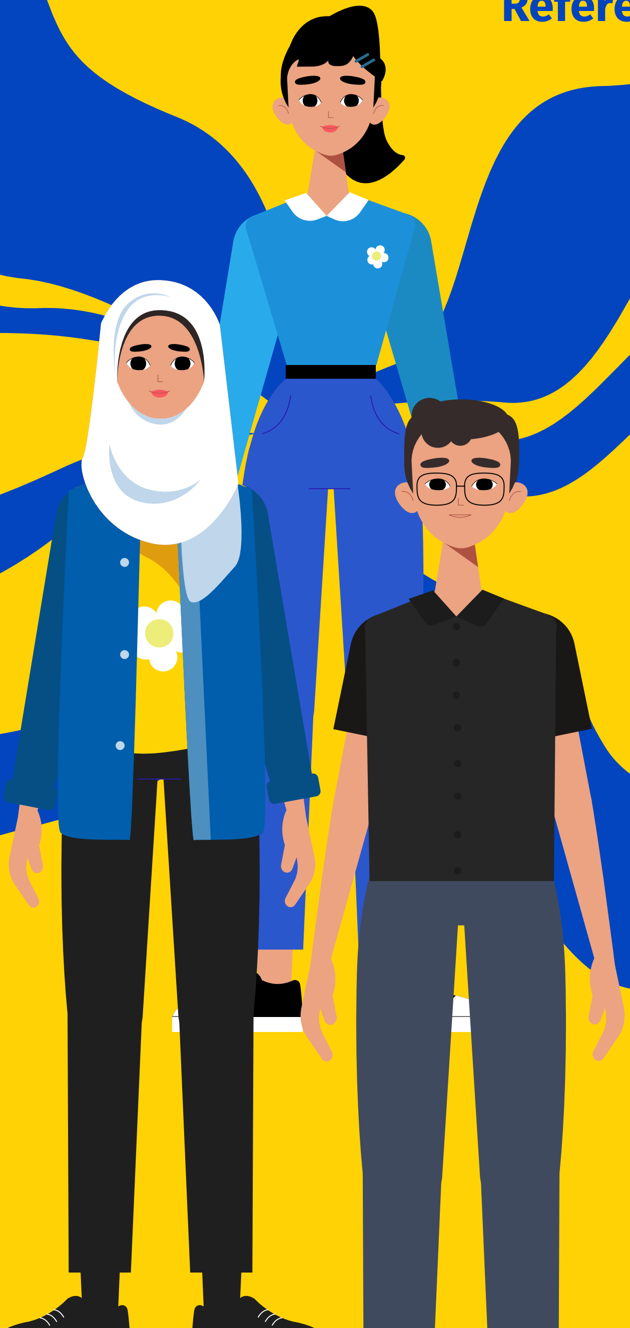

Core competencies for peer workers in Behavioral Health Services - Samhsa (no date) www.samhsa.gov. Available at: https://www.samhsa.gov/sites/default/files/programs_campaigns/brss_tacs/core-competencies_508_12_13_18.pdf (Accessed: 19 January 2024).

Direktorat Jenderal Pencegahan dan Pengendalian Penyakit, K. K. R. (2020). *Petunjuk Tenis Pendampingan Orang dengan TBC Resistan Obat Oleh Komunitas*.

Fuady, A. et al. (2024) 'Stigma, depression, quality of life, and the need for psychosocial support among people with tuberculosis in Indonesia: A multi-site cross-sectional study', PLOS Global Public Health, 4(1). doi:10.1371/journal.pgph.0002489.

Geldard, K. and Geldard, D. (2005) 'Useful Counseling Micro Skills', in Practical counselling skills : an integrative approach. Palgrave Macmillan, p. 130.

Ikatan Psikolog Klinis Indonesia. Prinsip Dasar Pelaksanaan Pendampingan Psikososial [Power point slides]

Ikatan Psikolog Klinis Indonesia. Deteksi Dini Permasalahan Psikologis [Power point slides]

Joo, J.H. et al. (2022) 'The benefits and challenges of established peer support programmes for patients, informal caregivers, and healthcare providers', Family Practice, 39(5), pp. 903–912. doi:10.1093/fampra/cmac004.

Kracen, A., Naughton, A., O'reilly, J., Panoutsakopoulou, V., & Rooney, N. (2003). *Peer Support Training Manual Written and compiled by Staff Members of the Student Counselling Service*. http://www.tcd.ie/Student_Counselling/

Menaldi, A., Hanum, L., & Asih, S. R. (n.d.). *Modul Penyegaran Keterampilan Memandu Support* Group. CEPAT-LKNU & Fakultas Psikologi UI.

UNIVERSITAS INDONESIA PUBLISHING

UNIVERSITAS INDONESIA
Veritas, Probitas, Iustitia
UI PUBLISHING

Jl Salemba Raya No 4, Jakarta Pusat 10430
0818 436500
E-mail: uipublishing@ui.ac.id
website: www.uipublishing.ui.ac.id

Komplek ILRC Gedung B Lt 1&2
Perpustakaan Lama Universitas Indonesia
Kampus UI Depok, Jawa Barat - 16424
Tel. + 62 21 7888 8199, 0812 9476 1054

ISBN 978-623-333-797-7
9 786233 337977
