# Supplementary material for: Community-based, peer-led psychosocial support to address stigma and reduce depression among adults with tuberculosis in Indonesia: A prospective interventional cohort study
Source: PLOS Glob Public Health. 2026 Jul 16;6(7):e0006754. doi: 10.1371/journal.pgph.0006754 (PMC13375138; doi:10.1371/journal.pgph.0006754)
Supplement: Attachment — Submitted filename: Report_Review_DK.docx [file pgph.0006754.s004.docx]

The manuscript is relevant and generally well written, but clarity can be improved—especially around defining project terms/acronyms and presenting the intervention steps more cleanly.

Major comments

Abstract & Methods: DR-TB intervention duration

- The abstract suggests DR-TB participants received the intervention only up to 6 months. Please clarify whether DR-TB participants truly received the intervention only for the first 6 months (given DR-TB treatment is often longer), and justify this choice if so.
- TB-CAPS not defined at first mention - “TB-CAPS” appears in the abstract without being written in full or defined, which may confuse readers encountering it for the first time. Please define TB-CAPS at first use (or avoid the acronym in the abstract if not essential).

Methods:

Sequencing/structure of intervention description is confusing

- In Lines 122–125, the “First… Second…” phrasing sets expectations for a clearly delimited list, but the narrative that follows makes it hard to track what belongs to each step. The appearance of “Finally…” later (around Line 214) further reinforces the sense that earlier steps are incomplete or that intervening text belongs to “Second.”
- Suggestion: reformat the intervention description into bullets or sub-steps, clearly separating (a) the intervention components and (b) evaluation activities.

Terminology assumes insider knowledge

- At Line 127, the term “active ingredients” is introduced without explanation and could be misinterpreted by non-specialist readers (e.g., as medicines/capsules). Please define the term at first use or replace with clearer wording such as “core components” or “intervention components.”

Positioning of TB-CAPS across the manuscript

- TB-CAPS is referenced repeatedly in a way that can make the paper read as though TB-CAPS is a standardized stigma intervention itself, rather than the broader project within which this study sits. Consider adding a short subheading/paragraph early (e.g., “About TB-CAPS”) to clearly state what TB-CAPS is, and what specific component/intervention this manuscript evaluates. After that, use “the intervention conducted” more consistently for readability.

Minor comments

- Line 247: “QoL” already used and abbreviated at first use in the main text
