# Supplementary material for: Community-based, peer-led psychosocial support to address stigma and reduce depression among adults with tuberculosis in Indonesia: A prospective interventional cohort study
Source: PLOS Glob Public Health. 2026 Jul 16;6(7):e0006754. doi: 10.1371/journal.pgph.0006754 (PMC13375138; doi:10.1371/journal.pgph.0006754)
Supplement: Attachment — Submitted filename: Overall Recommendation.docx [file pgph.0006754.s005.docx]

**Minor revision (acceptable after modest strengthening of Methods, Discussion, and Conclusions).**

**Summary**

This manuscript addresses an important and under-served aspect of TB care: reducing TB-related stigma and depressive symptoms through a community-based, peer-led psychosocial support package. The topic is timely and highly relevant to programmatic TB services. The study is clearly presented, uses locally adapted/validated tools, and demonstrates consistent improvement in stigma, depression, and quality of life across follow-up. With several targeted clarifications and modest strengthening of interpretation, the manuscript would be suitable for publication

**Major Points (focused revisions)**

**1) Methods(Intervention description and reproducibility)**

1. **Clarify the intervention logic model (theory of change).**
   Please add a brief conceptual pathway describing *how* each component (individual counselling, peer group counselling, online messaging, TB Talks) is expected to influence stigma, mental health, and QoL, and which components are hypothesized to be most influential.
2. **Provide slightly more operational detail to support replication.**
   Consider adding: core session topics/structure (even as a brief table or appendix), minimum “dose” intended (e.g., expected number of sessions), fidelity/quality assurance steps (supervision, checklists, refresher training).

**2) Methods/Analysis(Handling confounding and missing data)**

3. **Address potential confounding by clinical improvement during TB treatment.** Because symptoms commonly improve over time with TB therapy, please explicitly discuss in Methods/Discussion whether any clinical markers (e.g., treatment phase, symptom improvement proxy) were considered, and how this limitation affects attribution to the intervention.
4. **Missing data and attrition.** Please clarify: whether analyses were restricted to completers (complete-case approach), any differences between completers and non-completers (even descriptively), how missingness might bias estimates.
5. **Consider reporting effect sizes or clinically meaningful change.** Beyond p-values, adding simple effect size indicators (e.g., absolute risk reduction for “moderate-to-severe stigma” and “moderate-to-severe depression,” or median change with confidence intervals) would strengthen interpretability.

**3) Discussion(Strengthen causal framing and contextualization)**

6. **Strengthen the causal language and separate inference from hypothesis.** The manuscript appropriately notes the lack of a control group; however, some statements may still read as causal. Please ensure the Discussion consistently frames findings as associations over time within a single-arm cohort and highlights that causality cannot be established.
7. **Situate findings more deeply in the broader literature.** Please expand comparison with prior evidence on peer-led psychosocial interventions in TB and other infectious diseases, noting where this study extends the evidence base (community setting, mixed “active ingredients,” implementation feedback).
8. **Implementation and scalability.** The implementation challenges are important and should be made more actionable. Please add a short paragraph specifying: minimum requirements for scale-up (staffing/time, partnerships, supervision), practical scheduling solutions (e.g., after-hours/weekend groups), how referral pathways to mental health services could be integrated.

**4) Conclusions(Align strictly with design and add pragmatic recommendations)**

9. **Refine the Conclusions to reflect the study design.** Please avoid wording that may imply proven effectiveness. Consider revising to: “The intervention shows promise/potential and warrants evaluation in controlled designs.”
10. **Add 1–2 program-relevant takeaways.** For example: early-phase (intensive phase) counselling may be a feasible priority window; peer supporters can complement PHC services when adequately trained and supervised; integration into NTP activities is plausible with defined roles and budgeting.

**Data Availability (Policy alignment).** The Data Availability statement indicates data are not publicly available and are available on request due to privacy/ethical restrictions. Given PLOS data policy expectations, please clarify what level of de-identified data can be shared, and under what conditions (e.g., controlled access, data use agreement, restricted repository). Even if full open sharing is not feasible, providing a more detailed access pathway would improve compliance and transparency.

**Minor Points (editorial/clarity)**

1. Please ensure consistent terminology for “TB-Stigma” and categories (no/low vs moderate/high) and justify dichotomization briefly (loss of information vs analytic clarity).
2. Consider adding a short note on facilitator variability (peer supporters vs volunteers) and whether any standardization/fidelity checks were used.
3. If feasible, a brief appendix listing core counselling themes and examples of TB Talks content would enhance transferability.

**Final Assessment**

This is a valuable and timely contribution with clear public health relevance. The findings are coherent, the measures are appropriate, and the manuscript is generally well written. With the targeted additions above - primarily improving methodological clarity, strengthening causal framing, and refining conclusions -the paper would be suitable for publication.
