# Supplementary material for: Community-based, peer-led psychosocial support to address stigma and reduce depression among adults with tuberculosis in Indonesia: A prospective interventional cohort study
Source: PLOS Glob Public Health. 2026 Jul 16;6(7):e0006754. doi: 10.1371/journal.pgph.0006754 (PMC13375138; doi:10.1371/journal.pgph.0006754)
Supplement: Attachment — Submitted filename: Response to Reviewers PLOS GPH TW-FINAL TW.docx [file pgph.0006754.s006.docx]

Dr Tom Wingfield MbChB(Hons) PhD DTMH MRCP DipHIV DELE

Consultant Physician and Reader in Tuberculosis and Social Medicine

Deputy Director of the Centre for Tuberculosis Research, LIV-TB Lead

Departments of Clinical Sciences and International Public Health

Liverpool School of Tropical Medicine

Email: [tom.wingfield@lstmed.ac.uk](mailto:tom.wingfield@lstmed.ac.uk)

Website: <https://www.lstmed.ac.uk/about/people/dr-tom-wingfield>

17^th^ April 2026

**Subject: Response to reviewer comments on PLOS Global Public Health original research article PGPH-D-26-00317 entitled “Community-based, peer-led psychosocial support to address stigma and reduce depression among adults with tuberculosis in Indonesia: a prospective interventional cohort study”**

Dear Graeme and the PLoS Global Public Health Editorial Team,

Thank you very much to you and the editorial team and reviewers for your review of our manuscript and the recommendation of minor revision.

We have carefully addressed each of the points raised and made corresponding revisions to the manuscript where appropriate.

Below, we outline our detailed responses and indicate where changes have been made in the manuscript (indicated by *red italic text*).

We hope that these revisions have addressed the remaining concerns and that the manuscript is now suitable for publication in PLOS Global Public Health.

Please let us know if any further clarification or revisions would be helpful.

Yours sincerely,

Dr Tom Wingfield on behalf of the author team

**Editor Comments**

Dear Dr. Wingfield,

Thank you for submitting your manuscript to PLOS Global Public Health. After careful consideration, we feel that it has merit but does not fully meet PLOS Global Public Health’s publication criteria as it currently stands. Therefore, we invite you to submit a revised version of the manuscript that addresses the points raised during the review process.

Journal Requirements:

1. Some material included in your submission may be copyrighted. Please closely review the details of PLOS’s copyright requirements. Potential Copyright Issues: Figure 1: please (a) provide a direct link to the base layer of the map (i.e., the country or region border shape) and ensure this is also included in the figure legend; and (b) provide a link to the terms of use / license information for the base layer image or shapefile. We cannot publish proprietary or copyrighted maps (e.g. Google Maps, Mapquest) and the terms of use for your map base layer must be compatible with our CC-BY 4.0 license.

***Author response:*** We have ensured that the that Figure 1 complies with the copyright requirement. We added information of the base layer in the Figure legend. *“Figure 1. Study sites of TB-CAPS Study: Padang (West Sumatera) and Depok (West Java). The base layer of Indonesia map was created with mapchart.net (*[*Indonesia | MapChart*](https://www.mapchart.net/indonesia.html)*)”*

2. Please provide separate figure files in .tif or .eps format.

***Author Response:*** We have uploaded the Figures as .tiff files.

3. We have noticed that you have uploaded Supporting Information files, but you have not included a list of legends. Please add a full list of legends for your Supporting Information files after the references list.

***Author Response:*** We have now added a legend for our Supporting Information file at the end of the manuscript. “***S1.*** *Participants in Delphi survey and national participatory workshop (16-17 May 2026)”*

**Reviewer 1** **comments**

1. Abstract & Methods: DR-TB intervention duration. The abstract suggests DR-TB participants received the intervention only up to 6 months. Please clarify whether DR-TB participants truly received the intervention only for the first 6 months (given DR-TB treatment is often longer), and justify this choice if so.

***Author Response:*** Yes, people with DR-TB received the intervention up to the sixth month of their treatment due to limited follow-up time. From our previous implementation and evaluation experience in other contexts, including Peru and Nepal, we anticipate that psychological support will be most beneficial in the first two months of treatment when the psychological burden, including levels of depression, self-stigma, and anxiety, has been shown to be greatest. Therefore, we consider that follow-up to six months following treatment initiation is likely to be sufficient in this case, even for people with DR-TB. Moreover, within the limitations of this time-limited study and related grant period, it was not possible to continue follow up over variable, longer time periods depending on resistance profile and the treatment regimen received. In the Methods section, to respond to this helpful reviewer comment, we have updated a relevant section to read:

***Methods Section, Lines 205-210:*** *“It was expected that people with TB would attend two group counselling sessions during their initiation phase and could attend six in total during the intervention period for both people with DS- and DR-TB. The limited study and related grant period precluded our being able to intervene or follow-up for longer durations. Moreover, from our related research, we have demonstrated that levels of stigma and depression are highest in the treatment initiation phase rather than continuation phase, and so also assumed that psychological support would be most beneficial in the first two months and, therefore, follow-up up to six months would be sufficient.”*

2. TB-CAPS not defined at first mention - “TB-CAPS” appears in the abstract without being written in full or defined, which may confuse readers encountering it for the first time. Please define TB-CAPS at first use (or avoid the acronym in the abstract if not essential).

***Author Response:*** We agree with thanks. We have now revised the sentence by removing "TB-CAPS" and replacing it with *"activities in this study"* to keep the word count within the limits stipulated. (Line 57)

3. Methods: Sequencing/structure of intervention description is confusing. In Lines 122–125, the “First… Second…” phrasing sets expectations for a clearly delimited list, but the narrative that follows makes it hard to track what belongs to each step. The appearance of “Finally…” later (around Line 214) further reinforces the sense that earlier steps are incomplete or that intervening text belongs to “Second.” Suggestion: reformat the intervention description into bullets or sub-steps, clearly separating (a) the intervention components and (b) evaluation activities.

***Author Response:*** Thank you for your clear and helpful observation and suggestions. We have since updated the text to read *"…through three key activities"* (Line 122) and also added *"Third, we carried out…"* (Line 130) to make the progression narrative clearer. We have also added bullet points for each "active ingredient" of the intervention we describe to further delineate activities.

4. Methods: Terminology assumes insider knowledge. At Line 127, the term “active ingredients” is introduced without explanation and could be misinterpreted by non-specialist readers (e.g., as medicines/capsules). Please define the term at first use or replace with clearer wording such as “core components” or “intervention components.”

***Author Response:*** We added the definition of "active ingredients," a term introduced relatively recently, primarily in the mental health intervention literature and also in common usage by funders including Medical Research Council and [Wellcome Trust](chrome-extension://efaidnbmnnnibpcajpcglclefindmkaj/https:/cms.wellcome.org/sites/default/files/2022-11/Mental_Health_Active_Ingredients_table_Nov22.pdf). We have updated the text to read:

***Methods, Lines 126-128:*** *“…of the TB-CAPS “active ingredients” ­­­­­ —activities in our intervention that potentially drive clinical effect, are conceptually well defined, and link to specific hypothesised mechanisms of action[11]—…”*

5. Methods: Positioning of TB-CAPS across the manuscript. TB-CAPS is referenced repeatedly in a way that can make the paper read as though TB-CAPS is a standardized stigma intervention itself, rather than the broader project within which this study sits. Consider adding a short subheading/paragraph early (e.g., “About TB-CAPS”) to clearly state what TB-CAPS is, and what specific component/intervention this manuscript evaluates. After that, use “the intervention conducted” more consistently for readability.

***Author Response:*** Thank you for your suggestion, with which we agree. We removed "TB-CAPS" and replaced it with *"the intervention"* throughout the manuscript, retaining the term only where relevant.

6. Line 247: “QoL” already used and abbreviated at first use in the main text.

***Author Response:*** We replaced this with "QoL" and ensured that the abbreviation is used consistently after its first mention.

**Reviewer 2 Comments**

Major Points (focused revisions)

1) Methods(Intervention description and reproducibility)

1.	Clarify the intervention logic model (theory of change). Please add a brief conceptual pathway describing how each component (individual counselling, peer group counselling, online messaging, TB Talks) is expected to influence stigma, mental health, and QoL, and which components are hypothesized to be most influential.

***Author Response:*** Thank you for this helpful comment. We have now updated the text to describe the theory of change, which is from our previous scoping review and conceptual framework publications.

***Methods, lines 175-182:*** *“Based on the conceptual frameworks, which include pathways to impact, developed in our previous scoping review[5, 13], individual and group counselling are proposed to improve participants' knowledge and attitudes toward the disease, as well as foster a sense of psychological and social support, which in turn strengthens self-efficacy, reduces enacted stigma, and ultimately diminishes self-stigma and anticipated stigma. Complementarily, community conversations create a space where individuals feel seen and heard, promoting greater community understanding of tuberculosis and contributing to a reduction in enacted stigma.”*

2.	Provide slightly more operational detail to support replication. Consider adding: core session topics/structure (even as a brief table or appendix), minimum “dose” intended (e.g., expected number of sessions), fidelity/quality assurance steps (supervision, checklists, refresher training).

***Author Response:*** Thank you for your suggestions. We have now updated the detailed description concerning the *“active ingredients”* of our intervention in the Box. See also our related response to Reviewer 1.

2) Methods/Analysis(Handling confounding and missing data)

3.	Address potential confounding by clinical improvement during TB treatment. Because symptoms commonly improve over time with TB therapy, please explicitly discuss in Methods/Discussion whether any clinical markers (e.g., treatment phase, symptom improvement proxy) were considered, and how this limitation affects attribution to the intervention.

***Author Response:*** Despite the non-randomised interventional cohort study design, we were able to incorporate uniformity for some variables, including treatment phase since all participants were people newly diagnosed with TB. We also discussed in our findings about how symptom improvement may affect perceived stigma and mental health (Discussion, (lines 481-483). We have also clearly acknowledged the lack of a control arm as a limitation, which we would wish to address in future studies with randomised controlled trial design (Discussion, lines 561-569).

4.	Missing data and attrition. Please clarify: whether analyses were restricted to completers (complete-case approach), any differences between completers and non-completers (even descriptively), how missingness might bias estimates.

***Author Response:*** Yes, the analyses were restricted to completers in each measurement points (Month-2 and Month-6).

In the Methods, Lines 325-327, we added, *“We also applied complete-case approach, restricting the analyses to completers in each measurement point (Month-2 and Month-6).”*

Most participants not completing the intervention and follow-up were participants who were lost-to-follow-up the study at Month-2 (12/112), and only one more missing at Month-6. These missing cases may underestimate the reduction in stigma and depression.

We added a sentence to reflect this in the limitations paragraph as follows:

Discussion, Lines 573-576: “*Third, since we applied a complete-case approach in our analysis and there were some (13/112, 12%) missing cases, particularly at Month-2, the findings may not reflect the true changes in stigma and depression amongst all participants recruited, most likely leading to underestimation in their reductions.*”

5.	Consider reporting effect sizes or clinically meaningful change. Beyond p-values, adding simple effect size indicators (e.g., absolute risk reduction for “moderate-to-severe stigma” and “moderate-to-severe depression,” or median change with confidence intervals) would strengthen interpretability.

***Author Response:*** We thank the reviewer for this valuable suggestion. Our current approach to reporting results is consistent with our previous publications (Kritika et al. from Nepal [https://doi.org/10.1186/s44263-024-00049-2]; Fuady et al. from Indonesia [https://doi.org/10.1371/journal.pgph.0002489]), and we have maintained this format to ensure comparability and ease of interpretation across studies. We would therefore respectfully suggest retaining the current reporting approach. Additionally, it is worth noting that in the Indonesian context, there are as yet no well-validated benchmarks for minimally clinically important differences (MCID) for stigma and depression measures. Reporting effect sizes anchored to MCID thresholds would therefore risk implying a level of clinical interpretability that is not yet empirically established in this setting.

3) Discussion (Strengthen causal framing and contextualization)

6.	Strengthen the causal language and separate inference from hypothesis. The manuscript appropriately notes the lack of a control group; however, some statements may still read as causal. Please ensure the Discussion consistently frames findings as associations over time within a single-arm cohort and highlights that causality cannot be established.

***Author Response:*** We thank the reviewer for this important methodological and interpretation points. We have revised the Discussion throughout to ensure that all findings are consistently framed as associations observed over time within a single-arm cohort, and have removed any language that could imply causality. We agree that causal inference cannot be drawn from a non-randomised study design without a control group. We have also added explicit clarification in the Discussion to highlight this limitation. Looking ahead, we recognise the need for a randomised controlled trial to establish causality, and this is a direction we intend to pursue in future research.

7.	Situate findings more deeply in the broader literature. Please expand comparison with prior evidence on peer-led psychosocial interventions in TB and other infectious diseases, noting where this study extends the evidence base (community setting, mixed “active ingredients,” implementation feedback).

***Author Response:*** Thank you for this comment. We have since added an entirely new paragraph to address this and we think this has helped to improve the discussion.

***Discussion, Lines 495-503:*** “*There is limited evidence on the effectiveness of peer-led group counselling in reducing TB-Stigma and depression. Most existing interventions, such as cash transfers and peer counselling, have primarily focused on improving treatment adherence and clinical outcomes[23–25]. More robust evidence is available from the HIV/AIDS and leprosy fields[26–28]. Group counselling sessions led by peers or affected communities, conducted regularly, have been shown to empower individuals with HIV/AIDS by facilitating acceptance of their diagnosis and improving treatment adherence[26, 27]. In Indonesia, a study also found that multi-session individual counselling reduced stigma and depression among people affected by leprosy by enhancing their confidence[28].”*

8.	Implementation and scalability. The implementation challenges are important and should be made more actionable. Please add a short paragraph specifying: minimum requirements for scale-up (staffing/time, partnerships, supervision), practical scheduling solutions (e.g., after-hours/weekend groups), how referral pathways to mental health services could be integrated.

***Author Response:*** Thank you for your suggestions. To maintain ourselves within PLOS GPH’s specified maximum word limits, we have added a concise paragraph to explain potential implementation challenges.

***Discussion, Lines 546-560:*** *“The findings from our study suggest the potential for scale-up, although several issues need to be addressed to ensure the intervention is actionable and implementable. As highlighted in the participatory workshop, implementation should be integrated within existing systems to avoid siloing and fragmentation across specialties, particularly by linking with mental health services and established referral pathways. Training is required for TB-related healthcare workers at the primary care level, peer supporters, and general physicians. In addition, collaboration with TB survivor organizations and other relevant civil society organizations, should be strengthened. These organizations could receive training of trainers to enable them to train peer supporters, provide supervision, and help minimize the workload of primary care healthcare workers. Scheduling should remain flexible, with peer supporters identifying the most appropriate times for group counselling (e.g., weekends in urban settings, and after working hours on weekdays in rural areas). Another important issue is financing these activities; funding may be sourced from provincial or district health offices, with potential collaboration from philanthropic organizations.”*

4) Conclusions(Align strictly with design and add pragmatic recommendations)

9.	Refine the Conclusions to reflect the study design. Please avoid wording that may imply proven effectiveness. Consider revising to: “The intervention shows promise/potential and warrants evaluation in controlled designs.”

***Author Response:*** Thank you. As per our response regarding causality above, we have carefully revised the relevant wording accordingly here and throughout the manuscript.

10.	Add 1–2 program-relevant takeaways. For example: early-phase (intensive phase) counselling may be a feasible priority window; peer supporters can complement PHC services when adequately trained and supervised; integration into NTP activities is plausible with defined roles and budgeting.

***Author Response:*** Thank you for your suggestions. We have revised the Conclusion to be in line with this useful comment **(Lines 588-600)**. *“The psychosocial support intervention shows potential for reducing TB-Stigma and depressive symptoms and for improving QoL. However, a controlled study is needed to confirm its effectiveness. Peer-led group counselling during the intensive phase may represent a feasible priority window for further scale-up, and adequately trained and supervised peer supporters can complement Primary Health Centre services in delivering such psychosocial support. Participants and stakeholders found the intervention acceptable, appropriate, and feasible within existing TB services Scale-up and institutionalisation of integrated psychosocial support within national TB programmes would be achievable with clearly defined roles between healthcare workers and peer supporters, alongside sufficient and sustained funding.”*

11. Data Availability (Policy alignment). The Data Availability statement indicates data are not publicly available and are available on request due to privacy/ethical restrictions. Given PLOS data policy expectations, please clarify what level of de-identified data can be shared, and under what conditions (e.g., controlled access, data use agreement, restricted repository). Even if full open sharing is not feasible, providing a more detailed access pathway would improve compliance and transparency.

***Author Response:*** Thank you. We have since revised, in Section **Availability of data and materials, Lines 612-615**, this text to read *“The data of this study are not publicly available due to privacy or ethical restrictions but requests for the de-identified, aggregate dataset can be made to the corresponding author and this data may be made available depending on specific data use and transfer agreements.”*

Minor Points (editorial/clarity)

1. Please ensure consistent terminology for “TB-Stigma” and categories (no/low vs moderate/high) and justify dichotomization briefly (loss of information vs analytic clarity).

***Author Response:*** We have been careful to review and ensure that TB-Stigma is consistently used throughout. We have also added an explanation in the following text:

***Methods, Lines 262-265:*** *“There is no specific TB-Stigma category, but based on learning from our previous study, we divided the scores into three categories: no stigma (TB-Stigma score=0), low (≤16.67), moderate (16.68–33.33), and high (>33.33)[6].”*

2. Consider adding a short note on facilitator variability (peer supporters vs volunteers) and whether any standardization/fidelity checks were used.

***Author Response:*** Thank you. We have added the following text to clarify this issue:

***Methods, Lines 224-229:*** *“At least one team member observed the peer-led group counselling sessions and conducted debriefings with peer supporters and physicians afterward to evaluate the sessions, identify areas for refinement in subsequent sessions, and discuss topics for upcoming meetings. All facilitators received refresher training six months after the initial training to reinforce, evaluate, and further improve their skills.”*

3. If feasible, a brief appendix listing core counselling themes and examples of TB Talks content would enhance transferability.

***Author Response:*** We have added this detail in the Box.

***End of response. Thank you editors and reviewers for helping us to improve this research!***
