# Supplementary material for: Community-based, peer-led psychosocial support to address stigma and reduce depression among adults with tuberculosis in Indonesia: A prospective interventional cohort study
Source: PLOS Glob Public Health. 2026 Jul 16;6(7):e0006754. doi: 10.1371/journal.pgph.0006754 (PMC13375138; doi:10.1371/journal.pgph.0006754)
Supplement: Peer review history

# Peer review: Decision Letter 0

**DOI:** 10.1371/journal.pgph.0006754.r001

**Authors:** Graeme Hoddinott

23 Feb 2026

PGPH-D-26-00317

Community-based, peer-led psychosocial support to address stigma and reduce depression among adults with tuberculosis in Indonesia: a prospective interventional cohort study

PLOS Global Public Health

Dear Dr. Wingfield,

Thank you for submitting your manuscript to PLOS Global Public Health. After careful consideration, we feel that it has merit but does not fully meet PLOS Global Public Health’s publication criteria as it currently stands. Therefore, we invite you to submit a revised version of the manuscript that addresses the points raised during the review process.

EDITOR:

I agree with the reviewers' overall endorsement of the quality of the project / manuscript. They have offered some suggestions for minor revisions. These are generally reasonable, although I'm sure could be rebutted if you felt that there was sufficient justification. Please consider these and submit a revised version at your convenience.

Please note that you may need to open the attachment submitted by reviewer 2 to read the commentary in English.

Please submit your revised manuscript by Mar 25 2026 11:59PM. If you will need more time than this to complete your revisions, please reply to this message or contact the journal office at globalpubhealth@plos.org. When you're ready to submit your revision, log on to https://www.editorialmanager.com/pgph/ and select the 'Submissions Needing Revision' folder to locate your manuscript file.

Please include the following items when submitting your revised manuscript:

- A letter that responds to each point raised by the editor and reviewer(s). You should upload this letter as a separate file labeled 'Response to Reviewers'.
- A marked-up copy of your manuscript that highlights changes made to the original version. You should upload this as a separate file labeled 'Revised Manuscript with Track Changes'.
- An unmarked version of your revised paper without tracked changes. You should upload this as a separate file labeled 'Manuscript'.

Guidelines for resubmitting your figure files are available below the reviewer comments at the end of this letter.

We look forward to receiving your revised manuscript.

Kind regards,

Graeme Hoddinott, Ph.D

Academic Editor

PLOS Global Public Health

Journal Requirements:

1. Some material included in your submission may be copyrighted. According to PLOS’s copyright policy, authors who use figures or other material (e.g., graphics, clipart, maps) from another author or copyright holder must demonstrate or obtain permission to publish this material under the Creative Commons Attribution 4.0 International (CC BY 4.0) License used by PLOS journals. Please closely review the details of PLOS’s copyright requirements here: PLOS Licenses and Copyright. If you need to request permissions from a copyright holder, you may use PLOS's Copyright Content Permission form.

Please respond directly to this email or email the journal office and provide any known details concerning your material's license terms and permissions required for reuse, even if you have not yet obtained copyright permissions or are unsure of your material's copyright compatibility.

Potential Copyright Issues:

Figure 1: please (a) provide a direct link to the base layer of the map (i.e., the country or region border shape) and ensure this is also included in the figure legend; and (b) provide a link to the terms of use / license information for the base layer image or shapefile. We cannot publish proprietary or copyrighted maps (e.g. Google Maps, Mapquest) and the terms of use for your map base layer must be compatible with our CC-BY 4.0 license.

Note: if you created the map in a software program like R or ArcGIS, please locate and indicate the source of the basemap shapefile onto which data has been plotted.

If your map was obtained from a copyrighted source please amend the figure so that the base map used is from an openly available source. Alternatively, please provide explicit written permission from the copyright holder granting you the right to publish the material under our CC-BY 4.0 license.

Please note that the following CC BY licenses are compatible with PLOS license: CC BY 4.0, CC BY 2.0 and CC BY 3.0, meanwhile such licenses as CC BY-ND 3.0 and others are not compatible due to additional restrictions.

If you are unsure whether you can use a map or not, please do reach out and we will be able to help you. The following websites are good examples of where you can source open access or public domain maps:

* U.S. Geological Survey (USGS) - All maps are in the public domain. (http://www.usgs.gov)

* PlaniGlobe - All maps are published under a Creative Commons license so please cite “PlaniGlobe, http://www.planiglobe.com, CC BY 2.0” in the image credit after the caption. (http://www.planiglobe.com/?lang=enl)

* Natural Earth - All maps are public domain. (http://www.naturalearthdata.com/about/terms-of-use/)

2. Please provide separate figure files in .tif or .eps format.

For more information about figure files please see our guidelines:  LINK

https://journals.plos.org/globalpublichealth/s/figures

https://journals.plos.org/globalpublichealth/s/figures#loc-file-requirements

3. We have noticed that you have uploaded Supporting Information files, but you have not included a list of legends. Please add a full list of legends for your Supporting Information files after the references list.

If the reviewer comments include a recommendation to cite specific previously published works, please review and evaluate these publications to determine whether they are relevant and should be cited. There is no requirement to cite these works unless the editor has indicated otherwise.

Please review your reference list to ensure that it is complete and correct. If you have cited papers that have been retracted, please include the rationale for doing so in the manuscript text, or remove these references and replace them with relevant current references. Any changes to the reference list should be mentioned in the rebuttal letter that accompanies your revised manuscript. If you need to cite a retracted article, indicate the article’s retracted status in the References list and also include a citation and full reference for the retraction notice.

Additional Editor Comments (if provided):

[Note: HTML markup is below. Please do not edit.]

Reviewers' comments:

Reviewer's Responses to Questions

**Comments to the Author**

1. Does this manuscript meet PLOS Global Public Health’s publication criteria? Is the manuscript technically sound, and do the data support the conclusions? The manuscript must describe methodologically and ethically rigorous research with conclusions that are appropriately drawn based on the data presented.

Reviewer #1: Yes

Reviewer #2: Yes

**********

2. Has the statistical analysis been performed appropriately and rigorously?

Reviewer #1: Yes

Reviewer #2: Yes

**********

3. Have the authors made all data underlying the findings in their manuscript fully available (please refer to the Data Availability Statement at the start of the manuscript PDF file)?

The PLOS Data policy requires authors to make all data underlying the findings described in their manuscript fully available without restriction, with rare exception. The data should be provided as part of the manuscript or its supporting information, or deposited to a public repository. For example, in addition to summary statistics, the data points behind means, medians and variance measures should be available. If there are restrictions on publicly sharing data—e.g. participant privacy or use of data from a third party—those must be specified.

Reviewer #1: Yes

Reviewer #2: No

**********

4. Is the manuscript presented in an intelligible fashion and written in standard English?

PLOS Global Public Health does not copyedit accepted manuscripts, so the language in submitted articles must be clear, correct, and unambiguous. Any typographical or grammatical errors should be corrected at revision, so please note any specific errors here.

Reviewer #1: Yes

Reviewer #2: Yes

**********

5. Review Comments to the Author

Please use the space provided to explain your answers to the questions above. You may also include additional comments for the author, including concerns about dual publication, research ethics, or publication ethics. (Please upload your review as an attachment if it exceeds 20,000 characters)

Reviewer #1: The manuscript is relevant and generally well written, but clarity can be improved—especially around defining project terms/acronyms and presenting the intervention steps more cleanly.

Major comments

Abstract & Methods: DR-TB intervention duration

• The abstract suggests DR-TB participants received the intervention only up to 6 months. Please clarify whether DR-TB participants truly received the intervention only for the first 6 months (given DR-TB treatment is often longer), and justify this choice if so.

• TB-CAPS not defined at first mention - “TB-CAPS” appears in the abstract without being written in full or defined, which may confuse readers encountering it for the first time. Please define TB-CAPS at first use (or avoid the acronym in the abstract if not essential).

Methods:

Sequencing/structure of intervention description is confusing

• In Lines 122–125, the “First… Second…” phrasing sets expectations for a clearly delimited list, but the narrative that follows makes it hard to track what belongs to each step. The appearance of “Finally…” later (around Line 214) further reinforces the sense that earlier steps are incomplete or that intervening text belongs to “Second.”

• Suggestion: reformat the intervention description into bullets or sub-steps, clearly separating (a) the intervention components and (b) evaluation activities.

Terminology assumes insider knowledge

• At Line 127, the term “active ingredients” is introduced without explanation and could be misinterpreted by non-specialist readers (e.g., as medicines/capsules). Please define the term at first use or replace with clearer wording such as “core components” or “intervention components.”

Positioning of TB-CAPS across the manuscript

• TB-CAPS is referenced repeatedly in a way that can make the paper read as though TB-CAPS is a standardized stigma intervention itself, rather than the broader project within which this study sits. Consider adding a short subheading/paragraph early (e.g., “About TB-CAPS”) to clearly state what TB-CAPS is, and what specific component/intervention this manuscript evaluates. After that, use “the intervention conducted” more consistently for readability.

Minor comments

• Line 247: “QoL” already used and abbreviated at first use in the main text

Reviewer #2: Представленная рукопись посвящена чрезвычайно актуальной и социально значимой проблеме — снижению стигмы и депрессивных симптомов у людей с туберкулёзом посредством общественно-ориентированной, peer-led психосоциальной интервенции. В целом работа производит впечатление методологически добросовестного, этически корректного и хорошо спланированного исследования, а также представляет практическую ценность для систем здравоохранения, особенно в странах с высоким бременем ТБ.

Дизайн исследования (проспективное однофакторное интервенционное когортное) описан чётко и прозрачно. Критерии включения и исключения обоснованы, процесс набора участников и ход интервенции изложены достаточно подробно, что обеспечивает воспроизводимость подхода. Особенно положительно следует отметить использование валидированных и локально адаптированных инструментов измерения (Van Rie TB-Stigma scale, PHQ-9, EQ-5D-5L), а также многократные точки измерения (baseline, Month-2, Month-6), что позволяет оценить динамику показателей во времени.

Статистический анализ в целом соответствует типу данных и выбранному дизайну исследования. Применение тестов McNemar и Cochran’s Q для анализа изменений при повторных измерениях является уместным и методологически оправданным. Авторы представляют p-значения, описывают распределения, медианы и межквартильные интервалы, а также корректно интерпретируют полученные эффекты. Важно, что в разделе обсуждения авторы честно и корректно указывают ключевые ограничения исследования, прежде всего отсутствие контрольной группы и, как следствие, невозможность строгого установления причинно-следственной связи. Такая взвешенная интерпретация повышает доверие к результатам и демонстрирует высокий уровень научной добросовестности.

Выводы в целом соответствуют представленным данным и сформулированы осторожно, без чрезмерных обобщений. Авторы не утверждают окончательную доказанность эффективности, а говорят о «potential» и необходимости дальнейших контролируемых исследований, что выглядит научно корректно и уместно в контексте данного дизайна.

С этической точки зрения исследование выполнено надлежащим образом: получены соответствующие одобрения этических комитетов, описана процедура информированного согласия, отдельно отражены вопросы работы с уязвимой группой пациентов. Признаков нарушений исследовательской или публикационной этики, а также признаков двойной публикации в представленной версии рукописи не выявлено.

В то же время, отдельного внимания требует вопрос доступности данных. В разделе Data Availability указано, что данные не находятся в открытом доступе и предоставляются только по запросу в связи с этическими и конфиденциальными ограничениями. Это означает, что полный массив данных, лежащих в основе расчётов (за пределами сводной статистики), не является публично доступным, что формально не полностью соответствует политике PLOS по открытости данных. С учётом чувствительности информации и уязвимости исследуемой группы такие ограничения понятны и во многом обоснованы, однако авторам, возможно, стоит более чётко обозначить, какие именно данные могут быть предоставлены в деидентифицированном виде или при каких условиях доступ возможен, чтобы лучше соответствовать требованиям журнала.

С точки зрения языка и стиля, рукопись написана на хорошем, стандартном научном английском языке. Текст логично структурирован, последователен и в целом легко читается. Серьёзных языковых или стилистических проблем, препятствующих пониманию, не выявлено. Возможны лишь незначительные редакторские и стилистические правки на этапе доработки, но они носят косметический характер и не влияют на научное содержание.

В целом работа представляет собой ценный вклад в область интеграции психосоциальной поддержки в программы борьбы с туберкулёзом, особенно в контексте первичной медико-санитарной помощи и участия сообщества. Исследование демонстрирует практическую реализуемость, приемлемость и потенциальную эффективность предложенной модели и заслуживает публикации после учёта замечаний, прежде всего касающихся политики доступа к данным и возможных уточнений в этом разделе.

**********

6. PLOS authors have the option to publish the peer review history of their article (what does this mean?). If published, this will include your full peer review and any attached files.

**Do you want your identity to be public for this peer review?**  If you choose “no”, your identity will remain anonymous but your review may still be made public.

For information about this choice, including consent withdrawal, please see our Privacy Policy.

Reviewer #1: No

Reviewer #2: No

**********

[NOTE: If reviewer comments were submitted as an attachment file, they will be attached to this email and accessible via the submission site. Please log into your account, locate the manuscript record, and check for the action link "View Attachments". If this link does not appear, there are no attachment files.]

Figure Resubmissions:

While revising your submission, we strongly recommend that you use PLOS’s NAAS tool (https://ngplosjournals.pagemajik.ai/artanalysis) to test your figure files. NAAS can convert your figure files to the TIFF file type and meet basic requirements (such as print size, resolution), or provide you with a report on issues that do not meet our requirements and that NAAS cannot fix.

After uploading your figures to PLOS’s NAAS tool - https://ngplosjournals.pagemajik.ai/artanalysis, NAAS will process the files provided and display the results in the "Uploaded Files" section of the page as the processing is complete. If the uploaded figures meet our requirements (or NAAS is able to fix the files to meet our requirements), the figure will be marked as "fixed" above. If NAAS is unable to fix the files, a red "failed" label will appear above. When NAAS has confirmed that the figure files meet our requirements, please download the file via the download option, and include these NAAS processed figure files when submitting your revised manuscript.

Attachment Submitted filename: Report_Review_DK.docx

Attachment Submitted filename: Overall Recommendation.docx

# Author comment: Author response to Decision Letter 1

**DOI:** 10.1371/journal.pgph.0006754.r002

1 May 2026

Attachment Submitted filename: Response to Reviewers PLOS GPH TW-FINAL TW.docx

# Editor report: Decision Letter 1

**DOI:** 10.1371/journal.pgph.0006754.r003

**Authors:** Graeme Hoddinott

10 Jun 2026

Community-based, peer-led psychosocial support to address stigma and reduce depression among adults with tuberculosis in Indonesia: a prospective interventional cohort study

PGPH-D-26-00317R1

Dear Dr. Wingfield,

We are pleased to inform you that your manuscript 'Community-based, peer-led psychosocial support to address stigma and reduce depression among adults with tuberculosis in Indonesia: a prospective interventional cohort study' has been provisionally accepted for publication in PLOS Global Public Health.

Before your manuscript can be formally accepted you will need to complete some formatting changes, which you will receive in a follow up email. A member of our team will be in touch with a set of requests.

Please note that your manuscript will not be scheduled for publication until you have made the required changes, so a swift response is appreciated.

IMPORTANT: The editorial review process is now complete. PLOS will only permit corrections to spelling, formatting or significant scientific errors from this point onwards. Requests for major changes, or any which affect the scientific understanding of your work, will cause delays to the publication date of your manuscript.

If your institution or institutions have a press office, please notify them about your upcoming paper to help maximize its impact. If they'll be preparing press materials, please inform our press team as soon as possible -- no later than 48 hours after receiving the formal acceptance. Your manuscript will remain under strict press embargo until 2 pm Eastern Time on the date of publication. For more information, please contact globalpubhealth@plos.org.

Thank you again for supporting Open Access publishing; we are looking forward to publishing your work in PLOS Global Public Health.

Best regards,

Graeme Hoddinott, Ph.D

Academic Editor

PLOS Global Public Health

***********************************************************

Reviewer Comments (if any, and for reference):
